# TrxR2 Lactylation Facilitates Mitochondrial Protection and Endothelial Ferroptosis Resistance in Diabetic Cardiomyopathy

**DOI:** 10.1002/advs.202521997

**Published:** 2026-02-17

**Authors:** Su Li, Muyin Liu, Chao Chen, Xinyan Li, Xiaopei Yan, Wentao Zhu, Wenyan Qiu, Qiyu Li, Xiangyu Sun, Chao Huang, Ming Yin, Zhangwei Chen, Yao Lu, Junbo Ge, Xiangqing Kong, Juying Qian, Yuqiong Chen

**Affiliations:** ^1^ Department of Cardiology, Zhongshan Hospital, Fudan University National Clinical Research Center for Interventional Medicine Shanghai P. R. China; ^2^ Department of Cardiology, The Affiliated Suzhou Hospital of Nanjing Medical University, Suzhou Municipal Hospital Gusu School Suzhou P. R. China; ^3^ Center for Precision Cancer Medicine & Translational Research Tianjin Medical University Cancer Institute and Hospital, National Clinical Research Center for Cancer, Key Laboratory of Cancer Prevention and Therapy, Tianjin's Clinical Research Center for Cancer Tianjin P. R. China; ^4^ Department of Respiratory Medicine, The Affiliated Suzhou Hospital of Nanjing Medical University, Suzhou Municipal Hospital Gusu School Suzhou P. R. China; ^5^ Ministry of Science and Technology, the Affiliated Suzhou Hospital of Nanjing Medical University Suzhou Municipal Hospital Suzhou Jiangsu P. R. China; ^6^ XuZhou Clinical School of Xuzhou Medical University, Department of Cardiology, Xuzhou Central Hospital XuZhou Institute of Cardiovascular disease Xuzhou P. R. China; ^7^ Department of Cardiology, Gulou District the First Affiliated Hospital of Nanjing Medical University Nanjing City Jiangsu Province P. R. China

**Keywords:** diabetic cardiomyopathy, ferroptosis, Lactylation modification, mitophagy, TrxR2

## Abstract

Thioredoxin reductase 2 (TrxR2), a radical‐trapping antioxidant, plays a critical role in cardiac defense. However, the mechanisms underlying its benefits remain unclear. In this study, we aimed to investigate whether endothelial TrxR2 prevents cardiac microvascular dysfunction in diabetic cardiomyopathy (DCM). Key genes in the thioredoxin family and those involved in ferroptosis were analyzed using bulk RNA‐sequencing assay. Diabetic injury was induced in multiple transgenic mouse models, including endothelial cell‐specific knockout mice for TrxR2, sterol carrier protein 2 (SCP2), and Tu translation elongation factor, mitochondrial (TUFM). The TrxR2 lactylation site was identified by mass spectrometry and verified by a custom‐made lactylation antibody. Mitochondrial thioredoxin reductase (mitoTrxR) activity and lipid peroxyl radicals were detected using fluorescence staining. Endothelial TrxR2 deficiency significantly suppressed mitoTrxR activity, exacerbated cardiac microvascular dysfunction, and accelerated DCM progression. In contrast, TrxR2 overexpression and Kukoamine B (TrxR2 agonist) treatment inhibited mitochondria‐associated ferroptosis by facilitating SCP2 degradation and blocking the mitochondrial translocation of acyl‐CoA synthetase long‐chain family member 4 (ACSL4) via mitophagy. Mechanistically, TrxR2 maintained TUFM expression by scavenging oxygen radicals, thereby facilitating the mitochondrial translocation of AMPK for mitophagy activation. TrxR2 undergoes lactylation at lysine 340. This process is mediated by mitochondrial alanyl‐tRNA synthetase 2 (AARS2) and lactate accumulation in both human and mouse diabetic hearts. This modification and sodium lactate administration compensatorily enhanced mitoTrxR activity, promoted mitophagy, and conferred ferroptosis resistance in cardiac microcirculation in DCM. Our findings demonstrate that TrxR2 and its lactylation modification promote mitophagy, enhance ferroptosis resistance, and improve cardiac microvascular function in DCM. Thus, this study provides a promising therapeutic approach for the management of diabetic complications.

## Graphical abstract

TrxR2 deletion in diabetic mice suppresses TUFM‐AMPK‐FUNDC1‐dependent mitophagy in endothelial cells, resulting in SCP2 upregulation and mitochondrial translocation of ACSL4. Mitochondrial ACSL4 promotes mitochondrial eicosanoid biosynthesis and ferroptosis, thereby aggravating cardiac microvascular injury and diabetic cardiomyopathy. Lactylation modification of TrxR2 in diabetes compensatorily enhances mitochondrial thioredoxin reductase activity, mitophagy, and ferroptosis resistance in endothelial cells, exerting cardiovascular protection.

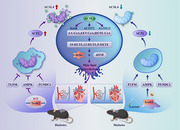

**Abbreviations**
AAV: Adeno‐associated virus
aAMPKα1: activated AMPKα1
aMitoAα1: activated mitochondrial translocation peptide‐fused AMPKα1
ACSL4: Acyl‐CoA synthetase long chain family member 4
Clpp: Caseinolytic mitochondrial matrix peptidase proteolytic subunit
CMA: Chaperone‐mediated autophagy
DCM: Diabetic cardiomyopathy
DHODH: Dihydroorotate dehydrogenase (Quinone)
FUNDC1: FUN14 domain containing 1
FSP1: Ferroptosis suppressor protein 1
GPX4: Glutathione peroxidase 4
HCMECs: Human cardiac microvascular endothelial cells
HFD/STZ: High‐fat diet feeding combined with streptozotocin injection
HG/PA: and H/P High glucose and palmitic acid treatment
K340: Lysine 340
Kuk B: Kukoamine B
LPO: Lipid peroxidation
MCMECs: Mouse cardiac microvascular endothelial cells
mitoLPO: Mitochondrial lipid peroxide
mitoTrxR: Mitochondrial thioredoxin reductase
MTP: Mitochondrial translocation peptide
Pan‐Kla: Pan‐lysine lactylation
PUFAs: polyunsaturated fatty acids
SCP2: sterol carrier protein 2
TUFM: Tu translation elongation factor, mitochondrial

## 1 Introduction

Diabetes mellitus accelerates atherosclerotic cardiovascular diseases, thereby contributing to high global morbidity, mortality, and financial burden [1, 2]. Diabetes triggers angina and cardiac dysfunction even in the absence of plaque formation or stenosis, primarily due to coronary microvascular endothelial dysfunction [3, 4]. However, clinically effective treatments for endothelial dysfunction remain limited. This has prompted further investigations into the mechanisms underlying microvascular dysfunction.

Mitochondrial dysfunction is the fundamental mechanism by which diabetes accelerates endothelial cell (EC) senescence, inhibits endothelial proliferation, and induces regulated cell death, including apoptosis, ferroptosis, and necroptosis [5, 6, 7, 8]. Mitochondria‐associated ferroptosis predominates in ECs; it exacerbates coronary microvascular dysfunction and diabetic cardiomyopathy (DCM) [6, 7]. Traditionally, the cysteine/cystine–GSH–GPX4 axis neutralizes lipid peroxyl radicals to suppress mitochondrial ferroptosis [9, 10]. Conversely, acyl‐CoA synthetase long‐chain family member 4 (ACSL4) preferentially incorporates long‐chain polyunsaturated fatty acids (PUFAs) into phospholipids via acyl‐CoA, consequently promoting lipid peroxidation (LPO) and contributing to various cardiomyopathies [11, 12, 13]. Cytoplasmic ACSL4 translocates to the mitochondria during diabetes, thereby aggravating mitochondria‐associated ferroptosis in ECs [6, 14]. However, it remains unclear why ACSL4 preferentially targets mitochondrial injury.

Dysfunctional mitochondria release excess reactive oxygen species (ROS), ions, and metabolites that drive ferroptosis. Mitophagy, which regulates mitochondrial turnover, has been proposed as a therapeutic approach [15]. However, mitophagy acts as a “double‐edged sword” in ferroptosis: it can reduce ROS and LPO by preserving mitochondrial integrity or promote ferroptosis by releasing free fatty acids and ferrous iron, which increase LPO [14, 16, 17, 18, 19]. Similarly, FUNDC1‐dependent mitophagy appears to play a paradoxical role in ferroptosis. FUNDC1 promotes hepatic ferroptosis through mitophagy‐mediated degradation of mitochondrial GPX4 [20]. In contrast, FUNDC1 overexpression reduces mitochondrial ROS production and LPO in endothelial ferroptosis induced by neutrophil extracellular traps [21]. More importantly, its insufficiency increases sensitivity to ACSL4‐mediated ferroptosis in high‐fat diet (HFD)‐induced cardiomyopathy, a metabolic disorder similar to DCM [22]. This discrepancy in mitophagy during ferroptosis may be context‐dependent. Elucidation of these disparate mechanisms may provide more precise strategies for targeting mitophagy in ferroptosis‐related diseases.

The thioredoxin superfamily is a highly conserved system that modulates the cellular redox state by reducing oxidized proteins through thiol–disulfide exchange reactions. It generally comprises NADPH, thioredoxins, thioredoxin reductases, and peroxiredoxins (Prdxs) [23]. Most of these have been introduced to delay diabetic complications by improving antioxidant activity [6, 24, 25]. The mitochondrial thioredoxins /Prdxs system, a subgroup of this superfamily, encompasses Trx2, TrxR2, and Prdx3, which maintain mitochondrial homeostasis and cardiac development [26, 27, 28]. Cardiomyocyte‐specific TrxR2 knockout induces mitochondrial ROS production and metabolic dysfunction to promote age‐related heart failure and ischemia‐reperfusion injury [29, 30]. In contrast, TrxR2 overexpression prevents doxorubicin‐induced cardiomyopathy [31]. Importantly, TrxR2 can reduce the oxidized form of Trx2, which alleviates DCM by reducing mitochondrial oxidative damage in cardiomyocytes [32]. Because its paralog (TrxR1) and downstream signaling protein (Prdx3) have been identified as ferroptosis markers, the role of TrxR2 in mitochondrial protection and ferroptosis warrants further exploration in DCM [33, 34].

Lactylation is a recently discovered, evolutionarily conserved post‐translational modification [35]. This process involves the covalent attachment of a lactyl group derived from the metabolite lactate to the lysine residues of proteins [35]. Lactylation uses lactyl‐CoA as a donor and is directly catalyzed by lactyltransferases, including alanyl‐tRNA synthetases 1 (AARS1) and 2 (AARS2) [36]. Elevated intracellular lactate levels, particularly under conditions of enhanced glycolysis such as the Warburg effect, significantly promote lactylation [37]. This directly links the cellular metabolic state to the regulation of protein functions. Lactate supplementation and α‐MHC K1897 lactylation alleviate Angiotensin II‐induced cardiomyocyte hypertrophy and heart failure [38]. In addition, Serpina3k lactylation at K351 in fibroblasts protects against cardiac ischemia‐reperfusion injury by suppressing cardiomyocyte apoptosis. As diabetes increases endothelial aerobic glycolysis and lactate content, whether lactylation modification affects mitochondrial thioredoxin reductase (mitoTrxR) activity remains unclear.

In the present study, we aimed to evaluate the benefits of TrxR2 in diabetes‐induced endothelial dysfunction, with a focus on mitochondrial homeostasis, mitophagy, and mitochondria‐associated ferroptosis. More exactly, how lactate orchestrates TrxR2 on the inhibition of endothelial ferroptosis is highlighted.

## 2 Methods

### 2.1 Human Samples

This study was conducted in accordance with the guidelines of the Declaration of Helsinki. Formalin‐fixed, paraffin‐embedded human heart slices from decedents with (diabetes group, *n* = 6) or without (control group, *n* = 6) diabetes were obtained from Zhongshan Hospital and Fudan University. The study protocol was approved by the Research Ethics Committee (approval Nos. B2014‐084, and 2020‐009) [39, 40, 41]. Human blood was collected in accordance with the guidelines of the Ethics Committee of Suzhou Municipal Hospital (approval No: K‐2024‐158‐K01). After obtaining informed consent, 8 mL blood was collected from 46 healthy adult volunteers and 81 patients with type 2 diabetes. Peripheral venous blood samples were collected in heparinized BD Vacutainer tubes and promptly transported to the laboratory.

### 2.2 Animals

All animal experiments were approved by the Animal Experimental Ethics Committee (approval Nos: 2023–029, and 2024–070) and performed in accordance with the animal experiment guidelines of Nanjing Medical University and Fudan University. C57BLKS/J^db/db^ (db/db) mice automatically develop type 2 diabetes mellitus (T2DM) and coronary microvascular dysfunction [42]. Four‐week‐old male db/db and littermate control (db/m) mice were purchased from M.Q. MICROBE Co., Ltd. (Suzhou, China) and housed in specific pathogen‐free rooms under standard conditions. Adeno‐associated virus serotype 9 (AAV9) packaging plasmids that encode full‐length TrxR2, TrxR2 K340R mutant, constitutively active AMPKα1(aAMPKα1), or aAMPKα1 with a mitochondrial transit peptide (aMitoAα1) were designed and constructed by QEgene (Shanghai, China). These plasmids were fused with the Tie2 promoter and a 3 × Flag tag to achieve endothelial specific transfection [6]. In total, 6 × 10^11^ genome copies were intravenously injected via the tail vein every 8 weeks for 24 weeks. The transfection efficiency and specificity of AAV9 were confirmed by immunofluorescence.

Global FUNDC1 knockout (FUNDC1‐/‐), SCP2^flox/flox^, endothelial‐specific SCP2 conditional knockout (SCP2^EC‐KO^), TUFM^flox/flox^, and endothelial‐specific TUFM conditional knockout (TUFM^EC‐KO^) mice were generated and purchased from M.Q. MICROBE Co., Ltd. (Suzhou, China). Homozygous genes were identified using polymerase chain reaction (PCR) and sequencing. Tamoxifen (50 mg/kg) dissolved in corn oil was intraperitoneally injected into SCP2^flox/flox^, SCP2^EC‐KO^, TUFM^flox/flox^, and TUFM^EC‐KO^ mice for 5 days. Male mice were fed an HFD for 4 weeks and intraperitoneally injected with three doses of streptozotocin (STZ; 40 mg/kg; Sigma‐Aldrich, St Louis, MO, USA) to establish the diabetic models [39]. Alda‐1 (10 mg/kg per day) was intraperitoneally injected for 24 weeks to improve ALDH2 activity [43]. FX‐11 (2 mg/kg per day) was intraperitoneally injected for 24 weeks to inhibit LDHA activity [44]. Sodium lactate (NaLa; 50 mg/kg per day) was intraperitoneally injected for 24 weeks to supplement exogenous lactate [38, 45].

### 2.3 Echocardiography

Mice were placed in the supine position and anesthetized with 2% isoflurane (RWD, China). Echocardiography was performed using a 30 MHz transducer to acquire M‐mode and Color Doppler flow images using the VisualSonics Vevo2100 system (VisualSonics, Canada). Cardiac function parameters, including left ventricular ejection fraction (LVEF), left ventricular fractional shortening (LVFS), and the ratio of early to late mitral flow velocities (E/A ratio), were analyzed using VevoLAB software (version 2.2.3; VisualSonics).

### 2.4 Histopathological Examination and Immunohistochemical Staining

After euthanasia, heart samples were photographed under a stereomicroscope and weighed using a digital balance. Heart weight was normalized to the tibial length (TL). The hearts were subsequently fixed in 4% paraformaldehyde (PFA) for 24 h, dehydrated in a graded ethanol series, embedded in paraffin, and sectioned into 4‐µm‐thick paraffin sections. Myocardial fibrosis and collagen content were evaluated using Masson's trichrome staining, and cell surface area was assessed using wheat germ agglutinin (WGA) staining. For immunohistochemical (IHC) staining, sections were blocked with 3% H_2_O_2_, followed by antigen retrieval using 1 m sodium citrate buffer and incubation with primary antibodies overnight at 4°C. After washing with Tris‐buffered saline containing Tween 20, the sections were incubated with horseradish peroxidase (HRP)‐conjugated anti‐mouse/Rabbit IgG for 1 h at room temperature (RT) and stained with 3,3′‐diaminobenzidine solution. The degree of cardiac fibrosis, cell surface area, and 4‐HNE expression were quantified using the ImageJ software (version 1.53c, NIH, Bethesda, MD, USA). The following primary antibodies were used:

**Table:** 

Name	Manufacturer	Cat no.	Host	Reacts with	Dilution
4‐HNE	Abcam	ab48506	Mouse	Human, Mouse	1:50
TUFM	Proteintech	26730‐1‐AP	Rabbit	Human, Mouse	1:400
TrxR2	Thermo Fisher	16360‐1‐AP	Rabbit	Human, Mouse	1:100
Pan‐lactylation	PTMBIO	PTM1401RM	Rabbit	Human, Mouse	1:200

### 2.5 Lectin Perfusion Assay, Dextrans Leakage Assay, and Immunofluorescence Staining

Cardiac microvascular perfusion was assessed using our previously established protocol [7, 46]. Briefly, 100 µL of DyLight594‐labeled tomato lectin (1 mg/mL; Vector Laboratories, USA) was intravenously injected into the tail vein. Ten minutes after injection, heart samples were harvested and processed to prepare frozen sections. Mice were intravenously injected with 100 µL of TRITC‐dextran (50 mg/mL, 65–85 kDa; Sigma‐Aldrich) to evaluate cardiac microvascular permeability [47]. Ten minutes after injection, heart samples were harvested and processed to prepare frozen sections.

Cardiac samples were washed with cold phosphate‐buffered saline (PBS), embedded in optimal cutting temperature compound (Sakura, USA), snap‐frozen in liquid nitrogen, and sectioned into 6‐µm‐thick frozen sections. The frozen sections were fixed with cold acetone for 10 min at −20°C, air‐dried for 15 min, and blocked with 5% bovine serum albumin (BSA) for 1 h at RT. The sections were then incubated with the anti‐CD31 (Ab7388; Abcam, USA), anti‐VCAM‐1 (30958‐1‐AP; Proteintech, China), and anti‐VE‐Cadherin (14‐1441‐82; Thermo Fisher Scientific, Waltham, MA, USA) antibodies at the recommended dilutions. After overnight incubation at 4°C, the samples were washed with PBS and incubated with fluorescence‐labeled secondary antibodies for 1 h at RT. Samples were imaged using a confocal microscope (Olympus FV3000; Japan). At least five random fields were captured and quantitatively analyzed using ImageJ software (version 1.53c; NIH).

### 2.6 Pharmacologic Relaxation of Aortic Rings

After deep anesthesia, the mice were perfused with 10 mL of heparinized (10 IU/mL) normal Krebs–Ringer solution. The distal part of the thoracic aorta was subsequently isolated, and 3‐mm‐long aortic rings were prepared and placed into a vessel chamber filled with equilibrated Krebs–Ringer solution (95% O_2_ balanced with 5% CO_2_) at 37°C for 45 min. The rings were mounted on the stainless‐steel vessel holders (200 µm) of the wire myograph system (DMT620M; Danish Myo Technology, Denmark) and stimulated with 60 mm KCl for 20 min to elicit a standard contraction. Precontraction of the aortic rings was induced with norepinephrine (5 × 10^−^^8^ m), and endothelial relaxation was assessed using acetylcholine (ACh) at concentrations ranging from 10^−^^8^ to 10^−^^5^ m. Relaxant responses are expressed as a percentage of the pre‐contractions induced by norepinephrine.

### 2.7 Cell Isolation, Culture, and Treatment

Human cardiac microvascular ECs (HCMECs) were purchased from Lonza (Basel, Switzerland). Human umbilical vein ECs (HUVECs, Cat: PRI‐H‐00023, RRID: CVCL_2959), human coronary artery ECs (HCAECs, Cat: PRI‐H‐00026), human aortic ECs (HAECs, Cat: PRI‐H‐00021, RRID: CVCL_C0EQ), mouse aortic ECs (MAECs, Cat: PRI‐MOU‐00018, RRID: CVCL_U411), and rat aortic ECs (RAECs, Cat: PRI‐RAT‐00018) were purchased from ZQXZBIO (Shanghai, China). These cell lines were free of mycoplasma contamination, as confirmed by the manufacturer. Mouse cardiac microvascular ECs (MCMECs) and rat cardiac microvascular ECs were isolated from the left ventricular tissue as described previously [46, 48]. Briefly, the endocardium, epicardium, and coronary arteries were excised. Next, the left ventricle was minced into 1‐mm^3^ pieces and thoroughly digested into a single‐cell suspension using Liberase (Roche, Switzerland). The cell suspension was subsequently filtered through a 70‐µm strainer to remove tissue debris and incubated with CD31‐conjugated MicroBeads (Thermo Fisher Scientific) for 15 min at 4°C. The cells were collected using a magnetic separator (Biolinkedin, China). ECs were cultured in fibronectin‐coated dishes with complete EC medium (ScienCell Research Laboratories, USA) between passages 3 and 6.

Primary adult mouse cardiomyocytes were isolated from the ventricles of mice as previously described [49]. Briefly, the mice were euthanized before the heart was immediately perfused with EDTA buffer via the right ventricle to flush out blood. The heart was then transferred to a 60‐mm dish and further digested through the sequential injection of EDTA, perfusion, and collagenase buffers into the left ventricle. After complete digestion, the single‐cell suspension was filtered through a 100‐µm filter. It was subsequently subjected to four sequential rounds of gravity settling using three intermediate calcium reintroduction buffers to gradually restore the calcium concentration. The cells were then plated on laminin‐coated dishes and cultured under standard conditions.

To induce diabetic injury, cells were exposed to high glucose medium (HG; 25 mm glucose) supplemented with 300 µm BSA‐conjugated palmitic acid (PA) for 1 or 3 days, depending on the experimental design [14]. The control cells were treated with 5.5 mm glucose, 19.5 mm mannitol, and 1% BSA. To induce ferroptosis, the cells were treated with RSL3 (MCE, USA) at the indicated concentrations for 4–6 h. Cells were exposed to Alda‐1 (10 µm) for 24 h to enhance ALDH2 activity. To inhibit or stimulate TrxR2 activity, MitoCur‐1 (10 µm, MCE), or Kukoamine B (10 µm, MCE) was applied for the indicated durations. The SCP2 inducer 4‐hydroxytamoxifen (4OHT, 1 µM; Macklin, China) or the activity inhibitor ScpI2 (5 µm; Vitas‐M, Netherlands) was used to regulate SCP2 activity [50].

### 2.8 Genome Editing and Transfection

TrxR1, TrxR2, SCP2, ACSL4, FUNDC1, Parkin, TUFM, Clpp, and AARS2 knockout cell lines, as well as FUNDC1 S17A and TrxR2 K340R mutation cell lines, were constructed by QEgene using the CRISPR–Cas9 system. Single guide RNAs (sgRNAs) were designed using an online CRISPR design tool (http://crispr.mit.edu). Single‐stranded oligodeoxynucleotide (ssODN) templates for generating FUNDC1 S17A and TrxR2 K340R mutations were designed based on previous studies [51]. HCMECs (1 × 10^5^ cells) in a 24‐well plate were infected with 20 multiplicities of infection (MOI) of the lentivirus (LV) packaging lentiCas9‐Blast for 24 h. Stably infected cells were selected using Blasticidin (10 µg/mL) for 72 h. Cells were then transfected with 0.2 µg of the sgRNA plasmid with or without 0.25 µg ssODNs using Lipofectamine 3000 reagents (Invitrogen, Waltham, MA, USA) for 6–8 h. Subsequently, the infected cells were selected with 3 µg/mL puromycin, trypsinized, and diluted to obtain single cells. The single cells were then seeded into 96‐well plates for genotyping validation, which was carried out through sequencing of the PCR products. The sgRNA sequences and ssODNs were as follows:

**Table:** 

Cell lines	sgRNA
TrxR1 KO	TCAAGTTCAAGCACAAAATA
TrxR2 KO	TCACGCCCATTATAAACCAC
TrxR2 K340R	CCCCGACACTCAGAAGATCC
SCP2 KO	GGTTGTAACACTCTACAAGA
ACSL4 KO	AATCGCAGAGTGAATAACTT
FUNDC1 KO	CAAACACTCGATTCCACCAC
FUNDC1 S17A	TGACGACTCTTATGAAGTGT
Parkin KO	GTCAGGTTCAACTCCAGCCA
TUFM KO	CAGTCAGCGTGGTCTTCCCG
Clpp KO	GCGCCTATGACATCTACTCG
AARS2 KO	TTCGAAGGTCGCCCGCATGG
	ssoDN
FUNDC1 S17A	GCATTTTGCTATTTTGAAGACTATGAAAGTGATGACGACGCTTATGAAGTGTTGGATTTAACTGAGTATGCAAGAAGACACCAGTGGTGGAATCGAGTGTTTGGCCACAGTTCGGGACCTATGGTAG
TrxR2 K340R	TTGGAGAAGGCTGGGGTAGATACTAGCCCCGACACTCAGAGGATCCTGGTGGACTCCCGGGAAGCCACCTCTGTGCCCCAC

HCMECs were transfected with small interfering RNAs (Tylinphire Biotech, China) using Lipofectamine 3000 reagents (Invitrogen) to knock down target genes. LVs encoding TrxR2, aAMPKα1, and aMitoAα1 were designed and constructed by QEgene for stable transfection. The cells were infected with adenoviruses encoding mt‐Keima (Hanbio Biotechnology Co. Ltd., China) at the recommended MOI to detect mitophagy. Chaperone‐mediated autophagy (CMA) of GPX4 and SCP2 was detected as described previously [52, 53]. The plasmids expressing PAmCherry‐GPX4‐NE and PAmCherry‐SCP2‐NE were provided by QEgene. After transfection and treatment, immunofluorescence was performed using a monoclonal anti‐NE antibody. Similarly, a plasmid expressing mitoABKAR (QEgene) was used to detect mitochondrial AMPK activity via Förster resonance energy transfer, as previously described [54]. For lactyltransferase screening, plasmids encoding HA‐tagged KAT5 and NAT13 and Myc‐tagged P300, CREBBP, AARS1, and AARS2 were generated for co‐immunoprecipitation assays.

### 2.9 Cell Viability Assay

Cell viability was assessed using a Cell Counting Kit‐8 (CCK‐8; EpiZyme, China). Cells were seeded in 96‐well plates, and 100 µL of CCK‐8 working solution was added to each well. After incubation at 37°C for 2 h, absorbance was measured at 450 nm using a microplate reader (Varioskan LUX; Thermo Fisher Scientific).

### 2.10 Evaluation of mitoTrxR Activity

mitoTrxR activity was measured as previously described [55, 56]. Cells were incubated with 10 µm Mito‐TRFS (MCE) at 37°C for 20 min in the dark. Mito‐TRFS fluorescence was excited at 375 nm with an emission wavelength of 480 nm using a confocal microscope (Olympus FV3000; Olympus, Japan) and a microplate reader (Varioskan LUX; Thermo Fisher Scientific). Fluorescence intensity was normalized to the cell number. To inhibit mitoTrxR activity, cells were treated with TrxR1 inhibitors (TrxR1‐IN‐1 and TrxR‐IN‐B19), a TrxR2 inhibitor (mitoCur‐1), and TrxRs inhibitors (TrxR‐IN‐2 and TrxR‐IN‐5) for 6 h at the indicated concentrations.

### 2.11 Mitochondrial Fluorescence Staining

Mitochondrial morphology was visualized through staining with 0.5 µm MitoTracker Deep Red FM (Thermo Fisher Scientific) for 20 min at 37°C in the dark. The mitochondrial ROS (mitoROS) levels and mitochondrial membrane potential (MMP) were assessed through staining with MitoSOX Red Mitochondrial Superoxide Indicator (5 µm; Invitrogen) for 30 min and TMRM (100 nm; Invitrogen) for 20 min in the dark, respectively. Samples were observed and imaged using a confocal microscope (Olympus FV3000; Olympus), and fluorescence intensity was analyzed using ImageJ (version 1.53c; NIH).

### 2.12 Ferroptosis Assessment

The cells were seeded in confocal dishes (NEST, China) or 96‐well black plates (Corning Inc., Corning, NY, USA). They were then incubated with 5 µm Mito‐FerroGreen (Dojindo Laboratories, Japan) and 1 µm FerroOrange (Dojindo Laboratories) at 37°C for 30 min to detect mitochondrial ferrous iron ([Fe^2+^]_m_) and intracellular ferrous iron ([Fe^2+^]_i_). Next, the cells were incubated with 5 µm BODIPY 581/591 C11 (MCE) and 5 µM MitoPerOx (MCE) for 30 min at 37°C in the dark to assess intracellular lipid peroxidation (LPO) and mitochondrial LPO (mitoLPO), respectively [57]. Cell death was evaluated through staining with 5 µg/mL Hoechst 33342 and 10 µg/mL propidium iodide (PI; Beyotime, China) for 20 min in the dark. Confocal dishes were observed and imaged using a confocal microscope (Olympus FV3000; Olympus), and fluorescence intensity was analyzed using ImageJ (version 1.53c; NIH). Cell death was quantified as the percentage of PI‐positive cells relative to Hoechst 33342‐stained cells. The fluorescence intensities in 96‐well black plates were measured using a microplate reader (Varioskan LUX; Thermo Fisher Scientific) at the corresponding excitation and emission wavelengths. Plasma malondialdehyde (MDA) levels were determined using a lipid peroxidation MDA assay kit (Beyotime) according to the manufacturer's instructions.

### 2.13 Immunofluorescence Staining

Cells were seeded in confocal dishes, fixed with 4% PFA, permeabilized with 0.1% Triton X‐100, and blocked with 5% BSA. After incubation with primary antibodies overnight at 4°C and fluorescence‐labeled secondary antibodies for 1 h, the dishes were observed using a confocal microscope (Olympus FV3000). Immunofluorescence images were quantitatively analyzed using ImageJ (version 1.53c; NIH). Protein colocalizations, including that of ACSL4, Tomm20, SCP2, and Flag‐tagged proteins, were analyzed using the Colocalization plugin in ImageJ. The following major primary antibodies were used:

**Table:** 

Name	Manufacturer	Cat no.	Host	Reacts with	Dilution
SCP2	Proteintech	23006‐1‐AP	Rabbit	Human, Mouse	1:100
Tomm20	Abcam	ab186735	Rabbit	Human, Mouse	1:200
Tomm20	Abcam	ab56783	Mouse	Human, Mouse	1:200
ACSL4	Abcam	ab155282	Rabbit	Human, Mouse	1:100
ACSL4	Thermo Fisher	MA5‐31548	Mouse	Human, Mouse	1:200
Flag tag	Abcam	ab205606	Rabbit	Human, Mouse	1:100
Flag tag	Abcam	Ab18230	Mouse	Human, Mouse	1:100
COX IV	Abcam	ab33985	Mouse	Human, Mouse	1:500
GPX4	Abcam	ab125066	Rabbit	Human, Mouse	1:200
LAMP2a	Abcam	ab25631	Mouse	Human, Mouse	1:100
FTH1	Abcam	Ab75973	Rabbit	Human, Mouse	1:200
NCOA4	NOVUS	H00008031‐M04	Mouse	Human, Mouse	1:200
LAMP1	CST	15665S	Mouse	Human	1:250

### 2.14 In Situ Proximity Ligation Assay (PLA)

PLA was performed using the Duolink In Situ Red Starter Kit Mouse/Rabbit assay kit (Sigma‐Aldrich) as described previously [58]. Cells were fixed with 4% PFA for 20 min at RT, permeabilized with 0.1% Triton X‐100 for 5 min, and blocked with PLA blocking buffer for 1 h at 37°C. The cells were subsequently incubated overnight at 4°C with the following primary antibodies: rabbit anti‐SCP2 (1:100; Proteintech, 23006‐1‐AP), rabbit anti‐AARS2 (1:50; Bioss, bs‐1603R), mouse anti‐ACSL4 (1:200, Thermo Fisher Scientific, MA5‐31548), and mouse anti‐TrxR2 (1:100; Santa Cruz Biotechnology, sc‐166259). They were then incubated with the PLA probes (Anti‐Rabbit PLUS and Anti‐Mouse MINUS) for 1 h at 37°C, followed by ligation and amplification. Samples were observed under a confocal microscope (Olympus FV3000; Japan), and fluorescent signals were quantified using ImageJ (version 1.53c; NIH).

### 2.15 Mitochondrial Oxygen Consumption Rate (OCR)

Mitochondrial OCR levels were measured using a Seahorse XF96 Extracellular Flux Analyzer (Seahorse Bioscience, USA) as previously reported [47]. HCMECs (2 × 10^4^ cells/well) were seeded onto XF96 cell culture plates and incubated in Seahorse XF medium (pH 7.4). Oligomycin, FCCP, rotenone, and antimycin A were sequentially injected according to the manufacturer's instructions. Basal, ATP‐linked, maximal, and spare respiratory OCR were normalized to the cell number per well by counting cells upon completion of the XF assay.

### 2.16 Lipid Extraction and Mass Spectrometry (MS)

Samples were homogenized with 200 µL of water and 240 µL of methanol. The mixture was combined with 800 µL of methyl tert‐butyl ether and subjected to ultrasonication for 20 min at 4°C. After incubation for 30 min at RT, the mixture was centrifuged at 14,000 × *g* for 15 min at 10°C. The upper layers were collected and dried under a stream of nitrogen. Subsequently, the lipid extracts were analyzed using MS and data analysis was performed by Applied Protein Technology Co., Ltd. (APTBIO, Shanghai, China) [59].

### 2.17 ELISA and ALDH2 Activity

The 5‐HETE, 12‐HETE, 15‐HETE, and 4‐HNE levels in cell lysates were measured using the following commercial ELISA kits: 5‐HETE (CUSABIO, China), 12‐HETE (Abcam), 15‐HETE (Abcam), and 4‐HNE (CUSABIO) ELISA kits according to the manufacturers’ protocols. ALDH2 activity was quantified using an ALDH2 Activity Assay Kit (Abcam) according to the manufacturer's instructions.

### 2.18 Co‐Immunoprecipitation (Co‐IP) Assay, IP‐MS Assay, and Western Blotting

Antibodies against ACSL4 (Abcam; ab155282), TrxR2 (Santa Cruz Biotechnology; sc‐166259), SCP2 (Proteintech; 23006‐1‐AP), Flag tag (Abcam; ab205606), HA tag (Abcam; ab9110), GPX4 (Proteintech; 67763‐1‐Ig), NCOA4 (Abcam; ab314553), FUNDC1 (CST; 49240S), TUFM (Abcam; ab173300), and AMPKα1 (Abcam, ab32047) were pre‐incubated with magnetic beads (Biolinkedin, China) at 4°C for 2 h. Next, cell samples were lysed using a Cell Lysis Buffer for IP (Beyotime) and incubated with the prepared magnetic beads at 4°C overnight. After extensive washing, the immunocomplex samples were boiled in a loading buffer for 10 min to elute the target proteins. For the IP‐MS assay, the samples were separated on 10% Tris‐glycine gels and stained with Coomassie brilliant blue. Specific bands were cut and digested with trypsin for MS analysis at Applied Protein Technology Co., Ltd. (Shanghai, China).

Mitochondrial and lysosomal samples were isolated using a Mitochondrial Separation Reagent (Beyotime) and a Lysosome Protein Extraction Kit (Bjbalb, China), respectively, according to the manufacturers’ instructions. Lysates (10–20 µg protein per lane) and Co‐IP samples were separated by SDS‐PAGE using 7.5%, 10%, or 12.5% gels and subsequently transferred to polyvinylidene difluoride membranes (Millipore, USA). The membranes were blocked with blocking buffer (Epizyme) for 20 min at RT. After blocking, the membranes were incubated with primary antibodies at the recommended dilutions overnight at 4°C, followed by probing with HRP‐labeled goat anti‐rabbit or mouse IgG (H+L) for 1 h at RT. Protein bands were visualized using an enhanced chemiluminescence reagent (Millipore) according to the manufacturer's instructions.

Redox western blotting was performed as described previously [60]. The mitochondrial pellet was washed with acetone (–20°C) and centrifuged at 21 000 × *g* for 10 min. Acetone was removed, and the pellet was solubilized in labeling buffer (670 mm Tris‐HCl (pH 7.5), 2% SDS, 1 mm EDTA, and 10 mm AMS (Invitrogen) and incubated at 37°C for 1 h. Samples were precipitated and washed with acetone, followed by molecular weight separation through 15% polyacrylamide gel electrophoresis with non‐reducing loading buffer.

The following major primary antibodies were used:

**Table:** 

Name	Manufacturer	Cat no.	Host	Reacts with	Dilution
Trx2	Proteintech	20886‐1‐AP	Rabbit	Human, Mouse	1:3000
Prdx3	Proteintech	81833‐1‐RR	Rabbit	Human, Mouse	1:5000
TrxR2	Thermo Fisher	PA5‐96344	Rabbit	Human, Mouse	1:1000
TrxR1	Abcam	ab124954	Rabbit	Human, Mouse	1:2000
ACSL4	Abcam	ab155282	Rabbit	Human, Mouse	1:3000
GPX4	Abcam	ab125066	Rabbit	Human, Mouse	1:1000
ACOT2	Proteintech	15633‐1‐AP	Rabbit	Human, Mouse	1:500
StAR	Proteintech	80751‐1‐RR	Rabbit	Human, Mouse	1:2000
ALOX12	Abcam	Ab168384	Rabbit	Human	1:4000
4‐HNE	Abcam	ab48506	Mouse	Human, Mouse	1:1000
SCP2	Proteintech	23006‐1‐AP	Rabbit	Human, Mouse	1:2000
VDAC2	Abcam	ab316107	Rabbit	Human, Mouse	1:1000
Tomm20	Abcam	ab186735	Rabbit	Human, Mouse	1:5000
Flag tag	Abcam	ab205606	Rabbit	Human, Mouse	1:500
HA tag	Abcam	ab9110	Rabbit	Human, Mouse	1:4000
GST	Abcam	ab252882	Rat	Human, Mouse	1:1000
**p‐FUNDC1 ^Ser17^**	Affinity	AF0001	Rabbit	Human	1:2000
FUNDC1	CST	49240S	Rabbit	Human, Mouse	1:2000
Pink1	Abcam	ab300623	Rabbit	Human	1:1000
Parkin	Proteintech	14060‐1‐AP	Rabbit	Human, Mouse	1:2000
LAMP2a	Abcam	ab25631	Mouse	Human, Mouse	1:1000
HSP90	Abcam	ab59459	Mouse	Human, Mouse	1:1000
HSC70	Abcam	ab51052	Rabbit	Human, Mouse	1:500
FTH1	Abcam	ab75973	Rabbit	Human, Mouse	1:2000
FTL	ABclonal	A11241	Rabbit	Human, Mouse	1:5000
NCOA4	NOVUS	H00008031‐M04	Mouse	Human, Mouse	1:2000
LAMP1	CST	15665S	Mouse	Human	1:2000
ALDH2	CST	18818S	Rabbit	Human	1:500
p‐AMPK	Abcam	ab133448	Rabbit	Human, Mouse	1:4000
AMPK	Abcam	ab131512	Rabbit	Human, Mouse	1:1000
TUFM	Thermo Fisher	MA5‐31363	Mouse	Human	1:1000
Clpp	Affinity	DF8448	Rabbit	Human, Mouse	1:1000
AARS2	Proteintech	22696‐1‐AP	Rabbit	Human	1:2000
**Pan‐lactylation**	PTMBIO	PTM1401RM	Rabbit	Human, Mouse	1:500
COX IV	CST	4850S	Rabbit	Human	1:1000
ACTB	Abcam	ab8227	Rabbit	Mouse, Human	1:3000

### 2.19 Quantitative Real‐Time PCR (qPCR)

Total RNA was extracted using a commercial kit (Cat No. 74104; QIAGEN) according to the manufacturer's instructions. mRNA was reverse transcribed into complementary DNA using the PrimeScript RT Reagent Kit (Cat No. RR036A; Takara Bio). The purity of total RNA was evaluated based on the absorbance ratio at 260 and 280 nm. qPCR was performed using the SYBR Premix Ex Taq II (Cat No. RR820A; Takara Bio) on a CFX96 Real‐Time System (Bio‐Rad). The thermocycling conditions for qPCR amplification were as follows: denaturation at 95°C for 10 s, followed by annealing and extension at 60°C for 30 s, for a total of 39 cycles. The primer sequences used were as follows:

**Table:** 

Human	Forward	Reverse
Drp1	TCACCCGGAGACCTCTCATTC	GGTTCAGGGCTTACTCCCTTAT
Fis1	AGCGGGATTACGTCTTCTACC	CATGCCCACGAGTCCATCTTT
Mief1	CACGGCCATTGACTTTGTGC	TCGTACATCCGCTTAACTGCC
Mief2	ATGGCAGAGTTCTCCCAGAAA	CCCTGTCAATGAACCGCTT
Mff	CACCACCTCGTGTACTTACGC	GTCTGCCAACTGCTCGGATTT
Mfn1	ATGACCTGGTGTTAGTAGACAGT	AGACATCAGCATCTAGGCAAAAC
Mfn2	GGCCCAACTCTAAGTGCCC	AAGTGCTTTTCCGTCTGCATC
Opa1	CGACCCCAATTAAGGACATCC	GCGAGGCTGGTAGCCATATTT
Mouse		
TrxR1	GGGTCCTATGACTTCGACCTG	AGTCGGTGTGACAAAATCCAAG
TrxR2	GATCCGGTGGCCTAGCTTG	TCGGGGAGAAGGTTCCACAT
Fundc1	AGCGATGACGAATCATACGAAG	CCACCCATTACAATCTGAGTAGC
Slc2a1	GCAGTTCGGCTATAACACTGG	GCGGTGGTTCCATGTTTGATTG
Hk2	ATGATCGCCTGCTTATTCACG	CGCCTAGAAATCTCCAGAAGGG
Pfkp	CGCCTATCCGAAGTACCTGGA	CCCCGTGTAGATTCCCATGC
Gapdh	TGACCTCAACTACATGGTCTACA	CTTCCCATTCTCGGCCTTG
Pgk1	ATGTCGCTTTCCAACAAGCTG	GCTCCATTGTCCAAGCAGAAT
Pgam1	AGCGACACTATGGCGGTCT	TGGGACATCATAAGATCGTCTCC
Eno1	GTACCGCCACATCGCTGACTTG	AGCATGAGAACCGCCATTGATGAC
Pkm1/2	CGCCTGGACATTGACTCTG	GAAATTCAGCCGAGCCACATT
Ldha	CAAAGACTACTGTGTAACTGCGA	TGGACTGTACTTGACAATGTTGG
Ldhb	TTCCTCCAGACTCCGAAAATTG	GTTTCCAGGTGACGTAAGTCAG

### 2.20 RNA‐Sequencing Assay and Bioinformatic Analysis

RNA‐sequencing was performed by OE Biotech (Shanghai, China). Total RNA was extracted from the cells using TRIzol reagent, and RNA purity and concentration were measured using a NanoDrop 2000 spectrophotometer (Thermo Fisher Scientific). The TruSeq Stranded mRNA LT Sample Prep Kit (Illumina, San Diego, CA, USA) was used to construct the sequencing library. Gene expression levels were quantified as fragments per kilobase of transcript per million mapped reads (FPKM) using RSEM (v1.2.12). Differentially expressed genes (DEGs) were identified using the R package “EdgeR,” with a threshold of fold change > 1.5 and false discovery rate < 0.05. Principal component analysis was performed using R (v3.2.0) to assess the biological replicates. Furthermore, differential expression analysis was performed using DESeq2. Hierarchical clustering analysis of DEGs was performed using R (v3.2.0) to visualize gene expression patterns across different groups and samples. Kyoto Encyclopedia of Genes and Genomes (KEGG) pathway enrichment analysis was conducted to identify significantly enriched terms using R (v3.2.0).

### 2.21 TrxR2 Agonist Screening via Molecular Docking

TrxR2 agonists were screened using molecular docking assays as described previously [61]. The protein structure of TrxR2 was predicted using the AlphaFold3 software (https://alphafoldserver. com/). In total, 79044 molecules from the MCE Virtual Screening Database (HY‐L001P) were docked with TrxR2 using the LibDock module of the Discovery Studio software (version3.5). Flexible docking of 15730 selected molecules (score > 120) to each potential binding site on TrxR2 was performed using the CDOCKER module; 115 matching compounds were obtained (score > 25). Finally, six molecules—Kukoamine B, Olumacostat glasaretil (DRM01), E6446, KY‐226, Otaplimastat, and AUTAC4—were selected as potential TrxR2 agonists based on binding pattern analysis and the manufacturer's instructions.

### 2.22 Statistical Analysis

Data are presented as the mean ± standard error of the mean (SEM). Normality was assessed using the Shapiro–Wilk and Kolmogorov–Smirnov tests. Statistical analyses were conducted using unpaired two‐tailed Student's t‐test or one‐way analysis of variance (ANOVA), followed by Tukey's post hoc test. For comparisons involving two independent variables, a two‐way ANOVA with Tukey's post‐hoc test was applied. The Mann‐Whitney U test was used for non‐normally distributed data between two groups. Simple linear regression analysis was performed to assess the correlation between 4‐HNE and TUFM. Moreover, statistical significance was defined as a two‐sided *p*‐value < 0.05. Data analysis and visualization were performed using GraphPad Prism (version 10.0; GraphPad, USA). The detailed statistical methodology and sample sizes for each analysis are provided in the figure legends.

## 3 Results

### 3.1 TrxR2 Contributed to Endothelial mitoTrxR Activity and Mitochondrial Protection

**FIGURE 1:**
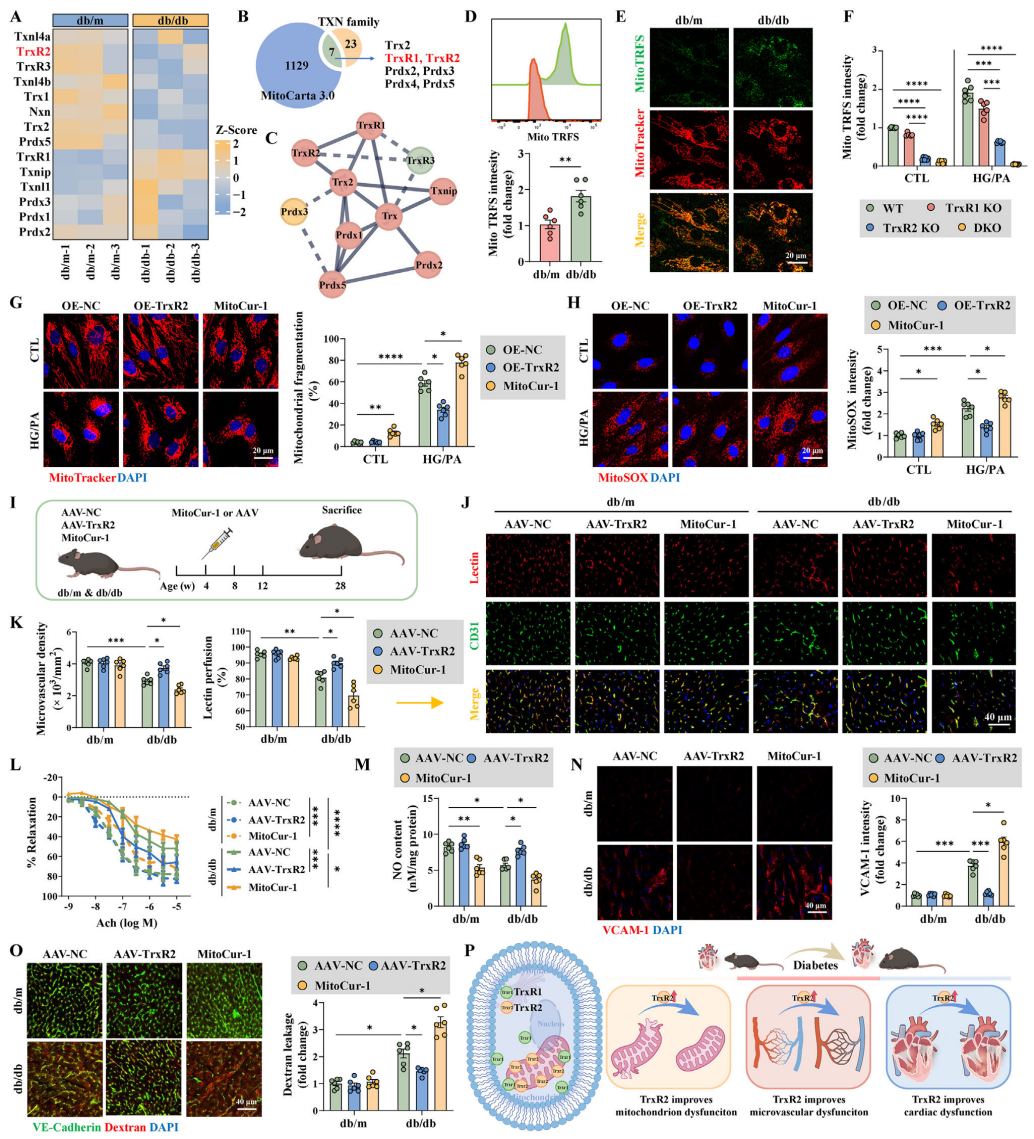
TrxR2 improved mitochondrial dysfunction and cardiac microvascular injury in diabetic cardiomyopathy. (A) A heatmap illustrating the expression of thioredoxin family members, as determined through RNA‐sequencing assay in db/m and db/db mice. (B) A Venn diagram shows seven mitochondria‐related genes within the thioredoxin family, as determined utilizing the MitoCarta 3.0 database. (C) Interactions among thioredoxin family members were generated from the STRING database. (D,E) MitoTRFS intensity in primary MCMECs isolated from db/m and db/db mice, as detected using flow cytometry (D) and confocal microscopy (E) (*n* = 6 per group). (F) Combined or individual TrxR1 and TrxR2 knockout in HCMECs; MitoTRFS intensity was measured and statistically analyzed following CTL and HG/PA treatments (*n* = 6 per group). (G,H) HCMECs were overexpressed with TrxR2 or treated with the TrxR2 inhibitor (MitoCur‐1). Mitochondrial fragmentation and mitoROS levels were measured by MitoTracker (G) and MitoSOX (H) fluorescence staining, respectively (*n* = 6 per group). (I) Schematic outline of AAV‐TrxR2 transfection and MitoCur‐1 treatment of db/m and db/db mice. MitoCur‐1 (10 mg/kg/day) was intraperitoneally administered for 24 weeks, whereas AAV‐TrxR2 was intravenously administered every eight weeks during the 24‐week period. (J,K) Representative immunofluorescence images and quantification of CD31‐labeled cardiac microcirculation and lectin‐perfused vessels (*n* = 6 per group). (L) Endothelium‐dependent vasodilation, as assessed using wire myography to measure aortic ring tension responses to Ach solution at specified concentrations (*n* = 3 per group). (M) The NO content in heart samples was statistically analyzed (*n* = 6 per group). (N) Representative immunofluorescence images and quantification of VCAM‐1 expression (*n* = 6 per group). (O) Representative immunofluorescence images and quantification of VE‐Cadherin expression and TRITC‐dextran leakage (*n* = 6 per group). (P) A diagram illustrating that TrxR2 overexpression improves mitochondrial function, microvascular function, and cardiac function in diabetic mice. Unpaired Student's t‐test was used for D. Two‐way analysis of variance (ANOVA) followed by Tukey's post‐hoc multi‐comparison test was used for F‐O. *p < 0.05, **p < 0.01, ***p < 0.001, ****p < 0.0001 indicate statistically significant differences. AAV, adeno‐associated virus; ACh, acetylcholine; CTL, control; HG/PA, high glucose and palmitic acid treatment; KO, knockout; OE, overexpression; TXN, thioredoxin; WT, wild‐type.

RNA‐sequencing assay was performed to determine the role of the thioredoxin family in DCM. Members of the thioredoxin family were significantly dysregulated in diabetic db/db hearts compared with those in non‐diabetic db/m mice (Figure 1A). Gene ontology (GO) analyses revealed that these members were enriched in cell redox homeostasis and oxidant detoxification, peroxiredoxin activity, inhibition of apoptosis, and mitochondrial localization (Figure S1A). Using MitoCarta 3.0 database, we identified seven mitochondria‐localized members, including TrxR1 and TrxR2, two of the core members of this family (Figure 1B,C). Both TrxR1 and TrxR2 were highly expressed in sorted primary ECs of db/m and db/db mice (Figure S1B). In addition, inhibitors targeting TrxR1 (TrxR1‐IN‐1 and TrxR‐IN‐B19), TrxR2 (mitoCur‐1), and TrxRs (TrxR‐IN‐2 and TrxR‐IN‐5) revealed that both TrxR1 and TrxR2 contribute to endothelial mitoTrxR activity (Figure S1C). Considering endothelial mitoTrxR activity increased in primary MCMECs sorted from db/db mice, TrxR1 and TrxR2 were knocked out using the CRISPR‐Cas9 system to determine which plays the dominant role (Figure 1D–F; Figure S1D). mitoTrxR activity in HCMECs increased under HG/PA injury, the effects of which were inhibited by TrxR2 KO and TrxR1 KO; but the effect was more obvious following TrxR2 KO (Figure 1F). In addition, TrxR2 reduced mitochondrial oxidative stress, preserved the MMP, balanced mitochondrial dynamics, and improved mitochondrial respiration when overexpressed in HCMECs during HG/PA injury (Figure 1G,H; Figure S1E–H). However, suppression of mitoTrxR activity via the TrxR2 inhibitor MitoCur‐1 accentuated mitochondrial dysfunction (Figure 1G,H; Figure S1E–H). These findings confirmed that TrxR2 is critical for regulating mitoTrxR activity and maintaining mitochondrial integrity in the context of diabetic endothelial injury.

### 3.2 TrxR2 Protected Cardiac Microcirculation Against Long‐Term Diabetes

Having established the protective effects of TrxR2 on mitochondria and ECs, we subsequently investigated its role in cardiac microvascular protection in DCM. To this end, endothelium‐specific overexpression of TrxR2 was achieved via AAV9 transfection (Figure 1I; Figure S2A,B). db/db mice exhibited reduced cardiac microvascular density and blood perfusion after 24 weeks of long‐term diabetes, primarily because of decreased NO generation and impaired endothelium‐dependent vasodilation (Figure 1J–M). However, these effects were alleviated by TrxR2 overexpression (Figure 1J–M). In addition, TrxR2 overexpression reduced the plasma expression of sICAM‐1 and vWF, and the cardiac expression of VCAM‐1 to suppress endothelium‐dependent inflammation (Figure 1N; Figure S2C). TrxR2 overexpression further alleviated cardiac microvascular leakage by enhancing VE‐Cadherin expression (Figure 1O). Cardiac microvascular protection mediated by endothelium‐specific TrxR2 overexpression improved cardiac pathology and function, as demonstrated by reduced cardiac hypertrophy, decreased fiber deposition, and improved systolic and diastolic dysfunction (Figure S2D–G). Collectively, these data underscore the mitochondrial and cardiovascular protective effects of TrxR2 in DCM (Figure 1P).

The TrxR2 inhibitor MitoCur‐1 was used to further investigate the potential effects of TrxR2 on cardiac protection. Administration of MitoCur‐1 for 24 weeks impaired endothelial‐dependent vascular relaxation in non‐diabetic db/m mice (Figure 1J–M). Furthermore, it exacerbated cardiac microvascular injury and myocardial dysfunction in diabetic db/db mice (Figure 1I–O; Figre S2C–G). In addition, the endothelium‐specific protection of TrxR2 was evaluated in TrxR2^EC‐KO^ mice, avoiding the off‐target effects of MitoCur‐1. TrxR2^EC‐KO^ and littermate TrxR2^flox/flox^ mice were subjected to DCM using the HFD/STZ method (Figure S3A). TrxR2^EC‐KO^ mice exhibited microvascular sparsity, hypoperfusion, cardiomyocyte hypertrophy, and cardiac diastolic dysfunction even in the CTL group (Figure S3B,C,E–G). More importantly, TrxR2 knockout exacerbated cardiac microvascular injury, cardiac pathological remodeling, and cardiac dysfunction in the DCM group (Figure S3B–G).

### 3.3 TrxR2 and Its Agonist Kukoamine B Prevented Ferroptosis by Suppressing Lipid Peroxidation

**FIGURE 2:**
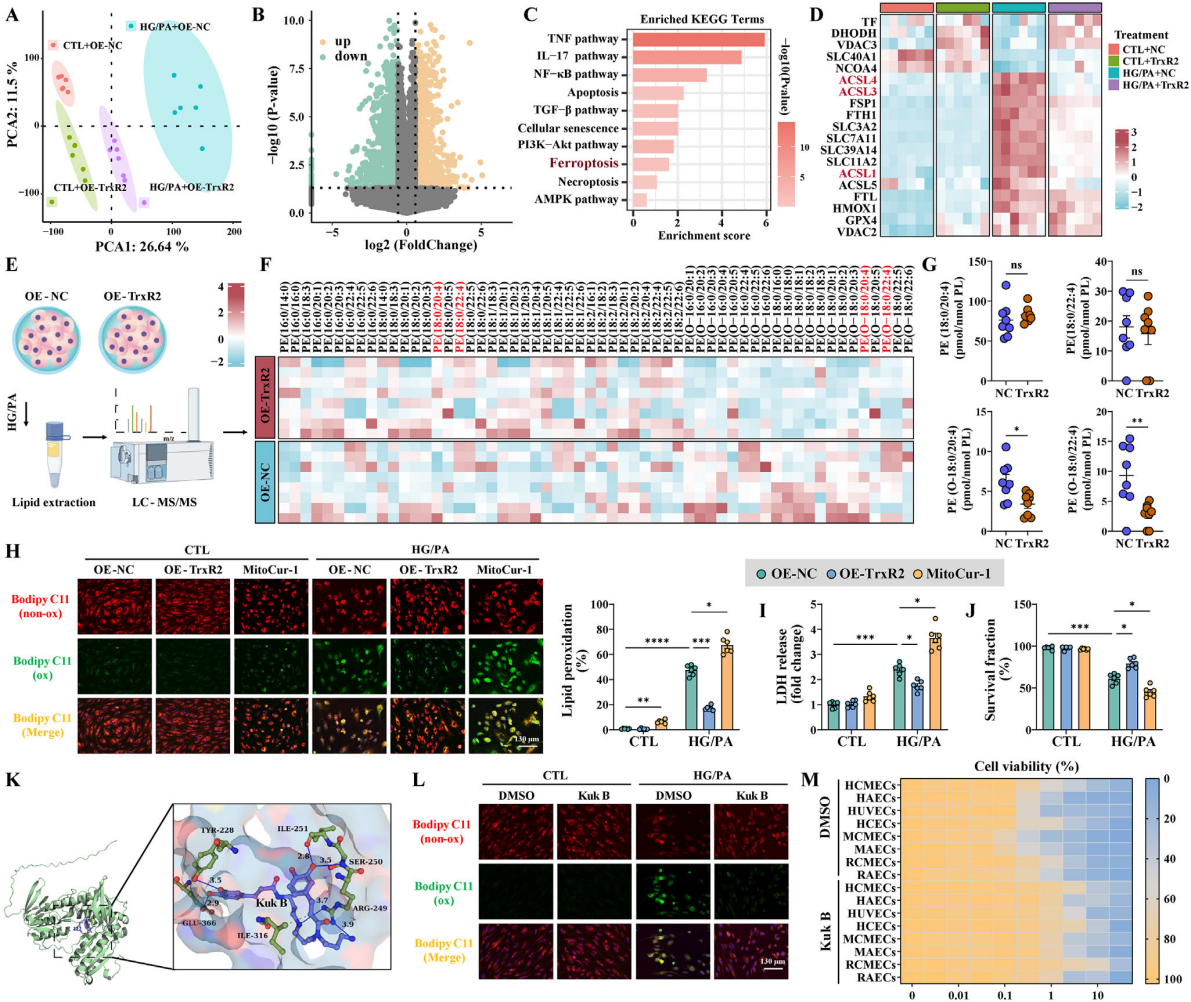
TrxR2 prevented ferroptosis by suppressing the formation of lipid peroxidation. (A) Principal component analysis of indicated groups. (B,C) Volcano plots and KEGG analysis of differentially expressed genes (DEGs), as identified by RNA‐sequencing assay in HCMECs treated with HG/PA, with or without TrxR2 overexpression. (D) A heat map of ferroptosis genes in the indicated groups. (E–G) Lipidomic analysis of HCMECs following HG/PA treatment (E); the levels of all major PE species are summarized in a heatmap (F). PE molecular species (18:0/20:4) and (18:0/22:4) as well as hydroperoxy‐PE molecular species (18:0/20:4) and (18:0/22:4) were quantitatively assessed (G) (*n* = 8 per group). (H) Representative immunofluorescence images and quantification of BODIPY C11 staining in the indicated groups (*n* = 6 per group). (I,J) LDH released into the supernatant and the surviving fraction were quantitatively analyzed (*n* = 6 per group). (K) Molecular docking analysis predicted that Kukoamine B forms six hydrogen bonds with TrxR2. (L) Representative immunofluorescence images of BODIPY C11 staining in HCMECs treated with HG/PA and/or Kuk B (10 µm) for 72 h (*n* = 6 per group). (M) Multiple endothelial cell lines were treated with 10 µm Kukoamine B (Kuk B) for 24 h, followed by exposure to RSL3 at indicated concentrations for 6 h. The relative cell viability is summarized in a heatmap (*n* = 3 per group). Unpaired Student's t‐test was used for G. Two‐way ANOVA, followed by Tukey's post‐hoc multi‐comparison test, was used for H, I, and J. *p < 0.05, **p < 0.01, ***p < 0.001, and ****p < 0.0001 indicate statistically significant differences. Kuk B, Kukoamine B; PCA, principal component analysis; PE, phosphatidylethanolamine.

KEGG enrichment and GSEA analyses were performed using RNA‐sequencing data to further elucidate the mechanisms through which TrxR2 protects ECs from diabetes (Figure 2A,B). Pathways related to ferroptosis, apoptosis, mitophagy, and autophagy were significantly enriched (Figure 2C; Figure S4A). In addition, TrxR2 overexpression suppressed the expression of *ACSL1*, *ACSL3*, and *ACSL4*, which generate LPO (Figure 2D). To further confirm the role of TrxR2 in the modulation of LPO, a lipidomic approach was used as described previously (Figure 2E) [11]. Arachidonic acid (AA)‐ and adrenic acid (AdA)‐containing species in phosphatidylethanolamine (PE) are the preferred substrates for oxidation [11]. In the present study, TrxR2 reduced the levels of oxidized AA‐ and AdA‐containing PE species, specifically PE(O‐18:0/20:4) and PE(O‐18:0/22:4) (Figure 2F,G). LPO formation was further assessed using the BODIPY C11 dye and an LDH ELISA kit. TrxR2 reduced the intensity of oxidized BODIPY C11, decreased LDH release, and improved cell survival in HG/PA‐ and RSL3‐established ferroptosis models (Figure 2H–J; Figure S4B,C). These findings demonstrate that TrxR2 protects ECs from ferroptosis by inhibiting LPO formation.

To further confirm mitoTrxR activity in ferroptosis resistance, we performed molecular docking analysis with TrxR2 and the MCE Virtual Screen Database to identify TrxR2 agonists (Figure 2K). Among the selected Kukoamine B (Kuk B), Olumacostat glasaretil (DRM01), E6446, KY‐226, Otaplimastat, and AUTAC4, Kuk B was identified as the most effective agonist in the improvement of mitoTrxR activity and cell viability under HG/PA injury and RSL3‐established ferroptosis models (Figure S4D,E). Kuk B increased cell viability in a dose‐dependent manner under HG/PA injury, even with MitoCur‐1 treatment (Figure S4F). However, this effect was abolished by TrxR2 KO (Figure S4F). KEGG enrichment analysis revealed that Kuk B treatment, similar to TrxR2 overexpression, suppressed ferroptosis and the expression of genes involved in LPO formation (Figure S4G,H). Additionally, Kuk B downregulated ACSL4 expression, upregulated GPX4 expression, and reduced HG/PA‐induced LPO formation (Figure 2L; Figure S4I). The inhibitory effects of Kuk B on ferroptosis were further validated in multiple EC lines using the RSL3‐ferroptosis model (Figure 2M). These findings demonstrate that TrxR2 and mitoTrxR inhibit ferroptosis by suppressing LPO formation.

### 3.4 TrxR2 Inhibited Ferroptosis by Suppressing Mitochondrial Translocation of ACSL4 and Mitochondrial Eicosanoid Biosynthesis

**FIGURE 3:**
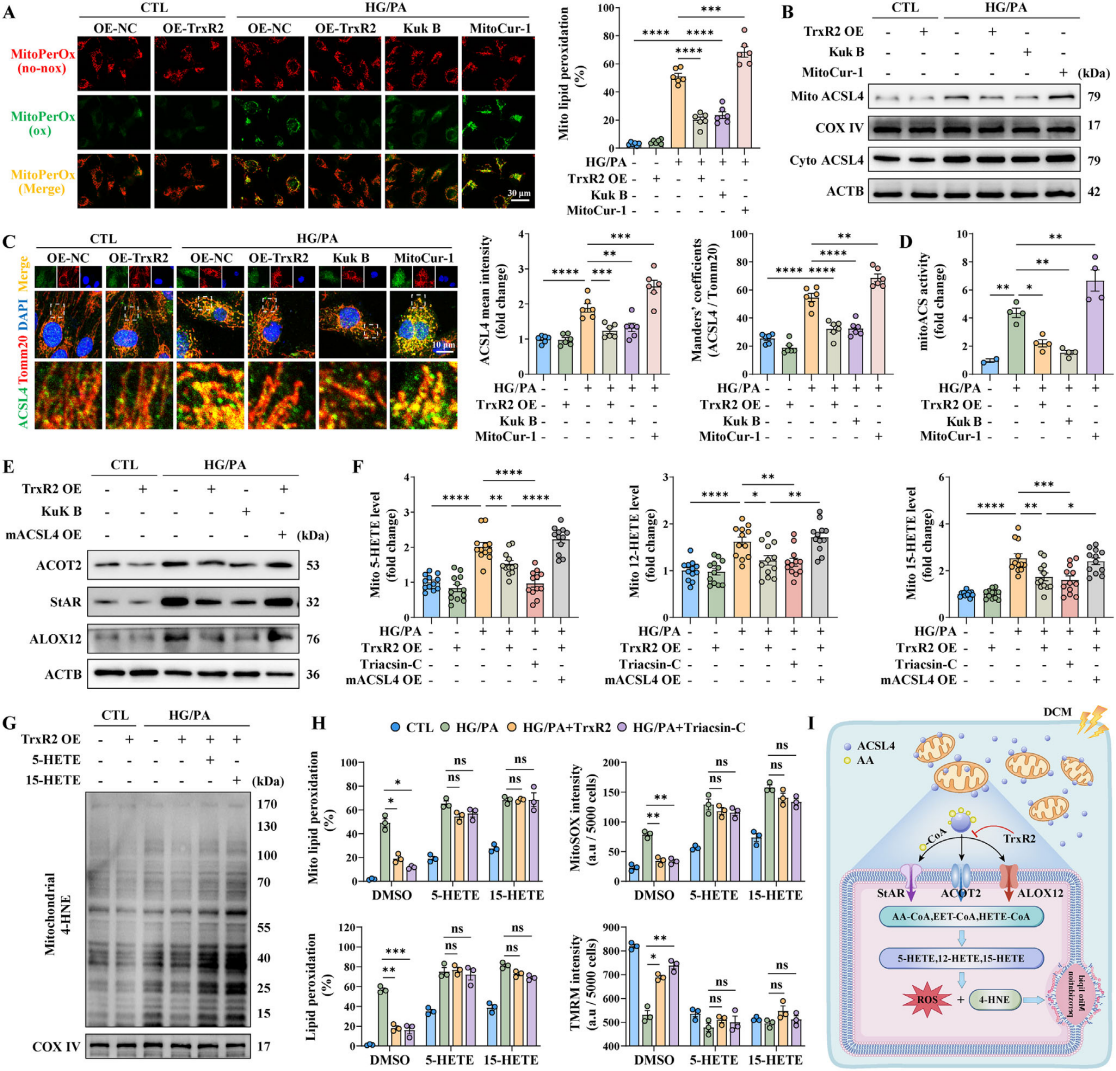
TrxR2 inhibited mitochondria‐associated ferroptosis by inhibiting mitochondrial translocation of ACSL4. (A) Representative immunofluorescence images and quantification data of MitoPerOx staining in the indicated groups (*n* = 6 per group). (B) ACSL4 expression in mitochondrial and mitochondria‐free fractions, as detected by western blotting (*n* = 3 per group). (C) Representative immunofluorescence images of ACSL4 and Tomm20 expression (left), quantification of ACSL4 intensity (middle), and statistical analysis of the colocalization between ACSL4 and Tomm20 using Manders’ coefficients (right) (*n* = 6 per group). (D) Statistical analysis of ACS activity (*n* = 4 per group). (E) ACOT2, StAR, and ALOX12 expression, as detected by western blotting (*n* = 3 per group). (F) Levels of 5‐HETE, 12‐HETE, and 15‐HETE in the mitochondrial fraction were measured using ELISA and statistically analyzed (*n* = 12 per group). (G) 4‐HNE expression in the mitochondrial fraction, as analyzed using western blotting (*n* = 3 per group). (H) Statistical analysis of mitoLPO, cellular LPO, MitoSOX intensity, and TMRM intensity after 72 h of treatment with HG/PA, 5‐HETE (1 µm), and 15‐HETE (1 µm) (*n* = 6 per group). (I) A diagram illustrating that ACSL4 promotes mitoLPO through its downstream signaling pathways—StAR, ACOT2, and ALOX12—as well as the metabolic intermediates 5‐HETE, 12‐HETE, and 15‐HETE. Two‐way ANOVA, followed by Tukey's post‐hoc multi‐comparison test, was used for this figure. *p < 0.05, **p < 0.01, ***p < 0.001, and ****p < 0.0001 indicate statistically significant differences. AA, Arachidonic Acid; DCM, diabetic cardiomyopathy; mACSL4, mitochondrial ACSL4.

Recent studies have emphasized the role of mitoLPO in the regulation of ferroptosis [57, 62]. Consistent with prior findings, the oxidized MitoPerOx significantly increased in HCMECs treated with HG/PA and primary MCMECs sorted from db/db mice, but was reduced by TrxR2 overexpression and Kuk B treatment (Figure 3A; Figure S5A). DHODH and FSP1, which are key regulators of mitochondria‐associated ferroptosis, were unaffected by TrxR2 overexpression or Kuk B treatment (Figure S4I). Therefore, we explored the role of TrxR2 in the regulation of ACSL4 and mitoLPO formation. ACSL4 translocates to mitochondria in diabetes, thereby contributing to DCM development [6, 14]. In the present study, total ACSL4 expression, mitochondrial localization of ACSL4, and mitochondrial ACS activity were clearly enhanced after diabetic injury (Figure 3B–D; Figure S5B). Both TrxR2 overexpression and Kuk B treatment alleviated these effects (Figure 3B–D; Figure S5B). Moreover, mitoACSL4 overexpression abolished the benefits of TrxR2 and Kuk B on mitoLPO suppression and mitochondrial protection in HG/PA‐ and RSL3‐injured HCMECs (Figure S5C–G). Conversely, the ACSL4 inhibitor, Triacsin‐C, mitigated cellular LPO and mitoLPO, and improved mitochondrial function, even after TrxR2 KO (Figure S5H,I). These findings indicate that TrxR2 inhibits mitochondria‐associated ferroptosis by regulating the mitochondrial translocation of ACSL4.

ACSL4 prefers PUFAs such as AA as its substrate. It facilitates the conversion of intracellular free AA into AA‐CoA and its delivery to the mitochondria via ACOT2 [63]. The released AAs are metabolized by lipoxygenases (ALOX12) and StAR for eicosanoid biosynthesis [64, 65]. TrxR2 and Kuk B suppressed the expression of ACOT2, StAR, and ALOX12, and reduced mitochondrial eicosanoid biosynthesis (5‐HETE, 12‐HETE, and 15‐HETE) after HG/PA injury (Figure 3E,F). However, mitoACSL4 overexpression abolished the effects of TrxR2 on eicosanoid biosynthesis (Figure 3E,F). Furthermore, addition of the exogenous eicosanoids 5‐HETE and 15‐HETE increased mitochondrial 4‐HNE expression, promoted LPO and mitoLPO formation, enhanced mitochondrial oxidative stress and dysfunction, and abolished the benefits of TrxR2 and Triacsin‐C under HG/PA injury (Figure 3G,H). Collectively, these results illustrate that TrxR2 suppresses the mitochondrial translocation of ACSL4 to reduce mitochondrial eicosanoid biosynthesis, thereby inhibiting mitochondria‐associated ferroptosis (Figure 3I).

### 3.5 SCP2 Bound to and Facilitated Mitochondrial Translocation of ACSL4

**FIGURE 4:**
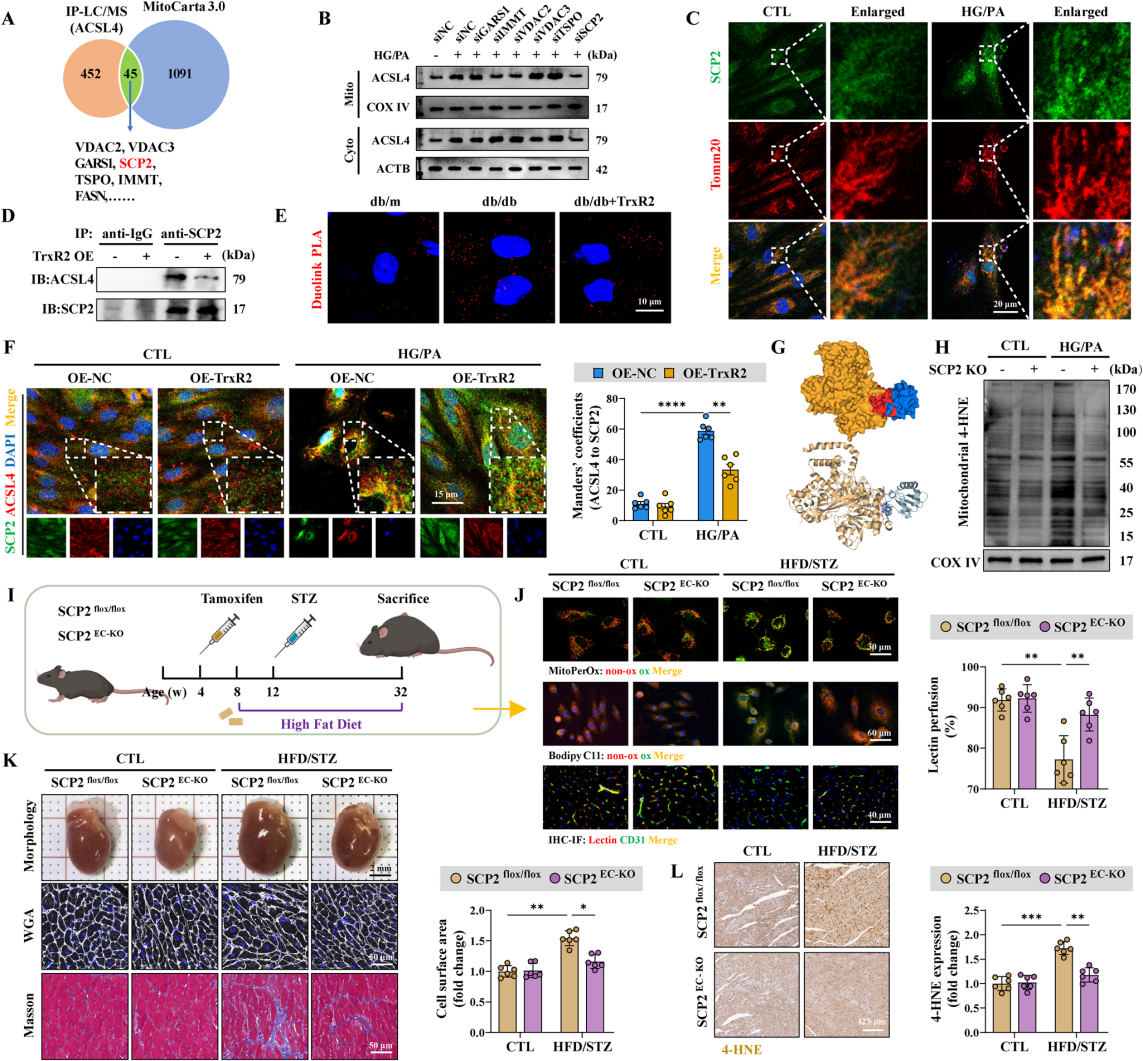
SCP2 was identified as a mitochondrial receptor for ACSL4. (A) A Venn diagram comparing IP‐LC/MS data of ACSL4 in HG/PA‐injured HCMECs and the MitoCarta 3.0 database identified SCP2 as a mitochondrial protein associated with ACSL4. (B) ACSL4 expression in mitochondrial and mitochondria‐free fractions, as detected by western blotting (*n* = 2 per group). (C) Representative immunofluorescence images showing the mitochondrial expression of SCP2 following HG/PA injury. (D) The interaction between SCP2 and ACSL4, as detected by Co‐IP assays in HCMECs following HG/PA treatment and TrxR2 overexpression (*n* = 2 per group). (E) Duolink PLA assay between SCP2 and ACSL4 in primary MCMECs isolated from diabetic db/db mice with or without TrxR2 overexpression (*n* = 2 per group). (F) Representative immunofluorescence images of ACSL4 and SCP2 expression and statistical analysis of their colocalization using Manders’ coefficients (*n* = 6 per group). (G) A predicted docking model revealing the binding mode between ACSL4 (yellow) and SCP2 (blue). (H) Mitochondrial 4‐HNE expression, as detected using western blotting (*n* = 3 per group). (I) A schematic outline demonstrating the induction of T2DM in SCP2^flox/flox^ and SCP2^EC‐KO^ mice by the HFD/STZ method. (J) Representative immunofluorescence images of MitoPerOx and BODIPY C11 staining in primary MCMECs (top and middle) and lectin perfusion assay (bottom) in the indicated mice (*n* = 6 per group). (K) Representative images of cardiac morphology (top), WGA staining (middle), and Masson's staining (bottom) of the indicated mice (*n* = 6 per group). (L) Representative images and statistical analysis of 4‐HNE expression (*n* = 6 per group). Two‐way ANOVA, followed by Tukey's post‐hoc multi‐comparison test, was used for this figure. *p < 0.05, **p < 0.01, ***p < 0.001, and ****p < 0.0001 indicate statistically significant differences. Co‐IP: co‐immunoprecipitation; HFD/STZ, high‐fat diet /streptozotocin; IHC, immunohistochemistry; IP‐LC/MS, immunoprecipitation combined with liquid chromatography‐mass spectrometry; PLA, proximity ligation assay; T2DM, Type 2 diabetes mellitus; WGA, wheat germ agglutinin.

ACSL4 localizes to the mitochondria and mitochondria‐associated membranes [66]. The present study further confirmed its expression in the outer mitochondrial membrane (Figure S6A). ACSL4 is not an intrinsic membrane protein, indicating that it likely translocates to the mitochondria through protein–protein interactions [67]. The TOM/TIM complex facilitates mitochondrial translocation of cytoplasmic proteins [68]. However, the knockout of this complex did not impede ACSL4 translocation (Figure S6B). Therefore, IP‐LC/MS analysis was performed to identify the potential binding receptors for ACSL4 translocation. In total, 45 mitochondrial proteins interacted with ACSL4 (Figure 4A). Of these, SCP2, VDAC2, and IMMT knockdown significantly reduced ACSL4 mitochondrial translocation (Figure 4B). However, only SCP2 expression was upregulated after HG/PA injury (Figure 4C; Figure S6C); this expression was suppressed by TrxR2 overexpression (Figure S6C). In addition, TrxR2 overexpression weakened the binding and colocalization of ACSL4 and SCP2, as reflected in the Co‐IP assay, Duolink PLA assay, and immunofluorescence colocalization analysis (Figu re 4D–F). These data indicate that SCP2 is a receptor for the mitochondrial translocation of ACSL4.

SCP2 was cleaved from its precursor SCPx; however, ACSL4 did not bind to SCPx (Figure S6D). Molecular docking revealed that SCP2 was more suitable for ACSL4 binding than SCPx, with a higher potential hydrogen‐bonding force (Figure 4G; Figure S6E). Next, we examined how SPC2 binds to ACSL4. SCP2 has a mitochondrial translocation peptide (MTP) within the N‐terminal 20 amino acids and a nonspecific lipid‐binding region spanning 33–123 amino acids [69]. Therefore, we generated truncated SCP2 mutants: SCP2△N32 (lacking the MTP), SCP2△N33‐123 (retaining the MTP), and MTP‐SCP2△N32 (SCP2△N32 fused with MTP) (Figure S6F). SCP2△N33‐123 retained mitochondrial localization but failed to bind ACSL4 (Figure S6G,H). SCP2△N32 lost mitochondrial localization and failed to bind to or colocalize with ACSL4 (Figure S6G,I,J). Only MTP‐SCP2△N32 that maintained mitochondrial localization bound to ACSL4 (Figure S6G). However, a GST pull‐down assay, independent of protein localization, confirmed that SCP2△N32 binds to ACSL4 (Figure S6K). These results demonstrate that SCP2 interacts with ACSL4 via its non‐specific lipid‐binding region. Collectively, our data demonstrate that SCP2 does not bring ACSL4 from the cytoplasm to the mitochondria but acts as a mitochondrial receptor for ACSL4 through its non‐specific lipid combination region. Particularly, the interaction between SCP2 and ACSL4 is influenced by diabetes and TrxR2.

### 3.6 SCP2 Promoted Endothelial Ferroptosis in Diabetes

We further investigated whether SCP2 contributes to endothelial ferroptosis under diabetic conditions. SCP2 KO attenuated mitochondrial eicosanoid biosynthesis and repaired mitochondrial injury following HG/PA injury (Figure S7A,B). Importantly, it reduced the mitochondrial 4‐HNE expression and suppressed mitoLPO (Figure 4H; Figure S7C). Similarly, pharmacological inhibition of SCP2 using SCPI2 decreased mitoLPO, whereas the SCP2 agonist 4OHT intensified this effect (Figure S7C). In addition, SCP2 overexpression aggravated LPO and cell death in the RSL3‐established ferroptosis model (Figure S7D,E). SCP2^EC‐KO^ mice were subjected to DCM using the HFD/STZ method for in vivo analyses (Figure 4I; Figure S7F). SCP2 EC‐KO reduced LPO and mitoLPO levels in primary MCMECs and maintained microvascular reperfusion in diabetic mice (Figure 4J; Figure S7G). Moreover, SCP2 EC‐KO enhanced endothelial vasodilation and reduced endothelial inflammation (Figure S7H,I). Additionally, SCP2 EC‐KO mitigated pathological cardiac hypertrophy, fibrosis, and dysfunction (Figure 4K; Figure S7J). Furthermore, SCP2 EC‐KO decreased the ferroptosis marker, 4‐HNE, in the diabetic heart (Figure 4L). These findings highlighted the critical role of SCP2 in the promotion of endothelial ferroptosis and cardiovascular dysfunction in DCM.

### 3.7 TrxR2 Promoted SCP2 Degradation via FUNDC1‐Dependent Mitophagy

We further investigated how TrxR2 promotes SCP2 degradation. TrxR2 destabilized SCP2 and promoted its lysosomal translocation; these effects were abolished by the autophagy inhibitors MA‐3 and Baf.A1, but not by the protease inhibitor MG132 (Figure S8A–D). In addition, the ubiquitin of TrxR2 was unaffected by HG/PA injury (Figure S8E). Mitophagy and chaperone‐mediated autophagy (CMA) are the two major pathways involved in autophagy‐dependent protein degradation. The mitophagy agonist MA‐5 suppressed SCP2 expression, enhanced lysosomal translocation of SCP2, and suppressed mitoACSL4 expression (Figure S8F–H). In contrast, HG/PA injury and TrxR2 overexpression did not affect the CMA of SCP2, as revealed by the unaltered PAmCherry‐SCP2‐NE intensity (Figure S8I). Collectively, these data indicate that TrxR2 may promote SCP2 degradation through mitophagy rather than by CMA or the ubiquitin‐proteasome system.

**FIGURE 5:**
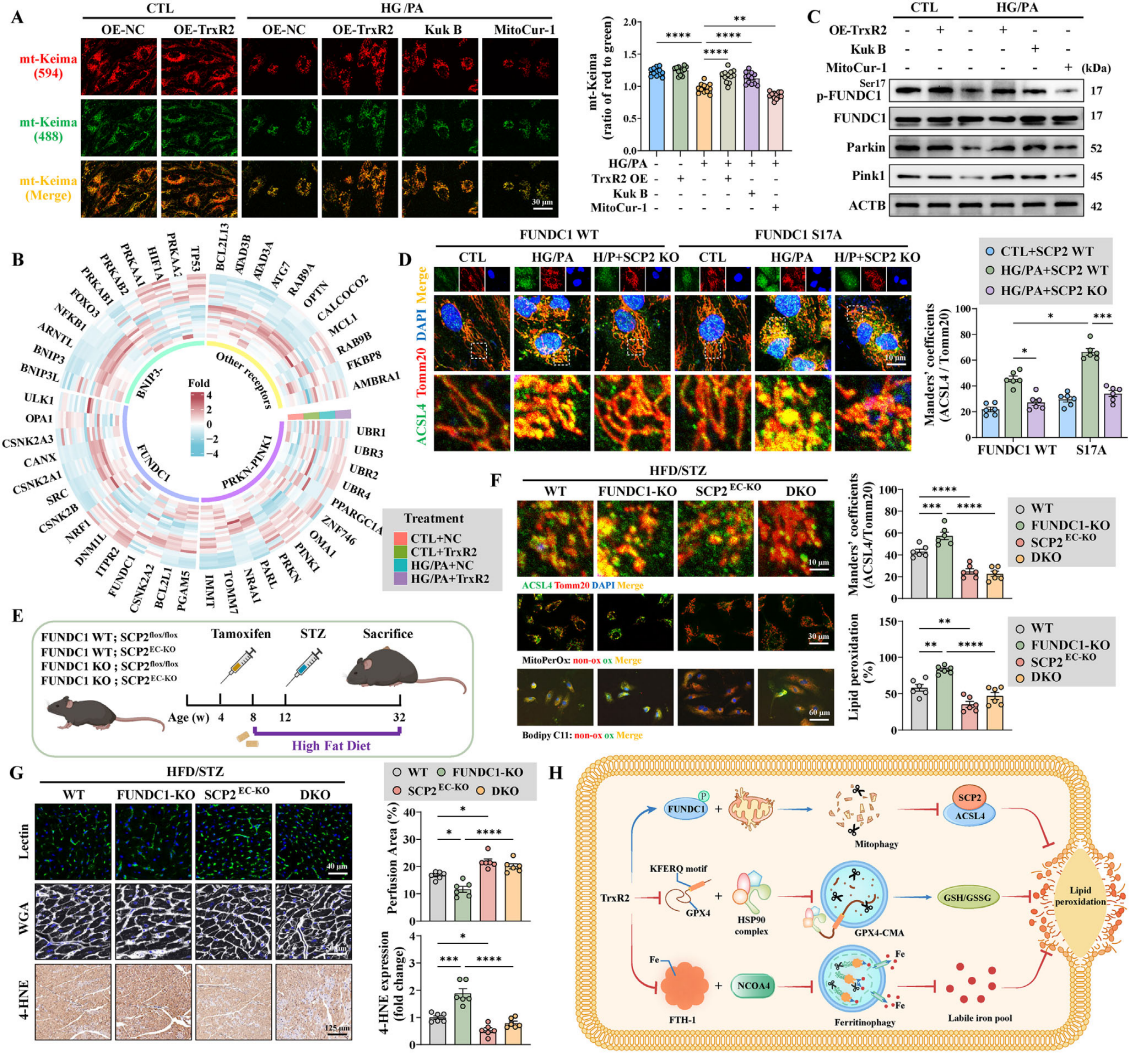
SCP2 was degraded through FUNDC1‐mediated mitophagy. (A) Mitophagy level was indicated by the ratio of red to green fluorescence intensity of mt‐Keima transfection (*n* = 12 per group). (B) A heatmap of mitophagy‐related genes with hierarchical clustering, including PINK1‐Parkin, FUNDC1, BNIP3‐BNIP3L, and other receptor pathways. (C) Western blotting of FUNDC1, Parkin, Pink1 expression, and FUNDC1 phosphorylation at Ser17 (*n* = 3 per group). (D) Representative immunofluorescence images of ACSL4 and Tomm20 expression and statistical analysis of their colocalization using Manders’ coefficients (*n* = 6 per group). (E) A schematic outline showing T2DM introduced into FUNDC1 WT; SCP2^flox/flox^ (WT) mice, FUNDC1 KO; SCP2^flox/flox^ (FUNDC1 KO) mice, FUNDC1 WT; SCP2^EC‐KO^ (SCP2^EC‐KO^) mice, and FUNDC1 KO; SCP2^EC‐KO^ (DKO) mice using the HFD/STZ method. (F) Representative immunofluorescence images of mitoACSL4 (top), MitoPerOx (middle), and BODIPY C11 (bottom) staining of primary MCMECs sorted from the indicated mice (*n* = 6 per group). (G) Representative images of the lectin perfusion assay (top), WGA staining (middle), and 4‐HNE staining (bottom) in the indicated mice (*n* = 6 per group). (H) A diagram illustrating that TrxR2 promotes FUNDC1‐dependent mitophagy while inhibiting the CMA degradation of GPX4 and ferritinophagy, acting synergistically to suppress ferroptosis. Two‐way ANOVA, followed by Tukey's post‐hoc multi‐comparison test, was used for this figure. *p < 0.05, **p < 0.01, ***p < 0.001, and ****p < 0.0001 indicate statistically significant differences. CMA, chaperone‐mediated autophagy.

Compromised endothelial mitophagy contributes to microvascular injury [70]. In the present study, mitophagy was suppressed in HCMECs treated with HG/PA but was re‐activated by TrxR2 overexpression (Figure 5A). Importantly, RNA‐sequencing assay and western blotting revealed that TrxR2 activates mitophagy through the FUNDC1 and Pink1‐Parkin pathways, as evidenced by increased FUNDC1 phosphorylation at Ser17 and increased Pink1 and Parkin expression (Figure 5B,C). We then deleted Parkin and FUNDC1 or mutated FUNDC1 serine 17 to alanine (S17A). FUNDC1 KO or the S17A mutation completely abolished the benefits of TrxR2 on mitophagy and LPO formation, whereas Parkin KO only partially impaired TrxR2 function (Figure S9A–C). Additionally, the Co‐IP assay showed that TrxR2 directly interacted with FUNDC1 rather than with Parkin, confirming that FUNDC1 is a direct target of TrxR2 in mediating mitophagy (Figure S9D).

SCP2 expression was further upregulated in FUNDC1 S17A cells compared with that in WT cells after HG/PA injury; TrxR2 failed to suppress SCP2 expression in the FUNDC1 S17A cell line (Figure S9E). Moreover, mitoACSL4 expression and mitoLPO generation were enhanced in the FUNDC1 S17A cell line after HG/PA injury but were rescued by SCP2 KO (Figure 5D; Figure S9F). Additionally, the FUNDC1 S17A mutation intensified cell death and LPO formation in an RSL3‐established ferroptosis model, which was reversed by SCP2 KO (Figure S9G–I). This demonstrates that SCP2 is a downstream target of FUNDC1 in ferroptosis modulation. Collectively, these findings indicate that TrxR2 promotes SCP2 degradation via FUNDC1‐dependent mitophagy, thereby suppressing mitochondria‐associated ferroptosis.

### 3.8 SCP2 was a Downstream Target of the TrxR2‐FUNDC1 Pathway in Cardiovascular Protection in DCM

FUNDC1 KO, SCP2^EC‐KO^, and DKO mice were generated and subjected to HFD/STZ treatment to further validate the role of the TrxR2‐FUNDC1‐SCP2 pathway in cardiac microvascular ferroptosis and dysfunction in DCM (Figure 5E). mitoACSL4 expression, mitoLPO formation, and LPO levels in primary MCMECs were elevated in FUNDC1 KO mice but reduced in SCP2^EC‐KO^ and DKO mice (Figure 5F; Figure S10A). FUNDC1 KO exacerbated cardiac microvascular injury and pathological remodeling, which was reversed in DKO mice (Figure 5G; Figure S10B). In addition, cardiac and plasma 4‐HNE levels were elevated in FUNDC1 KO mice but were reduced in SCP2^EC‐KO^ and DKO mice (Figure 5G; Figure S10B).

We then performed endothelial‐specific TrxR2 overexpression in FUNDC1 WT and KO mice (Figure S10C). TrxR2 overexpression reduced mitoLPO formation, decreased LPO levels in primary MCMECs, and improved cardiac perfusion in FUNDC1 WT, but not in FUNDC1 KO mice (Figure S10D). In addition, TrxR2 overexpression alleviated pathological cardiac remodeling and mitigated cardiac and plasma 4‐HNE expression in FUNDC1 WT mice but not in FUNDC1 KO mice (Figure S10E‐G). These data establish an essential role of the TrxR2‐FUNDC1‐SCP2 pathway in cardiovascular protection and highlight FUNDC1 as an intermediate molecule through which TrxR2 mitigates SCP2‐associated ferroptosis.

### 3.9 TrxR2 Balanced the Autophagic Process and Selectively Promoted Mitophagy

Ferroptosis is an autophagy‐dependent form of cell death [71]. In addition to mitophagy, CMA and ferritinophagy are other forms of autophagy that regulate ferroptosis [16, 53]. First, CMA‐mediated degradation of GPX4 during ferroptosis was investigated. HG/PA injury enhanced the interaction between GPX4 and molecular chaperones (HSC70, HSP90, and LAMP2a) and promoted the lysosomal translocation of GPX4 (Figure S11A,B). Furthermore, the fluorescent puncta of CMA‐GPX4 reporter (PAmCherry‐GPX4‐NE) increased after HG/PA treatment, which were reduced by TrxR2 overexpression (Figure S11C). These results indicate that TrxR2 suppressed CMA‐mediated GPX4 degradation.

Ferroptosis is driven by iron accumulation via NCOA4‐mediated ferritinophagy, which is a selective autophagic process that releases free iron from ferritins (FTH and FTL) [72]. In this study, ferrous iron levels increased following HG/PA injury; TrxR2 overexpression reversed these effects (Figure S11D). RNA‐sequencing assay revealed a compensatory upregulation of FTH1 and FTL gene expression following HG/PA injury (Figure S11E). However, their protein levels were significantly reduced (Figure S11F). In contrast, TrxR2 increased the protein expression of FTH1 and FTL following HG/PA injury (Figure S11F). These effects were attributed to its role in the alleviation of ferritinophagy, as evidenced by the decreased combination and colocalization of FTH1 and NCOA4 (Figure S11G,H). These findings suggest that TrxR2 balances autophagy and selectively promotes mitophagy, while inhibiting CMA and ferritinophagy to counteract ferroptosis (Figure 5H).

### 3.10 EC‐Released 4‐HNE Contributed to Cardiomyocyte Ferroptosis in Diabetes

**FIGURE 6:**
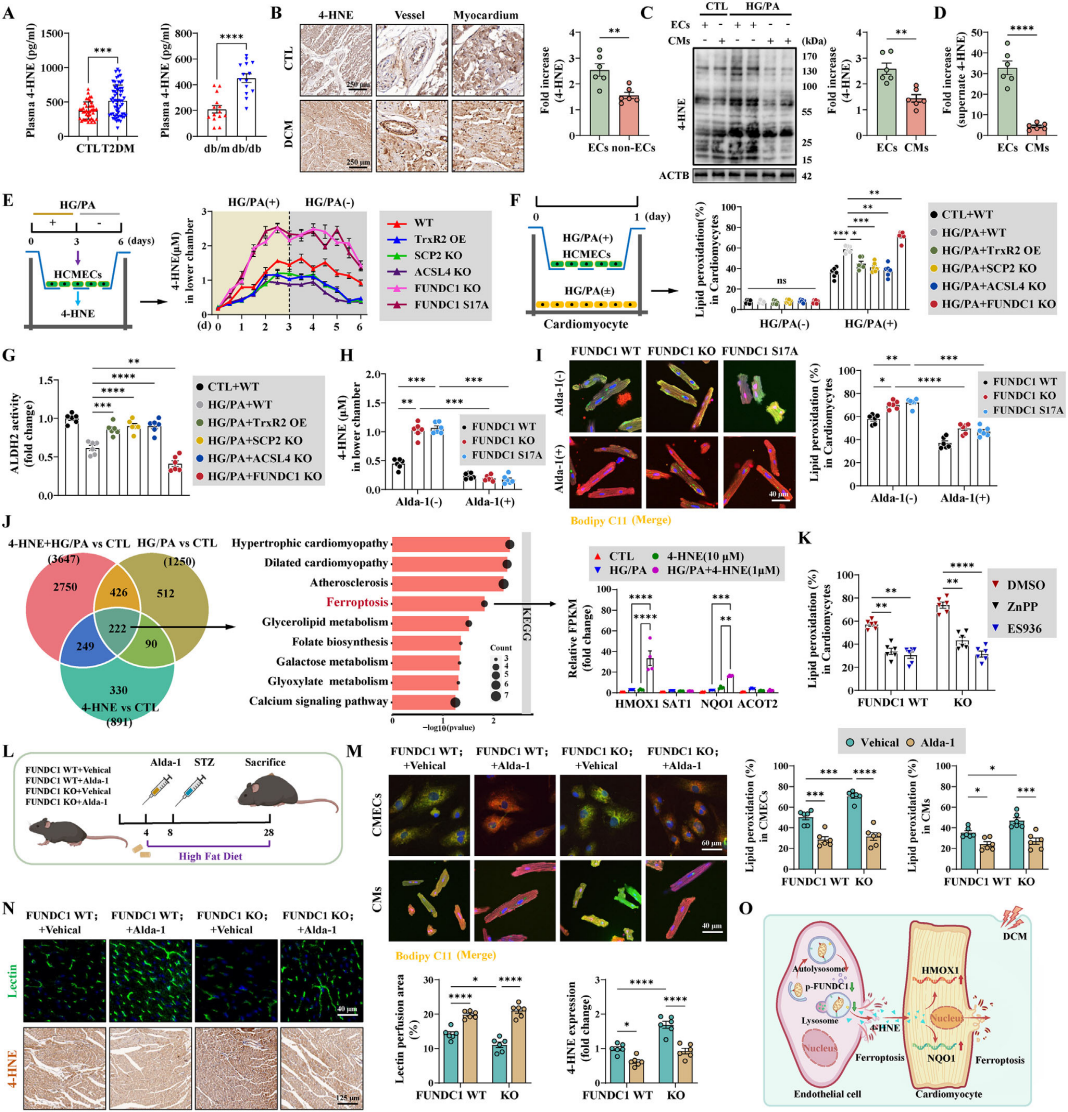
EC‐released 4‐HNE coordinated with diabetes to induce cardiomyocyte ferroptosis via HMOX1 and NQO1. (A) Statistical analysis of plasma 4‐HNE expression in health control (*n* = 46 per group), patients with T2DM (*n* = 81 per group), db/m mice (*n* = 14 per group), and db/db mice (*n* = 14 per group). (B) Representative images of 4‐HNE expression (left panel) in patients with DCM and statistical analysis of the fold increase of 4‐HNE in the vascular and non‐vascular regions (*n* = 6 per group). (C) Fold increase of 4‐HNE in HMCECs and primary mouse cardiomyocytes after HG/PA injury, as detected by western blotting (*n* = 4 per group). (D) Fold increase of the released 4‐HNE in the culture supernatant from HCMECs and primary mouse cardiomyocytes after HG/PA injury (*n* = 6 per group). (E) An illustration showing that HCMECs in the upper chamber of the co‐culture system released 4‐HNE into the lower chamber (left). The concentration of 4‐HNE in the lower chamber was continuously monitored every 12 h for 7 days (right) (*n* = 6 per group). (F) An illustration showing that HG/PA was added to both HCMECs and cardiomyocytes in the co‐culture system; LPO of cardiomyocytes in the lower chamber was detected in the indicated HCMECs groups (*n* = 6 per group). (G) Statistical analysis of ALDH2 activity in HCMECs (*n* = 6 per group). (H) HCMECs were treated with Alda‐1 (10 µm), and the concentration of 4‐HNE released to the lower chamber was monitored for one day (*n* = 6 per group). (I) Representative immunofluorescence images of BODIPY C11 staining and statistical analysis of LPO of cardiomyocytes in the lower chamber (*n* = 6 per group). (J) A Venn diagram showing 222 covariant DEGs among the HG/PA, 4‐HNE, and HG/PA+4‐HNE groups from the RNA‐sequencing assay (left), KEGG enrichment analysis of the 222 covariant DEGs (middle), and relative FPKM expression of *HMOX1*, *SAT1*, *NQO1*, and *ACOT2* (right) (*n* = 4 per group). (K) FUNDC1 WT or FUNDC1 KO HCMECs were cultured in the upper chamber, whereas cardiomyocytes in the lower chamber were pretreated with ZnPP or ES936 for 24 h. Following HG/PA injury, the LPO of cardiomyocytes was statistically analyzed (*n* = 6 per group). (L) A schematic outline illustrating the intraperitoneal injection of Alda‐1 (10 mg/kg/day) into FUNDC1 WT and FUNDC1 KO T2DM mice for 24 weeks. (M) Representative immunofluorescence images of BODIPY C11 staining and statistical analysis of LPO in primary MCMECs (top) and cardiomyocytes (bottom) (*n* = 6 per group). (N) Representative images and statistical analysis of the lectin perfusion assay (top) and 4‐HNE staining (bottom) in the indicated mice (*n* = 6 per group). (O) A diagram illustrating that ECs release 4‐HNE into cardiomyocytes, which, in cooperation with diabetes, facilitate ferroptosis in cardiomyocytes through the transcription of *HMOX1* and *NQO1*. Unpaired Student's t‐test was used for A–D. Two‐way ANOVA, followed by Tukey's post‐hoc multi‐comparison test, was used for E–N. *p < 0.05, **p < 0.01, ***p < 0.001, and ****p < 0.0001 indicate statistically significant differences. Vs, vascular.

4‐HNE, a highly reactive aldehyde generated  from ACSL4‐mediated lipid peroxidation, is a diffusible molecule with paracrine effects. Plasma and cardiac 4‐HNE levels were elevated after long‐term diabetes in patients and mice with T2DM (Figure 6A,B). Subgroup analyses indicated that the plasma levels of 4‐HNE were affected by cardiac function (LVEF), glycosylated hemoglobin (HbA1c%), and the duration of diabetes (Figure S12A). Furthermore, cardiac microvascular protection via TrxR2 overexpression and SCP2 EC‐KO reduced cardiac and plasma 4‐HNE levels (Figures 4L and 5G; Figure S12B). In the present study, ECs were more susceptible to diabetes than cardiomyocytes, leading to higher 4‐HNE expression and secretion in ECs (Figure 6B–D). To elucidate the mechanisms through which TrxR2‐mediated vascular protection alleviates cardiac ferroptosis, we further investigated whether endothelial 4‐HNE is a paracrine mediator of the surrounding CMs using a co‐culture system. HCMECs were placed in the upper chamber of a Transwell plate, whereas primary mouse cardiomyocytes were placed in the bottom chamber (Figure S12C). HG/PA injury stimulated ECs to secrete 4‐HNE into the lower chamber in a time‐dependent manner; these effects were alleviated by ferroptosis inhibition (TrxR2 OE, SCP2 KO, and ACSL4 KO), but enhanced by FUNDC1‐mitophagy inactivation (FUNDC1 KO and FUNDC1 S17A) (Figure 6E). However, 4‐HNE released by ECs did not aggravate LPO formation or cell death in cardiomyocytes, even in the FUNDC1 KO or FUNDC1 S17A EC lines (Figure S12C–E). We further directly treated cardiomyocytes with 4‐HNE (Figure S12F). A minimum of 5 µm 4‐HNE was required to induce LPO, whereas 10 µm 4‐HNE was necessary to consistently induce cardiomyocyte death (Figure S12G,H). These concentrations exceeded those released by ECs (Figure 6E). We hypothesized that EC‐released 4‐HNE cooperates with diabetes to exacerbate ferroptosis in cardiomyocytes. To verify this hypothesis, 4‐HNE was added to CTL or HG/PA‐injured cardiomyocytes. The IC50 of 4‐HNE was significantly lower in HG/PA‐injured cardiomyocytes than in CTL cardiomyocytes (Figure S12I). Additionally, 1 µm 4‐HNE markedly exacerbated LPO formation and cell death in HG/PA‐injured cardiomyocytes (Figure S12).

Based on these results, HG/PA was added to both ECs and cardiomyocytes in the co‐culture system (Figure 6F). TrxR2 OE, SCP2 KO, and ACSL4 KO ECs decreased LPO formation in cardiomyocytes, whereas FUNDC1 KO in ECs exhibited the opposite effect (Figure 6F). These results suggest that EC‐released 4‐HNE coordinates with diabetes to promote cardiomyocyte ferroptosis. Moreover, ALDH2 oxidizes 4‐HNE to 4‐HNA for detoxification. The activity of endothelial ALDH2, but not its expression, was modulated by the TrxR2–FUNDC1–SCP2–ACSL4 pathway (Figure 6G; Figure S12K). Consequently, the ALDH2 agonist Alda‐1 was administered to FUNDC1 KO and FUNDC1 S17A ECs, which decreased EC‐released 4‐HNE and LPO in cardiomyocytes (Figure 6H,I). Collectively, these data demonstrate the synergistic effect of EC‐released 4‐HNE and diabetes in the exacerbation of cardiomyocyte ferroptosis.

### 3.11 4‐HNE Promoted Cardiomyocyte and Cardiac Ferroptosis by Upregulating HMOX1 and NQO1 Expression

We investigated how 4‐HNE contributes to cardiomyocyte and cardiac ferroptosis. RNA‐sequencing assay identified 222 overlapping DEGs among 4‐HNE‐, HG/PA‐, and 4‐HNE+HG/PA‐treated cardiomyocytes (Figure 6J). KEGG and GO enrichment analyses of these DEGs revealed ferroptosis as a significantly enriched pathway, with *HMOX1*, *SAT1*, *NQO1*, and *ACOT2* identified as related genes (Figure 6J; Figure S12L). *HMOX1* and *NQO1* expression was markedly upregulated in 4‐HNE+HG/PA‐treated cardiomyocytes compared to that in 4‐HNE‐ or HG/PA‐treated cardiomyocytes (Figure 6J). Moreover, the pretreatment of cardiomyocytes with the HMOX1 inhibitor ZnPP or the NQO1 inhibitor ES936 alleviated cardiomyocyte ferroptosis in the co‐culture system (Figure 6K).

The contribution of 4‐HNE to DCM was examined in FUNDC1 KO mice treated with Alda‐1 (Figure 6L). LPO formation in primary MCMECs and cardiomyocytes further increased in FUNDC1 KO mice; Alda‐1 treatment significantly alleviated these effects (Figure 6M). Importantly, Alda‐1 improved cardiac microvascular perfusion and suppressed cardiac 4‐HNE expression in both FUNDC1 WT and KO mice (Figure 6N). Moreover, the plasma levels of 4‐HNE were significantly upregulated in FUNDC1 KO mice, accompanied by increased cardiac mRNA expression of *HMOX1* and *NQO1*, which was markedly alleviated by Alda‐1 (Figure S12M,N). These findings comprehensively highlight the critical role of endothelial‐released 4‐HNE in synergizing with diabetes to induce cardiomyocyte ferroptosis in DCM (Figure 6O).

### 3.12 TrxR2 Regulated FUNDC1 Phosphorylation and Mitophagy through the Mitochondrial TUFM‐AMPK Pathway

**FIGURE 7:**
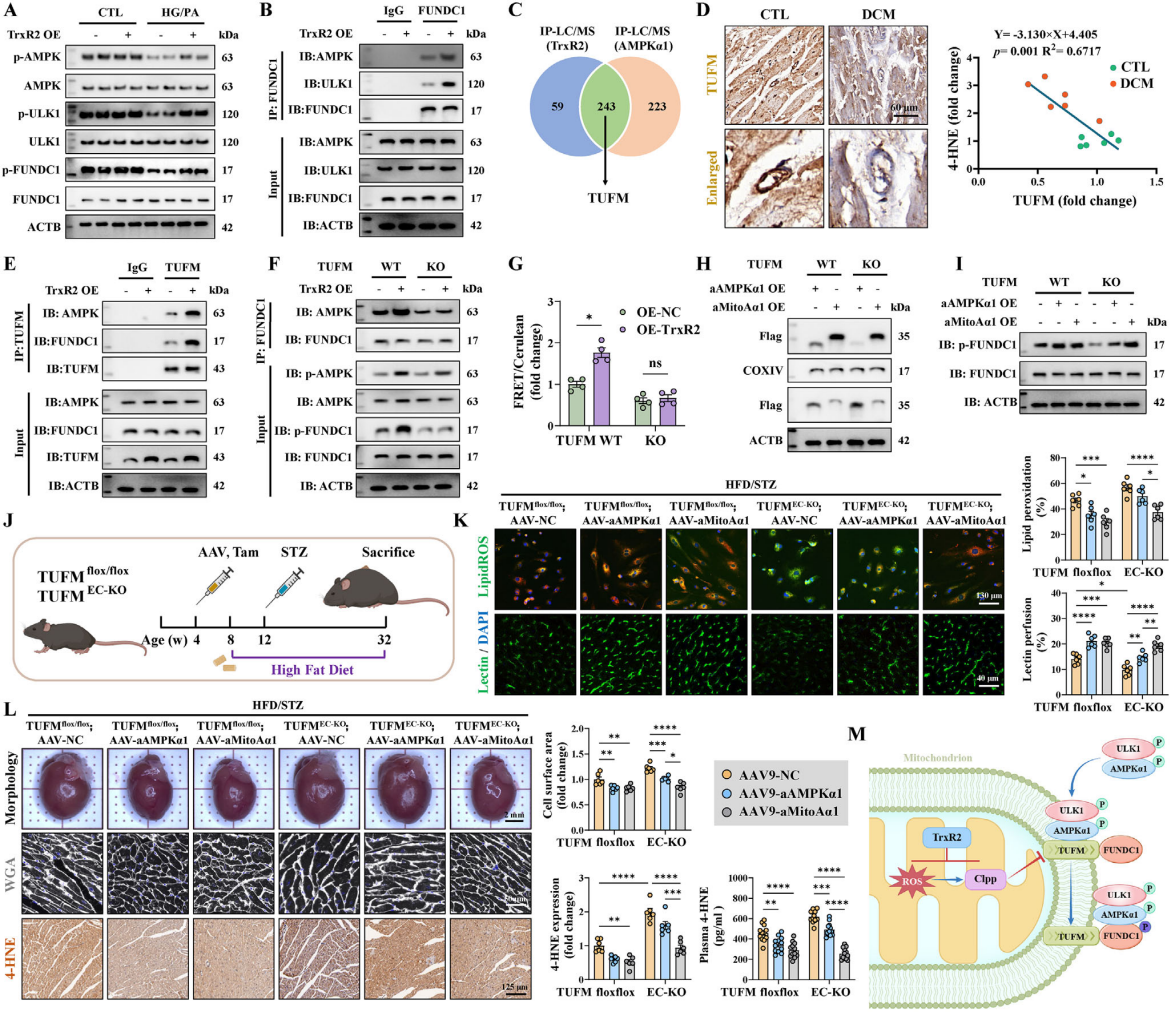
TrxR2 maintained FUNDC1‐dependent mitophagy via the mitochondrial TUFM‐AMPK pathway. (A) The expression and phosphorylation of AMPK, ULK1, and FUNDC1 were analyzed by western blotting (*n* = 4 per group). (B) The interactions among FUNDC1, ULK1, and AMPK were detected using the Co‐IP assay in HG/PA‐injured HCMECs (*n* = 3 per group). (C) A Venn diagram of 243 proteins shared by TrxR2 and AMPK, including TUFM, as analyzed based on the IP‐LC/MS data. (D) IHC staining of TUFM expression in DCM specimens. The relationship between endothelial TUFM and 4‐HNE was analyzed using a linear regression model (*n* = 6 per group). (E) The interactions among FUNDC1, TUFM, and AMPK, as detected using the Co‐IP assay in HG/PA‐injured cells (*n* = 3 per group). (F) The Co‐IP assay was performed to examine the interaction between FUNDC1 and AMPK in TUFM KO cells with or without TrxR2 overexpression (*n* = 3 per group). (G) The activity of mitoAMPK after TUFM KO was determined by pmitoABKAR (*n* = 4 per group). (H) HCMECs were transfected with aAMPKα1‐Flag and aMitoAα1‐Flag, with or without TUFM KO. The distribution of Flag in mitochondrial and mitochondria‐free fractions was detected by western blotting (*n* = 2 per group). (I) FUNDC1 phosphorylation at Ser17 was detected by western blotting after aAMPKα1‐Flag and aMitoAα1‐Flag transfection (*n* = 2 per group). (J) A schematic outline illustrating that TUFM^flox/flox^ and TUFM^EC‐KO^ mice were transfected with aAMPKα1 or aMitoAα1, followed by the induction of DCM using the HFD/STZ method. (K) Representative fluorescence images of BODIPY C11 staining in primary MCMECs (top) and lectin perfusion assays (bottom) in the indicated mice (*n* = 6 per group). (L) Representative images of the cardiac morphology (top), WGA staining (middle), and 4‐HNE staining (bottom) of the indicated mice (*n* = 6 per group) are shown. (M) A diagram illustrating that TUFM transports AMPK to FUNDC1 for FUNDC1 phosphorylation, whereas TrxR2 maintains TUFM expression by inhibiting ROS and Clpp. Two‐way ANOVA, followed by Tukey's post‐hoc multi‐comparison test, was used for this figure *p < 0.05, **p < 0.01, ***p < 0.001, and ****p < 0.0001 indicate significant differences. Tam, tamoxifen; aAMPKα1, activated AMPKα1; aMitoAα1, activated mitochondrial translocation peptide‐fused AMPKα1.

Having demonstrated the role of FUNDC1‐dependent mitophagy in regulating the SCP2/ACLS4 pathway and endothelial ferroptosis, we investigated how TrxR2 modulates its phosphorylation. FUNDC1 phosphorylation is regulated by the canonical AMPK pathway [73]. The mitochondrial AMPK (mitoAMPK) pool is involved in the regulation of mitophagy and ferroptosis [14, 54]. In this study, TrxR2 enhanced the AMPK‐ULK1‐FUNDC1 pathway and increased mitoAMPK activity after HG/PA injury (Figure 7A,B; Figure S13A). However, mitoAIP, which competes with mitoAMPK [54], disrupted the interaction between AMPK and FUNDC1, leading to reduced FUNDC1 phosphorylation (Figure S13B,C). These findings highlighted the critical role of mitoAMPK in FUNDC1‐dependent mitophagy. TrxR2 did not bind directly to AMPK (Figure S13D). However, IP‐LC/MS using antibodies against TrxR2 and AMPK identified TUFM, a key player in mitochondrial protein translation, as an intermediate signal candidate (Figure 7C). TUFM expression was suppressed in human diabetic hearts, db/db mice, and HG/PA‐injured HCMECs following induction of diabetes (Figure 7D; Figure S13E,F). Contrastingly, TrxR2 overexpression increased TUFM expression and enhanced the TUFM‐AMPK‐FUNDC1 interaction (Figure 7E; Figure S13F). In addition, TUFM KO led to decreased mitoAMPK expression and activity and reduced FUNDC1 phosphorylation, even after TrxR2 overexpression (Figure 7F,G; Figure S13G,H). These data suggest that TUFM is activated as an intermediate signal for TrxR2 to regulate the mitochondrial translocation of AMPK and FUNDC1 phosphorylation.

For further validation, we transfected exogenous flag‐tagged activated AMPKα1 (aAMPKα1) and MTP‐fused aAMPKα1 (aMitoAα1) into TUFM KO cells. aMitoAα1 retained its mitochondrial localization and phosphorylated FUNDC1 even in TUFM KO cells (Figure 7H,I). In contrast, the mitochondrial translocation of aAMPKα1 was nearly absent in TUFM KO cells (Figure 7H). Although aAMPKα1 still phosphorylated FUNDC1 in TUFM KO cells, its effects were significantly lower than those of aMitoAα1 (Figure 7I).

TUFM is a target of caseinolytic protease (Clpp), a mitochondrial oxidative stress response gene [74]. Therefore, we evaluated whether TrxR2 maintains TUFM expression by eliminating oxidative stress and suppressing Clpp expression. TrxR2 reduced Clpp expression, whereas mitoLND, an ROS agonist, upregulated Clpp expression and suppressed that of TUFM (Figure S13I). We also confirmed that the administration of radical‐trapping antioxidant mitoTEMPO or the knockout of Clpp strongly enhanced TUFM expression (Figure S13J).

### 3.13 AMPK Alleviated DCM Primarily through TUFM‐Mediated Mitochondrial Translocation

aMitoAα1 enhanced mitoAMPK activity more effectively than aAMPKα1 in both TUFM WT and KO cells (Figure S13K,L). Additionally, aMitoAα1 effectively enhanced ALDH2 activity, reduced EC‐released 4‐HNE, and alleviated cardiomyocyte ferroptosis in the co‐culture system (Figure S13M). In contrast, aAMPKα1 did not exert comparable benefits to those of aMitoAα1 in both TUFM WT and KO cells (Figure S13K–M). TUFM EC‐KO exacerbated endothelial LPO formation, microvascular dysfunction, and pathological hypertrophy in diabetic HFD/STZ mice (Figure 7J–L; Figure S13N). Additionally, plasma and cardiac levels of 4‐HNE were elevated in TUFM EC‐KO mice (Figure 7L). Although overexpression of aAMPKα1 and aMitoAα1 demonstrated comparable effects in improving endothelial LPO formation, microvascular dysfunction, and cardiac ferroptosis in TUFM^flox/flox^ mice, aMitoAα1 was more effective than aAMPKα1 in TUFM^EC‐KO^ mice (Figure 7K,L). Collectively, our data suggest that TrxR2 maintains TUFM expression to facilitate the mitochondrial translocation of AMPK, thereby promoting FUNDC1 phosphorylation and alleviating ferroptosis (Figure 7M).

### 3.14 The Lactylation of TrxR2 Enhanced mitoTrxR Activity in Diabetes

**FIGURE 8:**
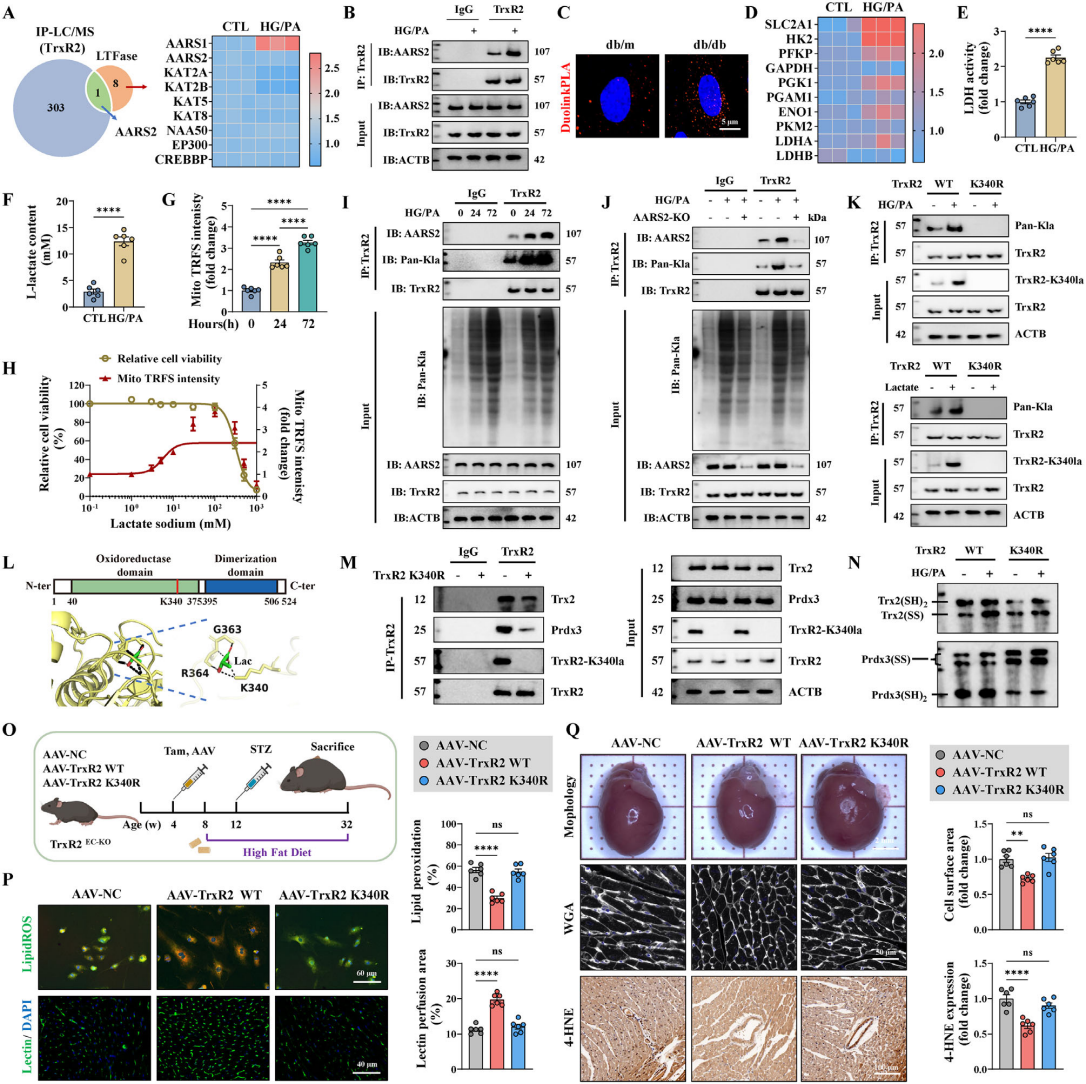
The lactylation modification of TrxR2 at K340 enhanced endothelial mitoTrxR activity in diabetic cardiomyopathy. (A) IP‐LC/MS analysis of TrxR2 in HCMECs revealed that AARS2 binds to TrxR2 (left), whereas RNA‐sequencing assay showed no change in the relative FPKM of AARS2 (right). (B,C) The interaction between TrxR2 and AARS2, as confirmed with a Co‐IP assay in HCMECs challenged with HG/PA and by a Duolink PLA assay in primary MCMECs sorted from db/m and db/db mice (*n* = 2 per group). (D) A heatmap of glycolytic genes from the RNA‐sequencing assay. (E,F) Quantitative assessment of LDH activity and L‐lactate content in HCMECs after HG/PA injury (*n* = 6 per group). (G) Time‐course analysis of MitoTRFS intensity in response to sodium lactate treatment (30 mm) (*n* = 6 per group). (H) Dose‐response curves illustrating the effects of sodium lactate on cell viability and MitoTRFS intensity after 72 h of treatment (*n* = 3 per group). (I,J) The effects of HG/PA injury (left) and AARS2 knockout (right) on pan‐lactylation of cell lysates and TrxR2 were analyzed using Co‐IP assay (*n* = 2 per group). (K) TrxR2 lactylation under HG/PA injury (top) and lactate treatment (bottom), as analyzed using a Co‐IP assay with pan‐lactylation and custom‐made TrxR2 K340la antibodies in TrxR2 WT and TrxR2 K340R cell lines (*n* = 2 per group). (L) A structural chart showing K340 in the oxidoreductase domain of TrxR2. (M) The interactions among TrxR2, Trx2, and Prdx3 in the TrxR2 WT and TrxR2 K340R cell lines, as confirmed using the Co‐IP assay (*n* = 2 per group). (N) Trx2(SH)_2_ (reduced Trx2), Trx2(SS) (oxidized Trx2), Prdx3(SS) (oxidized Prdx3), and Prdx3(SH)_2_ (reduced Prdx3), as detected using redox western blot analysis (*n* = 4 per group). (O) A schematic outline illustrating that TrxR2^EC‐KO^ mice were transfected with TrxR2 WT or TrxR2 K340R using AAV transfection, followed by induction of T2DM using the HFD/STZ method. (P) Representative immunofluorescence images of BODIPY C11 staining in primary MCMECs (top) and lectin perfusion assay (bottom) in the indicated mice (*n* = 6 per group). (Q), Representative images of cardiac morphology (top), WGA staining (middle), and 4‐HNE staining (bottom) of the indicated mice (*n* = 6 per group). Unpaired Student's t‐test was used for E and F. One‐way ANOVA, followed by Tukey's post‐hoc multi‐comparison test, was used for G, P, and Q. Two‐way ANOVA, followed by Tukey's post‐hoc multi‐comparison test, was used for H. *p < 0.05, **p < 0.01, ***p < 0.001, and ****p < 0.0001 indicate statistically significant differences. Pan‐Kla, pan‐lysine lactylation; LTFase, lactyltransferase.

The gene and protein expression of endothelial TrxR2 were unaffected by diabetes both in vivo and in vitro (Figure S14A–C). Therefore, the mechanism through which diabetes increases mitoTrxR activity remains unclear. IP‐LC/MS analysis of TrxR2 revealed its interaction with AARS2, a mitochondrial lysine lactyltransferase (Figure 8A). The mRNA and protein expression of AARS2 was unaffected by diabetes (Figure 8A,B). However, its interaction with TrxR2 was significantly enhanced, as evidenced by the Co‐IP assay and Duolink PLA (Figure 8B,C). Anaerobic glycolysis increases lactate production through the LDH‐catalyzed conversion of pyruvate and further activates lactylation modification with lactyltransferase. In the present study, diabetes activated the anaerobic glycolysis pathway in primary MCMECs and HG/PA‐injured HCMECs (Figure 8D; Figure S14D). Moreover, HG/PA injury enhanced LDH activity, intensified anaerobic glycolysis, and promoted lactate accumulation in HCMECs (Figure 8E,F; Figure S14E). Lactate (<100 µm) enhanced mitoTrxR activity in a dosage‐ and time‐dependent manner (Figure 8G,H). Therefore, both AARS2 and lactate accumulation create conditions conducive to the lactylation of TrxR2.

To further confirm the lactylation of TrxR2, a Co‐IP assay of TrxR2 followed by immunoblotting with a pan‐Kla antibody was performed, which demonstrated that TrxR2 was lactylated under HG/PA and lactate treatment in a time‐dependent manner (Figure 8I; Figure S14F). However, these effects were largely negated in AARS2 KO cells (Figure 8J; Figure S14G). IP‐LC/MS analysis revealed that TrxR2 is lactylated at K340, a residue conserved across different species (Figure S14H). We then developed an anti‐TrxR2 K340la‐specific antibody, which was validated by dot blotting (Figure S14I). Both lactyltransferase overexpression and in vitro lysine lactylation assays confirmed that the AARS2 mediated TrxR2 lactylation at K340, rather than AARS1 (Figure S14J,K).

TrxR2 K340la levels were elevated in DCM samples, diabetic db/db mice, and in HCMECs following HG/PA injury or lactate treatment (Figure 8K; Figure S14L). However, the TrxR2 K340R mutation (lysine 340 replaced with arginine) via the CRISPR‐Cas9 system negated the lactylation of TrxR2 (Figure 8K; Figure S14M). TrxR2 induces oxidant detoxification by reducing Trx2 and Prdx3 [60]. K340 is located in the oxidoreductase domain of TrxR2 (Figure 8L). Therefore, we also assessed the effect of K340 lactylation on TrxR2 function. The TrxR2 K340R mutation abolished interactions between TrxR2, Trx2, and Prdx3 (Figure 8M). In addition, the ratio of the oxidized form to the reduced form of Trx2 and Prdx3 was enhanced after TrxR2 K340R treatment, indicating a compromised reducing capacity of TrxR2 (Figure 8N). Moreover, mitoTrxR activity was enhanced in a time‐dependent manner in TrxR2 WT cells under HG/PA and lactate treatments. However, TrxR2 K340R abolished these effects (Figure S14N). In addition, the reduction of lactate generation with LDH inhibitors (FX‐11 and OA) suppressed mitoTrxR activity under HG/PA and lactate treatments (Figure S14O). These data collectively demonstrate that lactate accumulation and TrxR2 lactylation enhance mitoTrxR activity in response to diabetes.

### 3.15 Lactate and TrxR2 K340la Alleviated Endothelial Ferroptosis and Cardiac Microvascular Injury in Diabetes Models

Based on the above findings, we hypothesized that TrxR2 K340la and lactate contribute to mitophagy and enhance ferroptosis resistance. First, mitophagy was further reduced after TrxR2 K340R mutation (Figure S15A). In addition, TrxR2 K340R mutation aggravated mitoLPO and LPO formation following HG/PA injury (Figure S15B,C). EC‐released 4‐HNE was further increased after the TrxR2 K340R mutation, resulting in increased *HMOX1* and *NQO1* mRNA expression in co‐cultured cardiomyocytes (Figure S15D–F). Similar results were observed upon reduction of lactate production by the LDH inhibitor FX‐11 in both TrxR2 WT and TrxR2 K340R cells (Figure S15B–F).

In HFD/STZ‐induced DCM, TrxR2 WT and K340R were re‐expressed in TrxR2^EC‐KO^ mice (Figure 8O). TrxR2 WT, but not TrxR2 K340R, reduced LPO formation in primary MCMECs, enhanced microvascular perfusion, and mitigated cardiac hypertrophy and ferroptosis (Figure 8P,Q). We further assessed the role of lactate accumulation in DCM. NaLa administration in diabetic db/db mice significantly enhanced mitoTrxR activity and reduced LPO in primary HCMECs, thereby improving cardiovascular function (Figure S15G–J). Conversely, reducing lactate production via FX‐11 suppressed endothelial mitoTrxR activity, increased LPO levels, and exacerbated DCM (Figure S15K–N). Collectively, these findings demonstrate that lactate accumulation and TrxR2 lactylation improve endothelial ferroptosis resistance and enhance cardiovascular function in DCM.

## 4 Discussion

The thioredoxin superfamily regulates cellular redox balance and has the potential to inhibit ferroptosis by facilitating oxidant detoxification. In the present study, we identified TrxR2 as a potent anti‐ferroptotic gene. It prevented mitochondrial lipid peroxidation by activating FUNDC1‐dependent mitophagy to degrade SCP2, a newly identified receptor for ACSL4 mitochondrial recruitment, thereby mitigating mitochondria‐associated ferroptosis. TrxR2 reduced ROS production and suppressed Clpp activation to stabilize TUFM, consequently enabling AMPK‐mediated FUNDC1 phosphorylation and mitophagy. Additionally, we identified a direct link among lactate accumulation, TrxR2 lactylation, and enhanced mitoTrxR activity in diabetes. This link was mediated by AARS2, which acts as a lactyltransferase for TrxR2 lactylation at K340. Therapeutically, TrxR2 and its agonist (Kuk B) enhance ferroptosis resistance, improve cardiac microvascular function, and ameliorate DCM in chronic diabetes.

TrxR2, a rate‐limiting enzyme in the thioredoxin system, alleviates HFD‐induced metabolic dysfunction by enhancing mitochondrial metabolic systems and maintains endothelial homeostasis by alleviating mitochondrial oxidative stress [25, 75]. In this study, TrxR2 improved endothelial and cardiac microvascular injury in DCM, largely because it enhances endothelial ferroptosis resistance by suppressing LPO and 4‐HNE formation. 4‐HNE is one of the most bioactive LPO products. It modulates ferroptosis, mainly through the formation of covalent adducts with proteins [76]. Importantly, 4‐HNE exerts paracrine effects and acts as a paracrine mediator of harmful intercellular communication. For example, the foam cell‐derived 4‐HNE induces endothelial senescence [77]. Our study showed that endothelial 4‐HNE could be released into the plasma and CMs, further coordinating diabetes to promote CM ferroptosis and DCM via the upregulation of *HMOX‐1* and *NQO‐1* expression. These two pro‐ferroptotic genes aggravate heart failure [78, 79]. In contrast, TrxR2‐mediated endothelial protection and ferroptosis resistance interrupt this unfavorable feedback and delay DCM.

Mitochondria are essential for cellular bioenergetics and are the main sites of ROS, LPO, and 4‐HNE production under pathological conditions [76]. Mitochondria‐localized GPX4 and its parallel ferroptosis defense systems such as FSP1 and DHODH trap lipid peroxyl radicals and reduce mitochondria‐associated ferroptosis [9, 57]. In contrast, ACSL4 translocates to the mitochondria and increases LPO [6]. ACSL4 is primarily identified as a ligase that binds free fatty acids to CoA, enabling their entry into the mitochondria for β‐oxidation. However, the reason ACSL4 prefers mitochondrial translocation remains unclear. We identified numerous mitochondrial proteins that bind to ACSL4 during diabetes; SCP2 has been suggested as a receptor for ACSL4. SCP2 is a non‐specific lipid transfer protein that facilitates phospholipid trafficking and mitochondrial lipid transfer [80]. SCP2 also aids in lipid hydroperoxide transfer, the exchange of oxidized derivatives, and the formation of LPO, thereby promoting atherosclerosis and vascular inflammation [81, 82]. Additionally, the C‐terminal domain of SCP2 may act as a scaffold for protein–protein recognition and incorporation [83]. These functions may help explain its potential role as a receptor for ACSL4, as it could aid in the translocation and integration of ACSL4 within the mitochondria, particularly under conditions such as diabetes, where lipid metabolism is altered. Understanding this interaction may provide insights into the regulation of mitochondrial function and LPO formation in metabolic diseases.

FUNDC1 is a key receptor‐mediated pathway for mitophagy that initiates autophagosome formation through its LC3 interacting region [84]. Deficiency in FUNDC1 impairs mitophagy and mitochondrial quality, thereby exacerbating obesity‐induced cardiomyopathy [85]. In this study, TrxR2 promoted mitophagy primarily through FUNDC1. This led to SCP2 degradation and the inhibition of ferroptosis. FUNDC1‐induced mitophagy is regulated by several dynamic phosphorylation and dephosphorylation events. In particular, AMPK‐ULK1 acts as an upstream kinase that phosphorylates FUNDC1 at Ser17 to enhance mitophagy [86]. Additionally, TUFM is crucial for mitophagy and protection against diabetes‐ and doxorubicin‐induced cardiomyopathy [87, 88]. Our research demonstrates that TUFM enriches the mitochondrial AMPK pool in diabetes and facilitates the subsequent phosphorylation of FUNDC1 to enhance mitophagy. In this study, we provide comprehensive and systematic evidence that TrxR2 reduces TUFM degradation via its inherent antioxidation to inhibit Clpp, and underscores the importance of TUFM in enhancing mitochondrial AMPK activity, promoting mitophagy and protecting against diabetic microvascular dysfunction.

Metabolic changes during cardiovascular disease, such as lactate accumulation, modify protein function through post‐translational modifications and contribute to the progression of heart failure [89]. Lactate and lactylation modification affect cardiac prognosis based on modifiable proteins [38]. Histone H3K18 lactylation in monocyte macrophages activates cardiac repair after myocardial infarction [90, 91]. Additionally, exercise‐induced endothelial Mecp2 lactylation suppresses atherosclerosis [45]. In contrast, lactate can also have adverse effects as it promotes endothelial‐to‐mesenchymal transition through Snail, thereby exacerbating conditions such as myocardial infarction [92]. Our data reveal that endothelial lactate accumulation in DCM promotes TrxR2 K340 lactylation, the modification of which further enhances mitoTrxR activity to improve mitophagy, ferroptosis resistance, and cardiac microvascular function. In addition to lactate, protein lactylation requires lysine lactyltransferases. AARS1 and AARS2 have been identified as both sensors for L‐lactate and as lactyltransferases involved in global lysine lactylation [36]. AARS2 is localized to the mitochondria; it lactylates PDHA1 and CPT2 during oxidative phosphorylation [93]. Mutations or deficiencies in AARS2 can lead to lethal mitochondrial cardiomyopathy [94]. In this study, we identified AARS2 as a lactyltransferase involved in TrxR2 K340 lactylation. These findings support the potential role of AARS2 in mitochondrial resilience, ferroptosis resistance, and cardiovascular protection against diabetes.

Although our study identified TrxR2 lactylation as a key mechanism in DCM, several limitations and future directions should be noted. First, although our findings demonstrated that short‐term low‐dose supplementation with exogenous lactate can ameliorate cardiac dysfunction, its optimal dosage, long‐term efficacy, and safety warrant further exploration. This is particularly crucial, as some studies suggest that elevated lactate levels promote DCM [95]. Thus, further investigation is required to resolve this contradiction. Secondly, the clinical utility of TrxR2 lactylation as a direct diagnostic marker is yet to be established. However, its downstream signaling molecule—4‐HNE—shows promise as a potential biomarker of diabetic myocardial injury and warrants further clinical validation. Moreover, our research primarily explored the lactylation of TrxR2 in ECs. Therefore, data on TrxR2 lactylation in non‐endothelial cells (e.g., cardiomyocytes and fibroblasts) are lacking. Finally, TrxR1, a paralog of TrxR2, is more abundant in cardiomyocytes and inhibits ferroptosis. Therefore, whether TrxR1 also undergoes lactylation in DCM warrants further investigation. If so, whether this modification contributes to the endogenous protective mechanisms of the heart must be determined. Addressing these questions will deepen our understanding of lactate signaling in cardiac pathology and may facilitate the development of new therapeutic and diagnostic strategies for DCM.

## 5 Conclusions

In the present study, TrxR2 improved cardiac microcirculation and function in diabetes. TrxR2 maintained the mitochondrial AMPK pool via TUFM to facilitate FUNDC1‐dependent mitophagy, consequently inhibiting the SCP2‐ACSL4‐ferroptosis pathway. Our data also suggest that enhancing mitoTrxR activity through TrxR2 lactylation or pharmacological activation offers an alternative therapeutic strategy for diabetic complications characterized by mitochondria‐associated ferroptosis. These findings may facilitate the development of new therapeutic and diagnostic strategies for DCM.

## Funding

This study was supported by National Natural Science Foundation of China (grant nos. 82570495, 82200449, 82300479, 82300444, and 82403430), China Postdoctoral Science Foundation (grant nos. 2023M731763 and 2024T170427), the Scientific Research Project of the Gusu Health Talent Plan (grant no. GSWS2023108), Nanjing Medical University Science and Technology development project (grant no. NUMB20230220), and Suzhou Science and Technology Innovation Project (grant nos. SYW2024029 and SYW2024125). Natural Science Foundation of Jiangsu Province (grant no. BK20251763). Science and Technology Talent Trusted Project of Jiangsu Province (grant no. JSTJ‐2024‐339). Tianjin Key Medical Discipline Construction Project (Grant No.TJYXZDXK‐3‐003A).

## Disclosure

The authors have nothing to report.

## Conflicts of Interest

The authors declare no conflict of interest.

## Supporting information




**Supporting File**: advs74337‐sup‐0001‐SuppMat.docx.

## Data Availability

The data that support the findings of this study are available from the corresponding author upon reasonable request.
