# Supplementary material for: TrxR2 Lactylation Facilitates Mitochondrial Protection and Endothelial Ferroptosis Resistance in Diabetic Cardiomyopathy
Source: Adv Sci (Weinh). 2026 Feb 17;13(22):e21997. doi: 10.1002/advs.202521997 (PMC13088291; doi:10.1002/advs.202521997)
Supplement: Supplementary file 1 — Supporting File: advs74337‐sup‐0001‐SuppMat.docx. [file ADVS-13-e21997-s001.docx]

**TrxR2** **Lactylation Facilitates Mitochondrial Protection and Endothelial Ferroptosis Resistance in Diabetic Cardiomyopathy**

Su Li^1a^, Muyin Liu^1a^, Chao Chen^2a^, Xinyan Li^3a^, Xiaopei Yan^4^, Wentao Zhu^1^, Wenyan Qiu^1^, Qiyu Li^1^, Xiangyu Sun^5^, Chao Huang^6^, Min Yin^1^, Zhangwei Chen^1^, Yao Lu^7^, Junbo Ge^1^, Xiangqing Kong^2, 8*^, Juying Qian^1*^, Yuqiong Chen^2*^

^a^ These authors contribute equally to this work.

^*^ Yuqiong Chen, Juying Qian, and Xiangqing Kong are corresponding authors.

**Affiliations**

^1^Department of Cardiology, Zhongshan Hospital, Fudan University, Shanghai Institute of Cardiovascular Diseases, National Clinical Research Center for Interventional Medicine, Shanghai 200032, China.

^2^Department of Cardiology, The Affiliated Suzhou Hospital of Nanjing Medical University, Suzhou Municipal Hospital, Gusu School, Nanjing Medical University.

^3^National Cancer Center/National Clinical Research Center for Cancer/Cancer Hospital, Chinese Academy of Medical Sciences and Peking Union Medical College, Beijing, 100021, China

^4^Department of Respiratory Medicine, The Affiliated Suzhou Hospital of Nanjing Medical University, Suzhou Municipal Hospital, Gusu School, Nanjing Medical University

^5^National Cancer Center/National Clinical Research Center for Cancer/Cancer Hospital, Chinese Academy of Medical Sciences and Peking Union Medical College, Beijing, 100021, China

^6^Ministry of Science and Technology, the Affiliated Suzhou Hospital of Nanjing Medical University, Suzhou Municipal Hospital, Suzhou, Jiangsu 215002, China.

^7^XuZhou Clinical School of Xuzhou Medical University, Department of Cardiology, Xuzhou Central Hospital, XuZhou Institute of Cardiovascular disease, No.199 Jiefang South Road, Xuzhou 221009, P.R. China

^8^Department of Cardiology, Gulou District, the First Affiliated Hospital of Nanjing Medical University, 300 Guangzhou Road, Nanjing City, Jiangsu Province, China

**Corresponding authors:**

(1) Yuqiong Chen, MD, PhD

Tel: 86-512-62363800 Fax: 86-512-62363800

Email: [cosmoscyq@163.com](mailto:cosmoscyq@163.com)

Address: No. 80 Tian'edang Road, Wuzhong District, Suzhou, Jiangsu Province, 215000, China

(2) Juying Qian, MD, PhD, FACC, FESC

Tel: 86-21-60268565 Fax: 86-21-64223006

Email: qian.juying@zs-hospital.sh.cn

Address: 180 Fenglin Road, Shanghai 200032, China

(3) Xiangqing Kong, MD, PhD, FESC, FACC

Tel: 86-025-83714511 Fax: 86-025-83724440

Email: [Kongxq@njmu.edu.cn](mailto:Kongxq@njmu.edu.cn)

Address: 300 Guangzhou Road, Gulou District, Nanjing City, Jiangsu Province, China

**Supplementary Figure Legends**

**
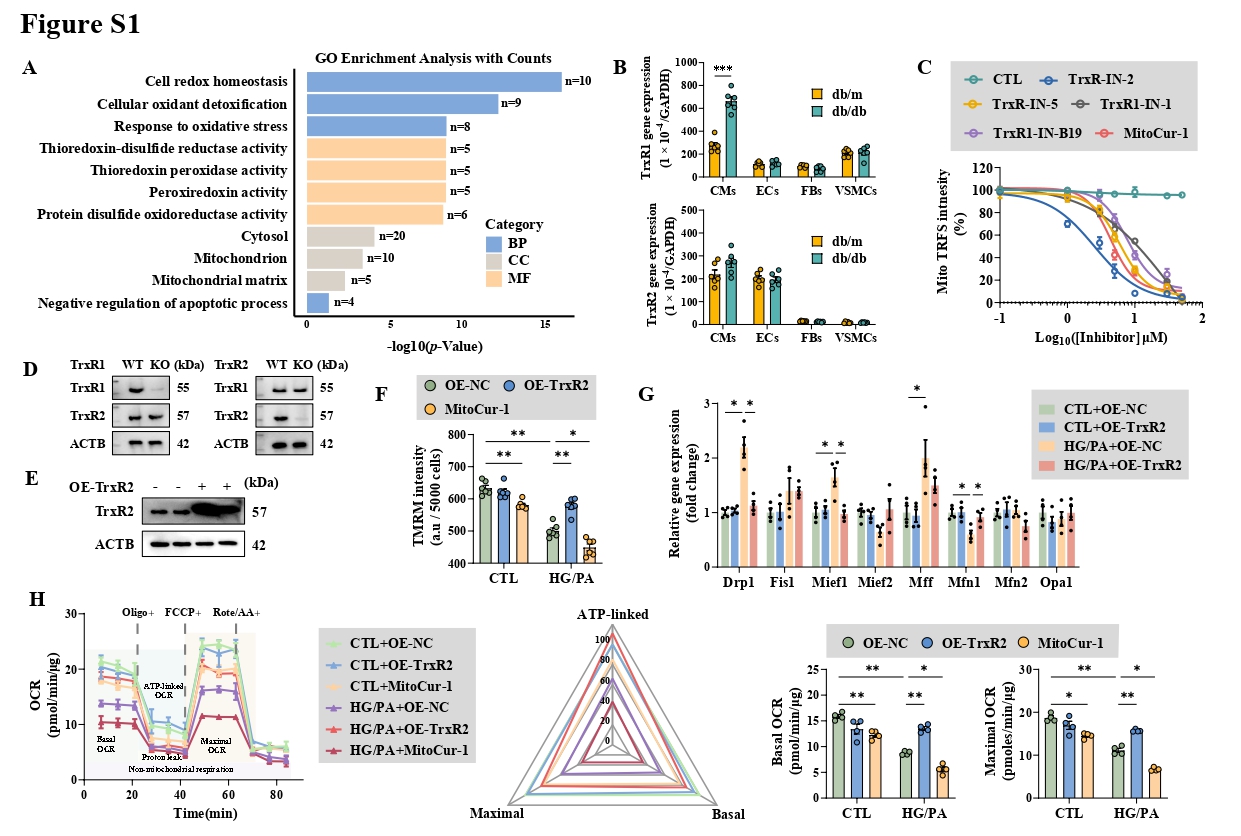
**

**Figure S1. TrxR2 improved mitochondrial dysfunction in HG/PA-injured HCMECs.**

**A**, GO enrichment analysis of thioredoxin family. **B**, *TrxR1* and *TrxR2* mRNA expression in cardiomyocytes (CMs), endothelial cells (ECs), fibroblasts (FBs), and vascular smooth cells (VSMCs) sorted from db/m and db/db mice at 28 weeks of age (n = 6 per group). **C**, Dose-response curve of MitoTRFS intensity following treatment with TrxR inhibitors (n = 3 per group). **D**, TrxR1 and TrxR2 expression was detected by western blot post TrxR1 or TrxR2 knockout. **E**, Western blot validation of TrxR2 overexpression. **F**, Quantification of TMRM intensity (n = 6 per group). **G**, The mRNA expression of mitochondrial dynamics-related genes (n = 4 per group). **H**, Line charts of oxygen consumption rate (OCR, left), radar charts of ATP-linked OCR, basal OCR, and maximal OCR (middle), and bar graphs of basal OCR and maximal OCR (right, n = 4 per group). Unpaired Student’s t-test was used for **B.** Two-way ANOVA, followed by Tukey’s post-hoc multi-comparison test, was used for **C**, **F**, **G** and **H**. *p<0.05, **p<0.01, ***p<0.001, ****p<0.0001 indicate statistically significant differences.


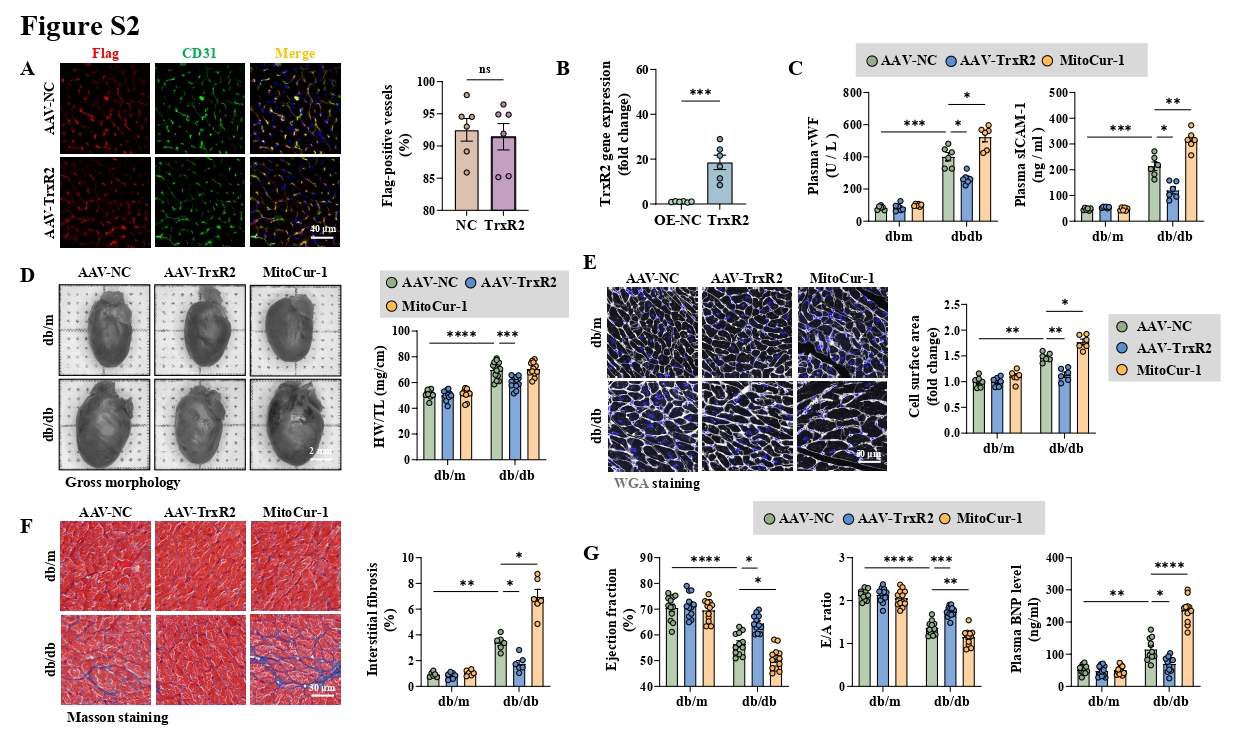


**Figure S2. TrxR2 improved cardiac pathological remodeling and dysfunction in diabetic cardiomyopathy.**

**A** and **B**, Transfection efficiency of AAV9-NC-Flag and AAV9-TrxR2-Flag was assessed by immunofluorescence staining (n = 6 per group); the overexpression efficiency was measured by PCR in primary MCMECs (n = 6 per group). **C**, Statistical analysis of plasma vWF and sICAM-1 levels (n = 6 per group). **D**, Representative images of cardiac morphology and quantification data of HW/TL (heart weight / tibial length) (n = 12 per group). **E**, Representative images of WGA staining with quantification data of relative cell surface area (n = 6 per group). **F**, Representative images of Masson staining and fibrosis quantification (n = 6 per group). **G**, Statistical analysis of ejection fraction, E/A ratio and plasma BNP level (n = 12 per group). Unpaired Student’s t-test was used for **A** and **B**. Two-way ANOVA, followed by Tukey’s post-hoc multi-comparison test, was used for **C-G**. *p<0.05, **p<0.01, ***p<0.001, ****p<0.0001 indicate statistically significant differences. WGA indicates Wheat germ agglutinin.


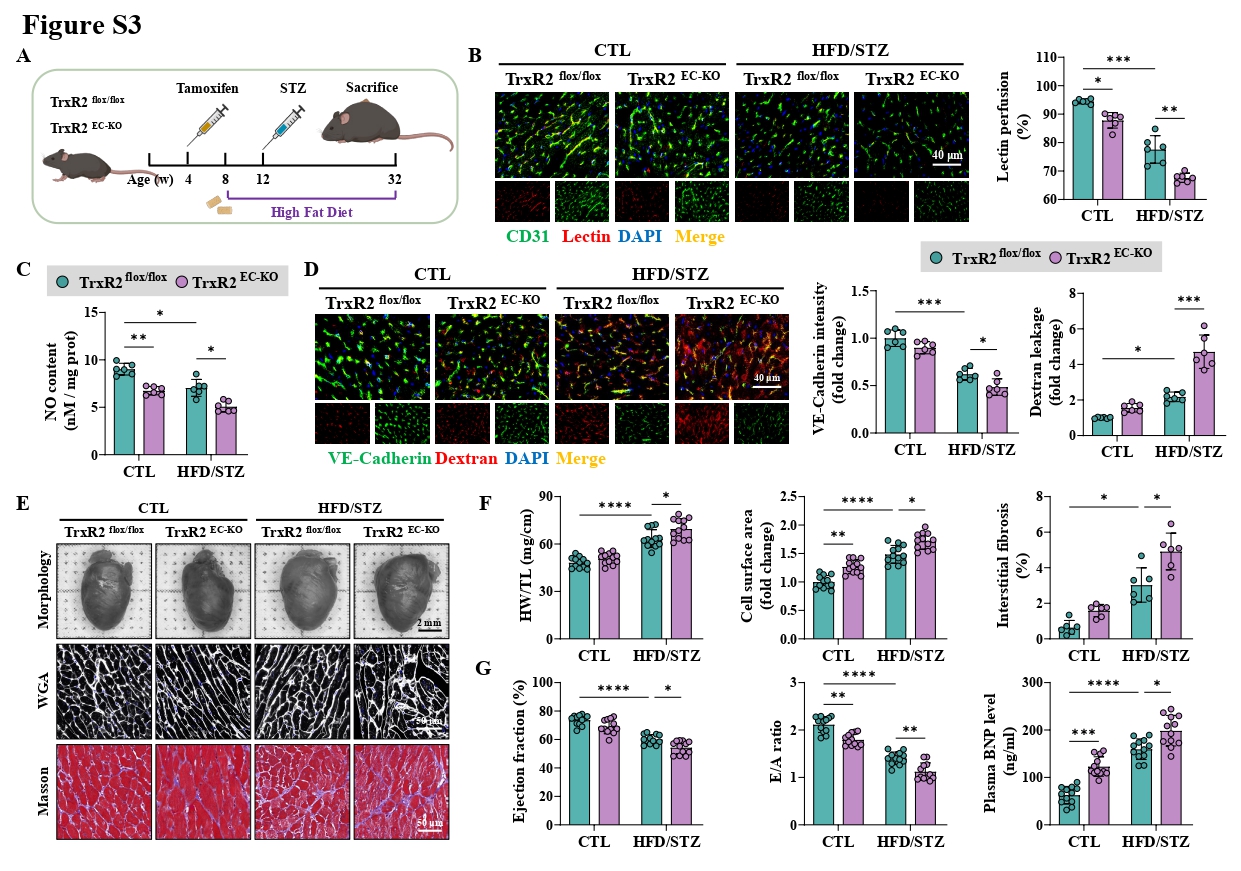


**Figure S3. Endothelial-specific knockout of TrxR2 exacerbated cardiac microvascular injury and cardiac dysfunction in diabetic cardiomyopathy.**

**A**, Schematic diagram illustrating the induction of T2DM in TrxR2^flox/flox^ and TrxR2^EC-KO^ mice using the HFD/STZ method. **B**, Representative immunofluorescence images and quantification of CD31-labeled cardiac microcirculation and Lectin-perfused vessels. (n = 6 per group). **C**, Quantification of cardiac NO content (n=6 per group). **D**, Representative immunofluorescence images and quantification of VE-Cadherin and TRITC-dextran leakage (n = 6 per group). **E**, Representative images of cardiac morphology (top), WGA staining (middle), and Masson staining (bottom). **F**, Quantification data of HW/TL (n = 12 per group), relative cell surface area (n = 12 per group) and fibrosis (n = 6 per group). **G**, Statistical analysis of ejection fraction, E/A ratio and plasma BNP level (n = 12 per group). Two-way ANOVA, followed by Tukey’s post-hoc multi-comparison test, was used for this figure. *p<0.05, **p<0.01, ***p<0.001, ****p<0.0001 indicate statistically significant differences.


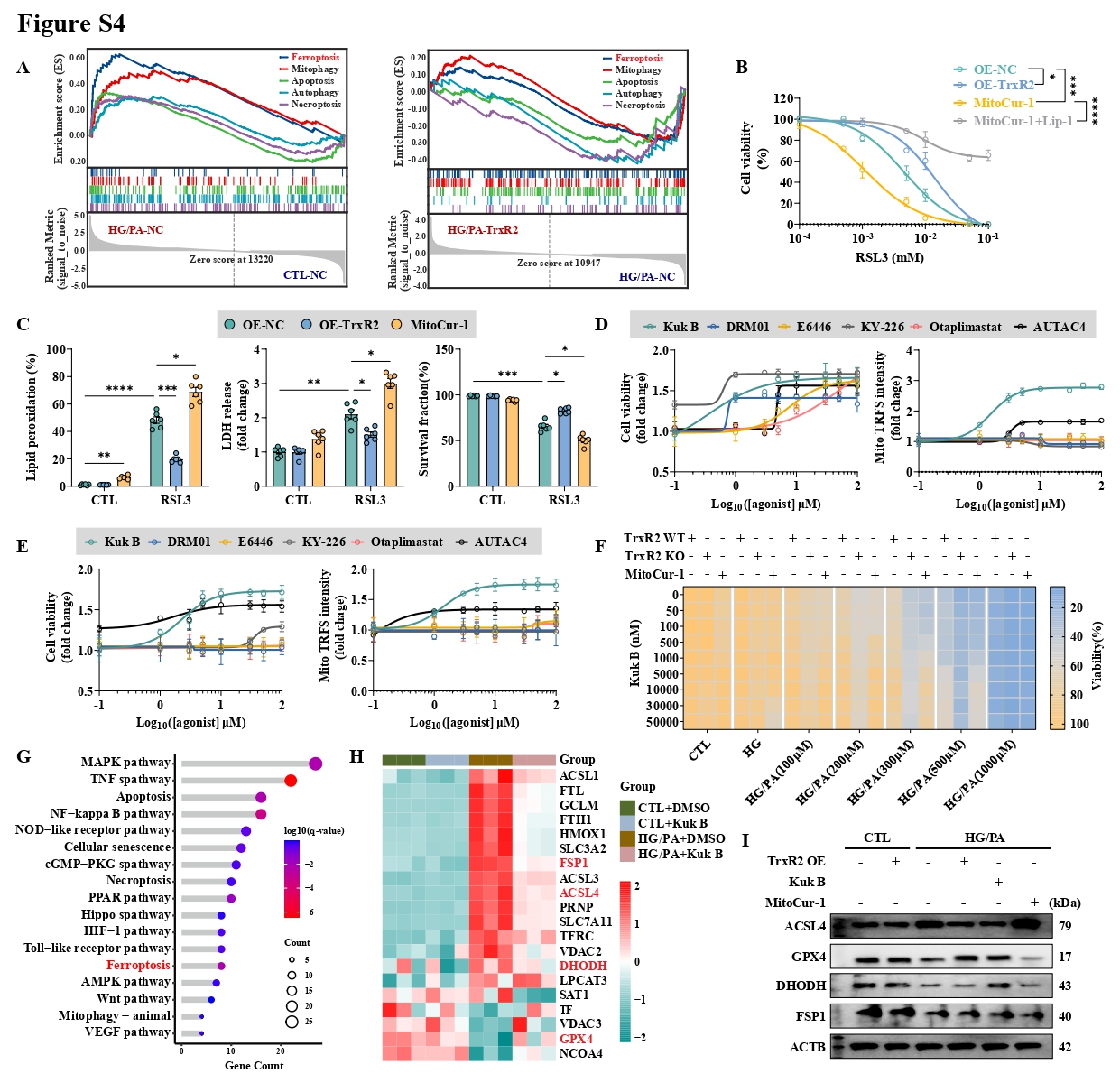


**Figure S4. Kukoamine B was selected as agonist for TrxR2 in suppressing ferroptosis.**

**A**, GO based GSEA rank plot visualization of mitophagy, autophagy, ferroptosis, apoptosis, necroptosis. **B**, Dose-response curves of relative cell viability after 6-hour RSL3 treatment (n = 3 per group). **C**, Statistical analysis of lipid peroxidation (left), LDH release (middle), and survival fraction (right) after RSL3 treatment (n = 6 per group). **D** and **E**, Dose-response curves of potential agonists for relative cell viability and MitoTRFS intensity after 72-hour HG/PA injury (**D**; n = 3 per group) or 6-hour RSL3 treatment (**E**; n = 3 per group) on HCMECs. **F**, HCMECs were treated with Kuk B and subjected to HG/PA injury at the indicated concentrations for 72 hours. Relative cell viability was summarized in a heatmap. **G**, KEGG enrichment analysis of DEGs between the HG/PA+DMSO and HG/PA+Kuk B groups. **H**, Heatmap of ferroptosis genes in indicated groups. **I**, Western blot of ACSL4, GPX4, DHODH and FSP1 expression in indicated groups (n = 3 per group). Two-way ANOVA, followed by Tukey’s post-hoc multi-comparison test, was used for this figure. *p<0.05, **p<0.01, ***p<0.001, ****p<0.0001 indicate statistically significant differences.


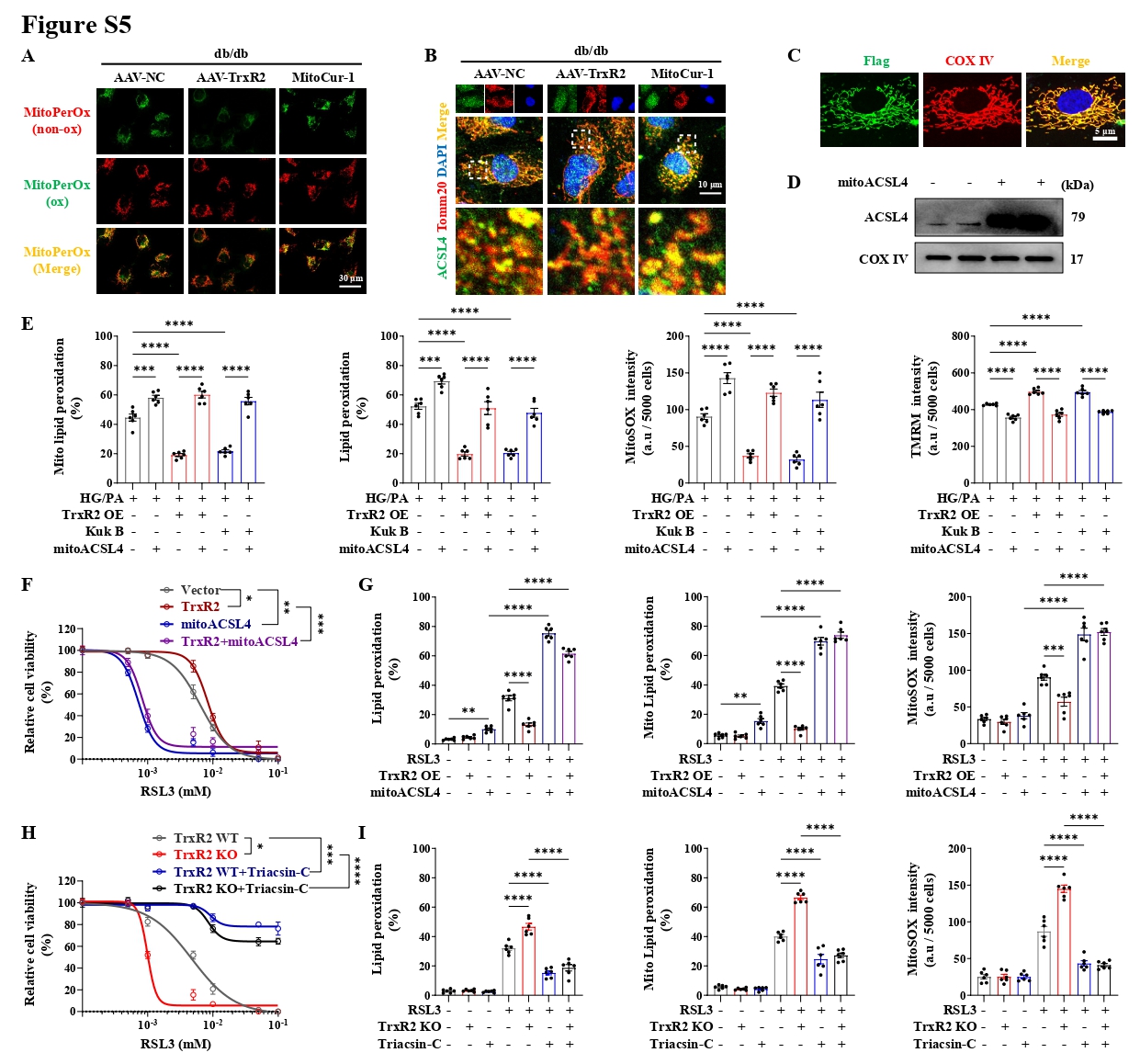


**Figure S5. TrxR2 reduced mitochondrial lipid peroxidation and alleviated mitochondrial dysfunction via inhibiting mitochondrial translocation of ACSL4.**

**A** and **B**, Representative immunoﬂuorescence images of MitoPerOx staining and mitoACSL4 in primary MCMECs sorted from indicated mice. **C**, Representative immunoﬂuorescence images of Flag-mitoACSL4 transfection. **D**, The transfection efficiency of mitoACSL4 was detected by western blot (n = 2 per group). **E**, After TrxR2 and/or mitoACSL4 overexpression, HCMECs were subjected to HG/PA injury for 72 hours, with or without Kuk B (10 μM) treatment. The mitoLPO level, LPO level, MitoSOX intensity, and TMRM intensity were statistically analyzed (n = 6 per group). **F**, Dose-response curves of relative cell viability after 6- hour RSL3 treatment (n = 3 per group). **G**, After TrxR2 and/or mitoACSL4 overexpression, HCMECs were subjected to 10 μM RSL3 treatment for 6 hours. The mitoLPO level, LPO level, and MitoSOX intensity were statistically analyzed (n = 6 per group). **H**, Dose-response curves of relative cell viability after 6-hour RSL3 treatment in TrxR2 WT and TrxR2 KO cell lines, with or without 24-hour Triacsin-C (1 μM) pretreatment (n = 3 per group). **I**. TrxR2 WT and TrxR2 KO HCMECs were subjected to 10 μM RSL3 treatment for 6 hours, with or without 24 hours Triacsin-C (1 μM) pretreatment. The mitoLPO level, LPO level, and MitoSOX intensity were statistically analyzed (n = 6 per group). Two-way ANOVA, followed by Tukey’s post-hoc multi-comparison test, was used for this figure. *p<0.05, **p<0.01, ***p<0.001, ****p<0.0001 indicate statistically significant differences.


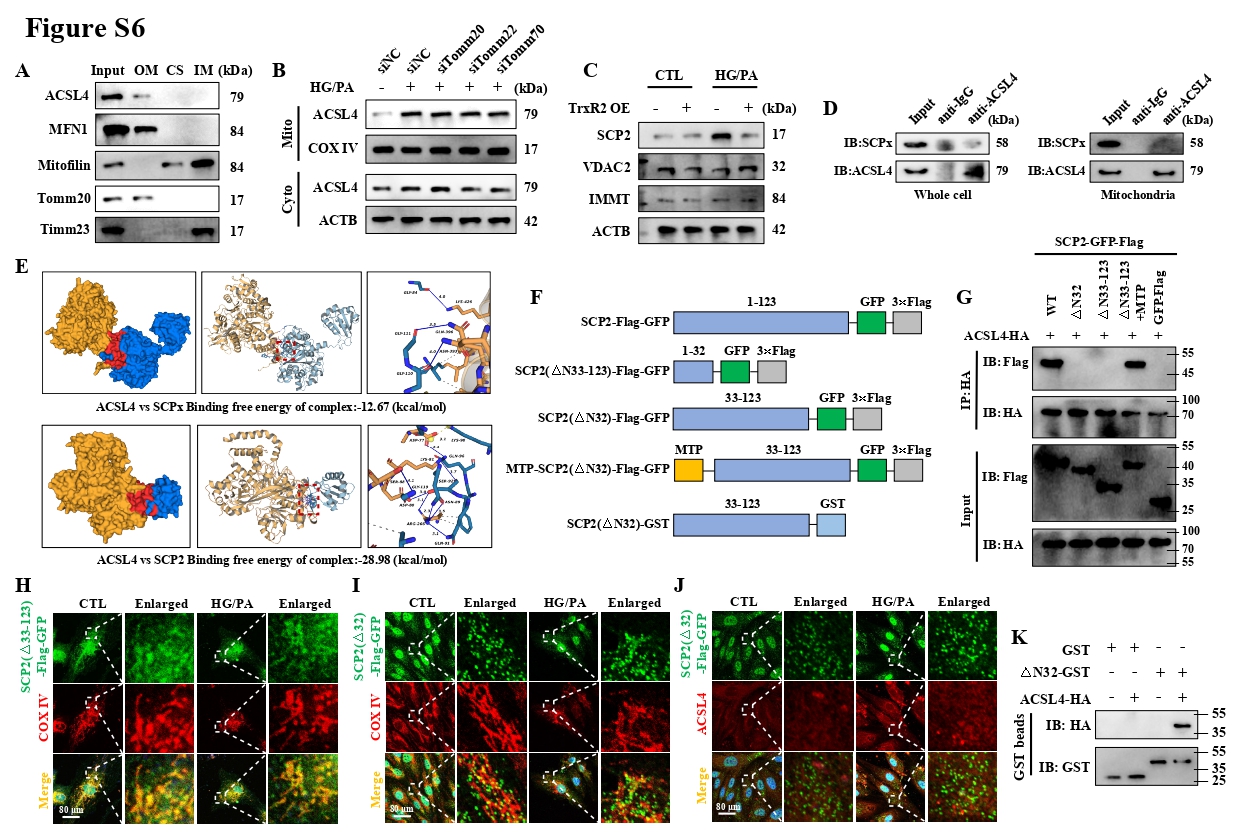


**Figure S6. SCP2 acted as a mitochondrial receptor for ACSL4 through its non-specific lipid combination region.**

**A**, Western blot of ACSL4, MFN1, Mitofilin, Tomm20, and Timm23 expression in the mitochondrial outer membrane (OM), contact site (CS) and inner membrane (IM) (n = 2 per group). **B**, Western blot analysis of ACSL4 expression in the mitochondrial fraction and mitochondria-free fraction following knockdown of Tomm20, Tomm22, and Tomm70 (n = 2 per group). **C**, Western blot analysis of SCP2, VDAC2, and IMMT expression in the indicated groups (n = 3 per group). **D**, The interactions between ACSL4 and SCPx were analyzed by Co-IP assay in whole-cell lysate and mitochondrial lysate (n = 2 per group). **E**, The predicted docking models and binding free energy values for ACSL4 with SCP2 or SCPx were analyzed. **F**, A schematic diagram presenting the strategies for SCP2 truncated mutants. **G**, The interactions between HA-tagged ACSL4 and Flag-tagged SCP2 truncated mutants were detected by Co-IP assay in whole cell lysates (n = 2 per group). **H-J**. Representative immunoﬂuorescence images of Flag-tagged SCP2 truncated mutants, COX IV and ACSL4 staining in HCMECs, with or without HG/PA injury. **K**, The combination between GST-tagged SCP2△N32 and HA-tagged ACSL4 was detected by GST-pulldown assay (n = 2 per group).


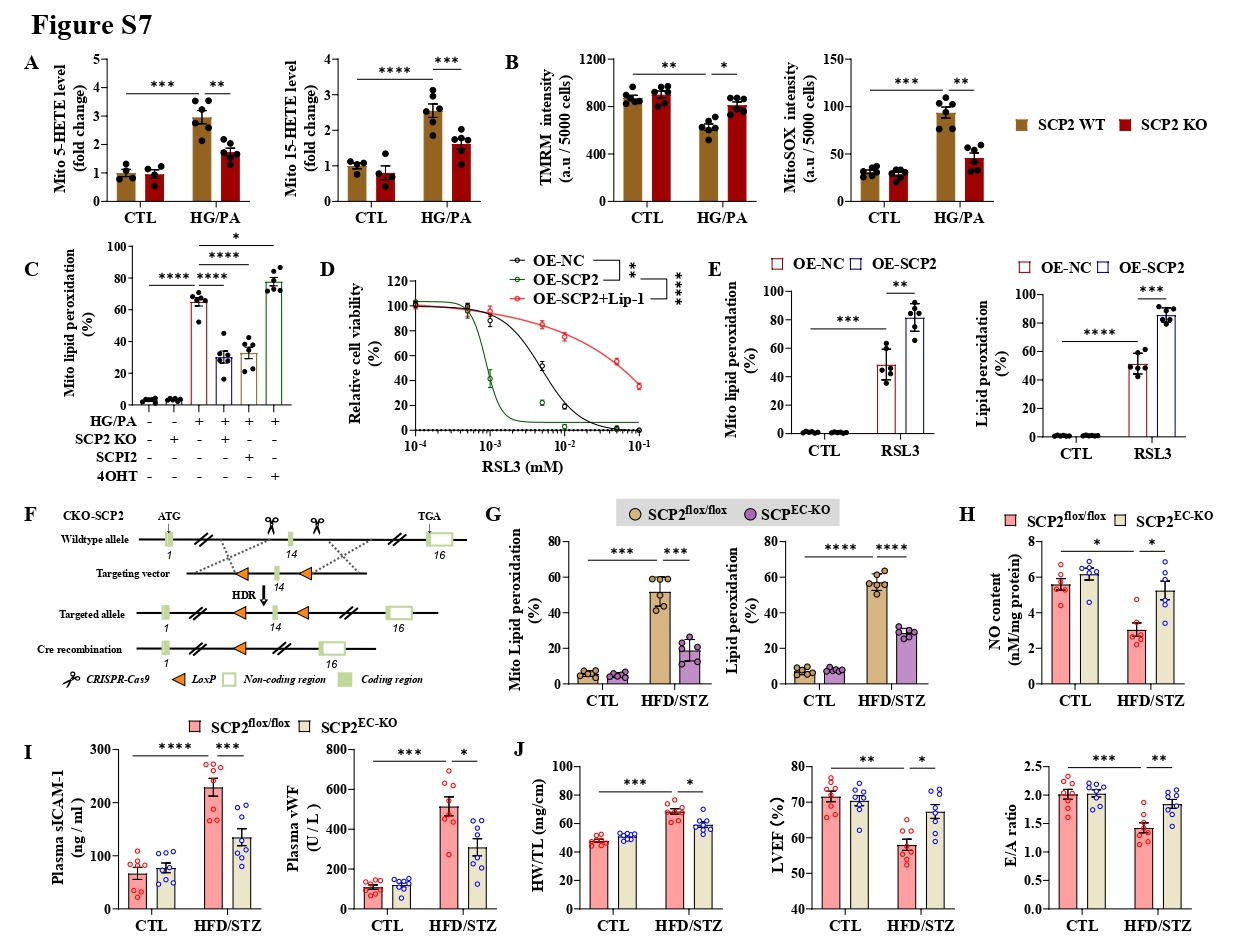


**Figure S7. Endothelial-specific knockout of SCP2 inhibited endothelial ferroptosis and ameliorated microvascular injury in diabetic cardiomyopathy.**

**A**, Mitochondrial 5-HETE and 15-HETE levels were measured using ELISA and statistically analyzed (n = 6 per group). **B**, Quantification of TMRM intensity and MitoSOX intensity (n = 6 per group). **C**, mitoLPO level was statistically analyzed, with or without pretreatment of SCP2 agonist (4OHT, 1 μM) and inhibitor (SCPI2, 5 μM) for 24 hours (n = 6 per group). **D**, Dose-response curves of relative cell viability after 6-hour RSL3 treatment in indicated groups. **E**, After SCP2 overexpression, HCMECs were subjected to 10 μM RSL3 treatment for 6 hours (n = 6 per group). The mitoLPO and LPO levels were statistically analyzed (n = 6 per group). **F**, The strategy for generating endothelial-specific SCP2 knockout (SCP2^EC-KO^) mice. **G**, Quantification data of mitoLPO and cellular LPO from MitoPerOx staining and BODIPY C11 staining in **Figure 4J** (n = 6 per group). **H**, Cardiac NO content was analyzed (n = 6 per group). **I**, Plasma vWF and sICAM-1 levels were analyzed (n=8 per group). **J**, Statistical analysis of HW/TL, LVEF, and E/A ratio (n = 8 per group). Two-way ANOVA, followed by Tukey’s post-hoc multi-comparison test, was used for this figure. *p<0.05, **p<0.01, ***p<0.001, ****p<0.0001 indicate statistically significant differences.


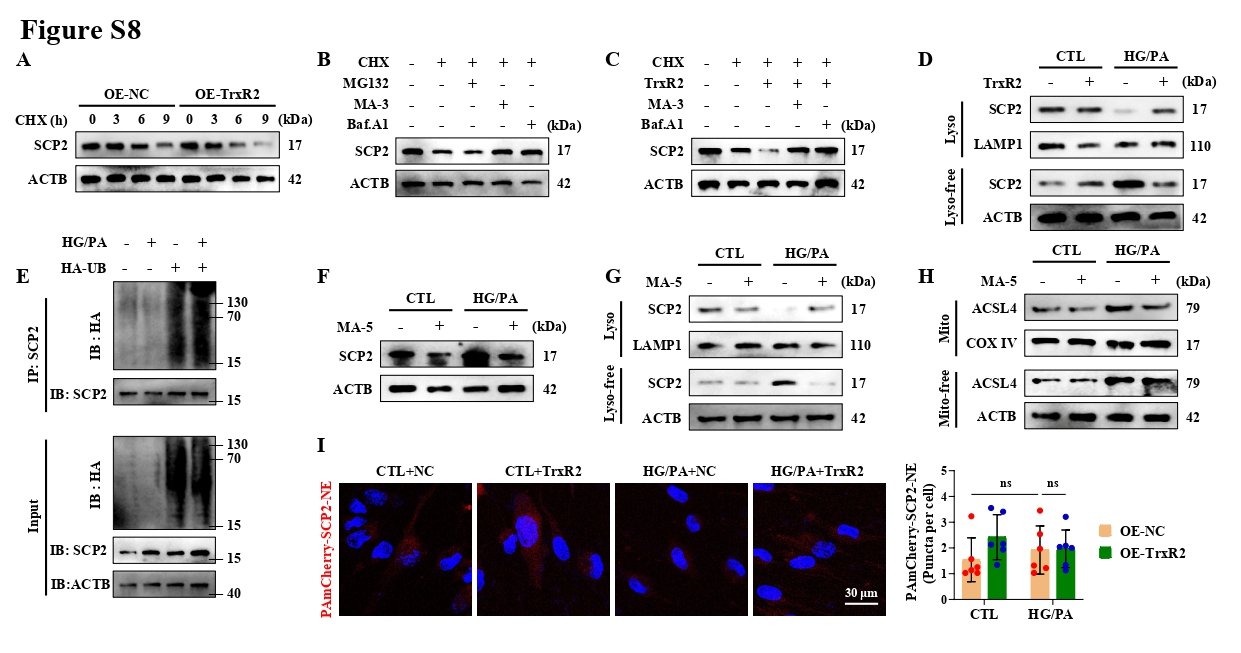


**Figure S8.** **SCP2 was degraded through the autophagy pathway.**

**A**, SCP2 expression was analyzed by western blot after Cycloheximide (CHX, 100 μM) treatment at indicated time points, with or without TrxR2 overexpression (n = 3 per group). **B** and **C**, SCP2 expression was assessed by western blot following 6-hour treatment with CHX(100 μM), MG132 (10 μM), 3-Methyladenine (MA-3, 5 μM), and Bafilomycin A1 (Baf.A1, 1 μM), with or without TrxR2 overexpression (n = 3 per group). **D**, SCP2 expression in lysosomal fraction and lysosome-free fraction was detected by western blot (n = 3 per group). **E**, HCMECs were transfected with HA-Ub (ubiquitin) before the cell lysates were immunoprecipitated with anti-SCP2 antibody. Immunoprecipitants and input were analyzed by western blot (n = 2 per group). **F-H**, Mitochonic acid 5 (MA-5, 5 μM, 72 hours) increased lysosomal degradation of SCP2 and reduced mitoACSL4 (n=2 per group). **I**, Representative immunofluorescence images and statistical analysis of PAmCherry-SCP2-NE staining for chaperone-mediated autophagy (CMA) degradation of SCP2 (n = 6 per group). Two-way ANOVA, followed by Tukey’s post-hoc multi-comparison test, was used for this figure. ns indicates not significant.


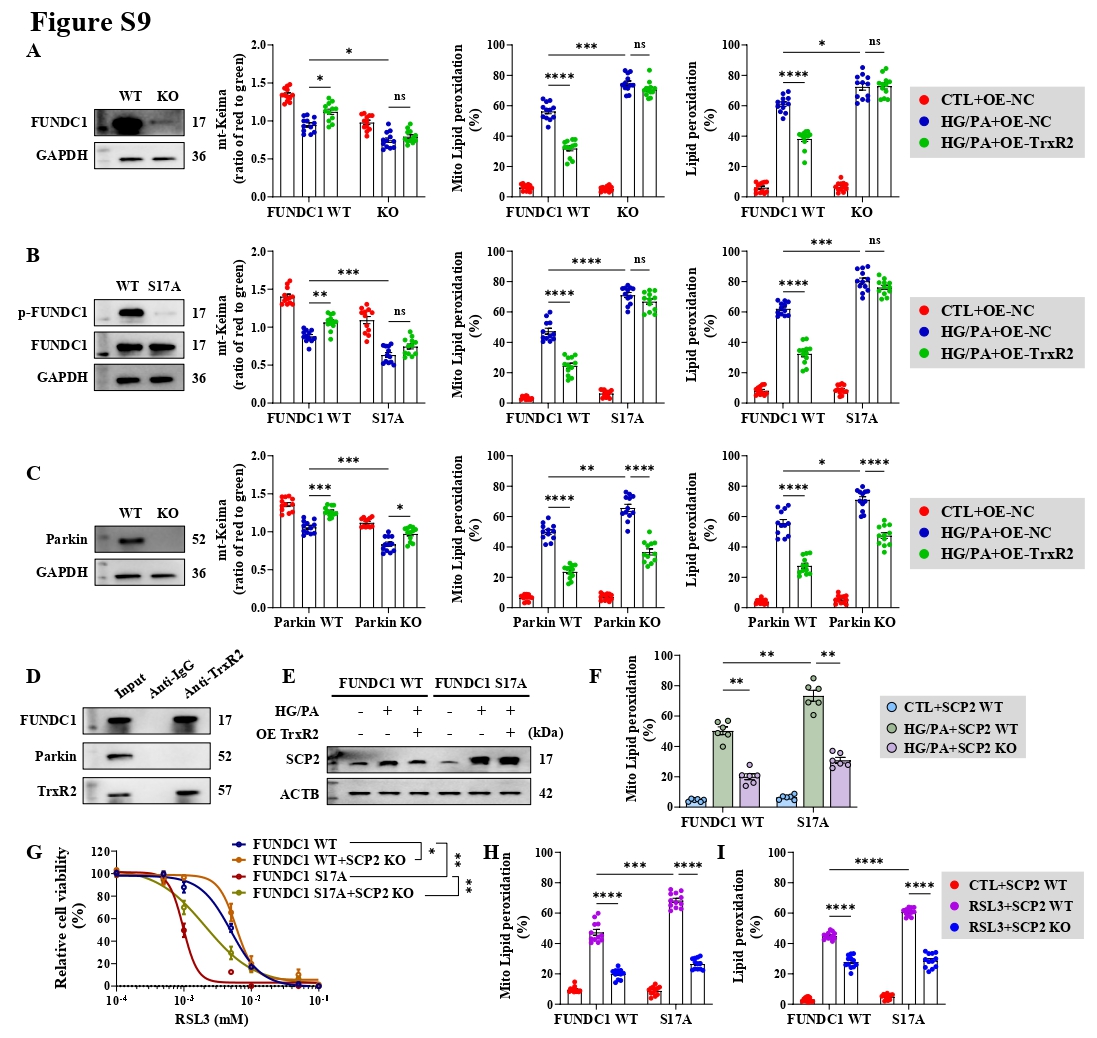


**Figure S9. SCP2 was degraded through FUNDC1-mitophagy pathway.**

**A-C**, Statistical analysis of mt-Keima, mitoLPO and cellular LPO in wild type, FUNDC1 KO, FUNDC1 S17A, and Parkin KO cell lines (n = 12 per group). **D**, The interactions among FUNDC1, Parkin and TrxR2 were detected by Co-IP assay (n = 2 per group). **E**, Western blot analysis of SCP2 expression in FUNDC1 WT and FUNDC1 S17A cell lines following HG/PA treatment and TrxR2 overexpression (n = 2 per group). **F**, Statistical analysis of mitochondrial lipid peroxidation in FUNDC1 WT and FUNDC1 S17A cell line, with or without SCP2 KO (n = 6 per group). **G**, Dose-response curves for relative cell viability after 6-hour RSL3 treatment (n = 3 per group). **H** and **I**, Statistical analysis of mitoLPO and cellular LPO after 6-hour RSL3 (10 μM) treatment in indicated cell lines (n = 12 per group). Two-way ANOVA, followed by Tukey’s post-hoc multi-comparison test, was used for this figure. *p<0.05, **p<0.01, ***p<0.001, ****p<0.0001 indicate statistically significant differences.


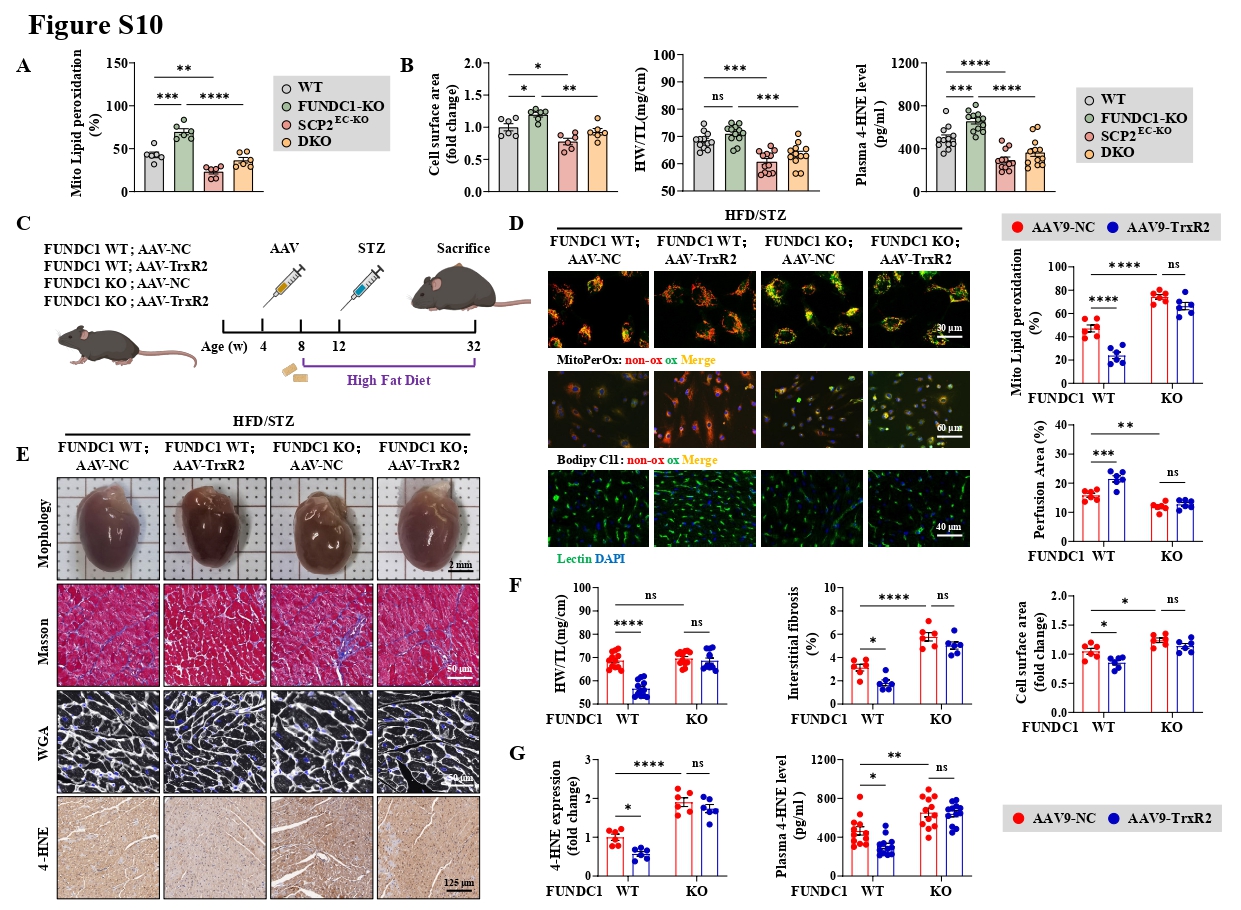


**Figure S10. Knockout of FUNDC1 abolished the benefits of TrxR2 on diabetic cardiomyopathy.**

**A**, Quantification of mitoLPO (n = 6 per group) in indicated groups. **B**, Statistical analysis of relative cell surface area (n = 6 per group), HW/TL (n = 12 per group) and plasma 4-HNE (n = 12 per group) in indicated groups. **C**, Schematic outline demonstrating the injection of AAV-NC and AAV-TrxR2 into FUNDC1 WT and FUNDC1 KO mice, followed by the induction of T2DM using the HFD/STZ method. **D**, Representative immunofluorescence images of MitoPerOx and BODIPY C11 staining in primary MCMECs, along with Lectin-perfused vessels in the indicated groups (n = 6 per group). **E**, Representative images of cardiac morphology, WGA staining, Masson staining, and 4-HNE staining in the indicated groups (n = 6 per group). **F**, Statistical analysis of HW/TL (n = 12 per group), interstitial fibrosis (n = 6 per group), and relative cell surface area (n = 6 per group). **G**, Statistical analysis of cardiac (n = 6 per group) and plasma (n = 12 per group) 4-HNE level in indicated groups. Two-way ANOVA, followed by Tukey’s post-hoc multi-comparison test, was used for this figure. *p<0.05, **p<0.01, ***p<0.001, ****p<0.0001 indicate statistically significant differences.


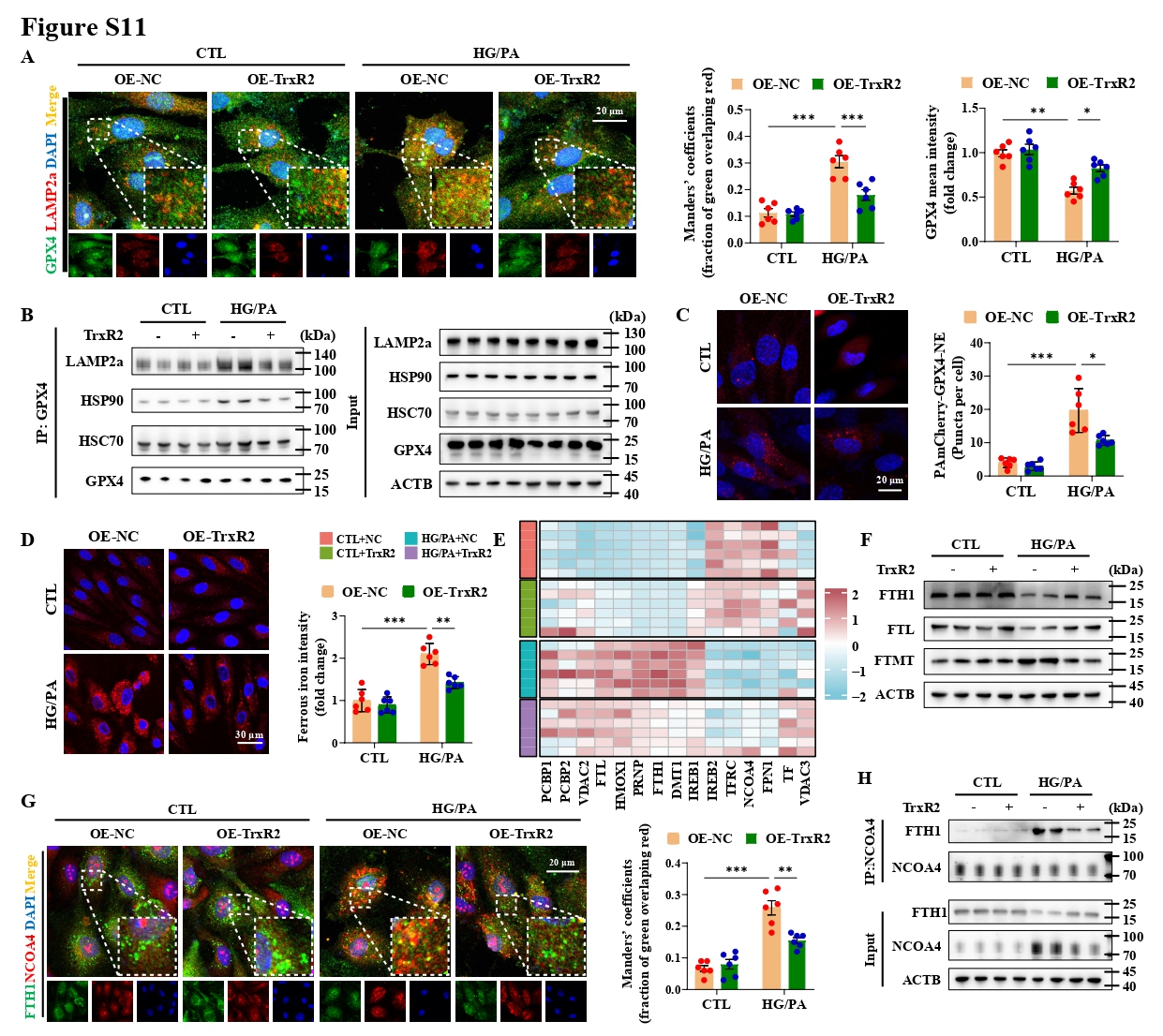


**Figure S11. TrxR2 inhibited the chaperone-mediated autophagy degradation of GPX4 and ferritinophagy.**

**A**, Representative immunofluorescence images and statistical analysis of GPX4 and LAMP2a colocalization using Manders’ coefficients (n = 6 per group). **B**, The interactions between GPX4 and chaperones (HSC70, HSP90 and LAMP2a) were detected by Co-IP assay (n = 4 per group). **C**, Representative immunofluorescence images and quantification of PAmCherry-GPX4-NE staining for CMA degradation of GPX4 (n = 6 per group). **D**, Representative immunoﬂuorescence images and quantification of ferrous iron intensity (n = 6 per group). **E**, Heatmap of iron transport and ferritinophagy-related genes from RNA-sequencing assay. **F**, Western blot analysis of FTH1, FTL, and FTMT protein expression (n = 3 per group). **G**, Representative immunofluorescence images and statistical analysis of FTH1 and NCOA4 colocalization using Manders’ coefficients (n = 6 per group). **H**, The combination between FTH1 and NCOA4 were detected by Co-IP assay (n = 4 per group). Two-way ANOVA, followed by Tukey’s post-hoc multi-comparison test, was used for this figure. *p<0.05, **p<0.01, ***p<0.001, ****p<0.0001 indicate statistically significant differences.


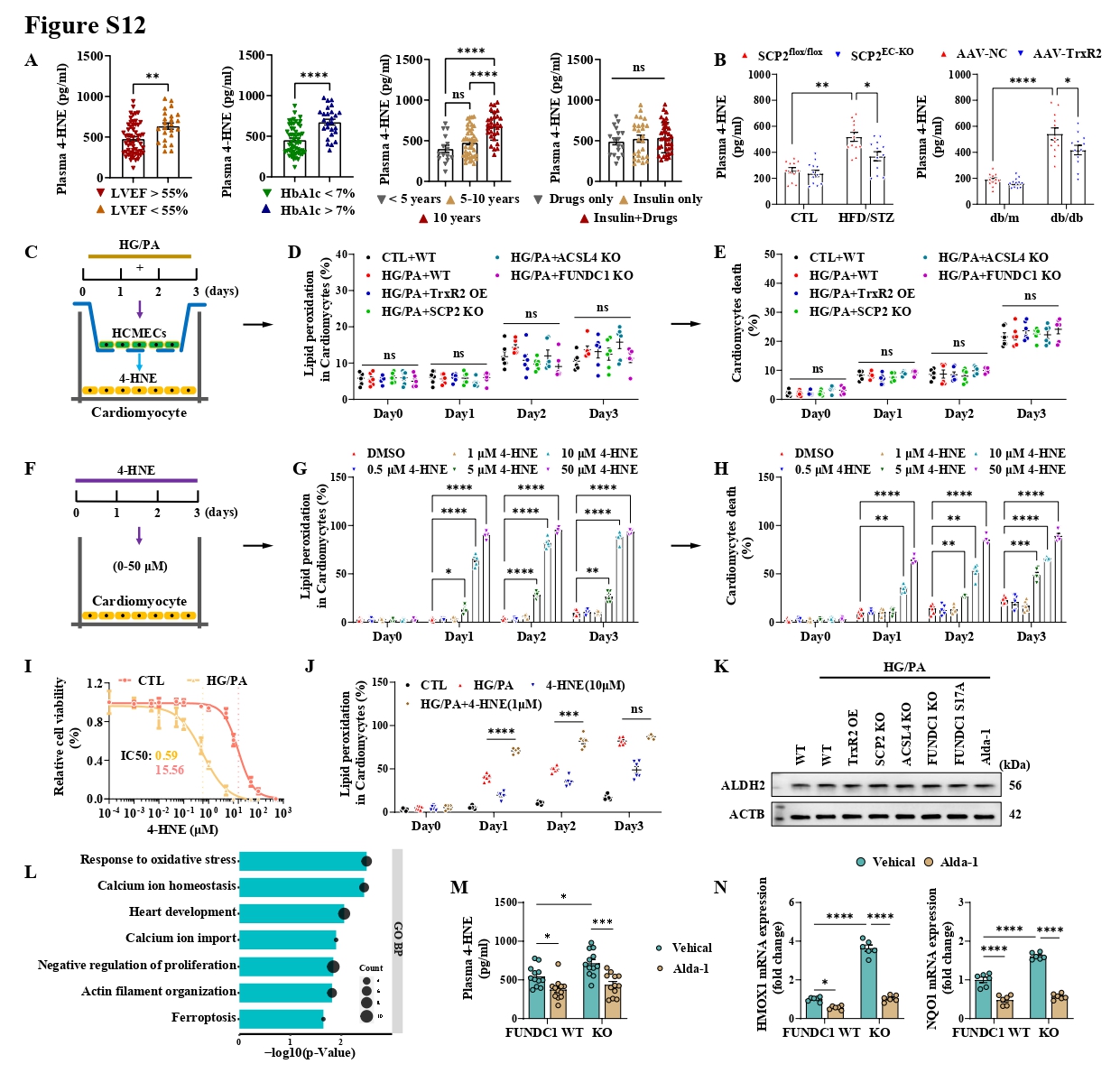


**Figure S12. ECs-released 4-HNE facilitated cardiomyocytes ferroptosis under diabetic injury.**

**A**, Subgroup analyses of plasma 4-HNE levels in diabetic patients. **B**, Statistical analysis of plasma 4-HNE levels in the indicated mice (n = 12 per group). **C**, The illustration demonstrates that 4-HNE was released from HCMECs in the upper chamber to cardiomyocytes in the lower chamber. **D** and **E**, Genetically edited HCMECs were exposed to HG/PA injury in the upper chamber; LPO level and cell death of cardiomyocytes in the lower chamber were statistically analyzed (n = 6 per group). **F-H**, 4-HNE was directly administered to cardiomyocytes; LPO levels and cell death of cardiomyocytes were statistically analyzed (n = 6 per group). **I**, Dose-response curves of 4-HNE for cell viability, with or without HG/PA injury (n = 6 per group). IC50 indicates inhibitory concentration 50%. **J**, Statistical analysis of LPO levels in cardiomyocytes in indicated groups (n = 6 per group). **K**, Western blot analysis of ALDH2 expression in the indicated groups was performed (n = 2 per group). **L**, GO enrichment analysis was performed on the 222 co-regulated DEGs identified in Figure 6J. **M** and **N**, Statistical analysis of plasma 4-HNE level (n = 12 per group) and mRNA expression of *HMOX1* and *NQO1* in primary cardiomyocytes (n = 6 per group) in indicated mice. Two-way ANOVA, followed by Tukey’s post-hoc multi-comparison test, was used for this figure. *p<0.05, **p<0.01, ***p<0.001, ****p<0.0001 indicate statistically significant differences.


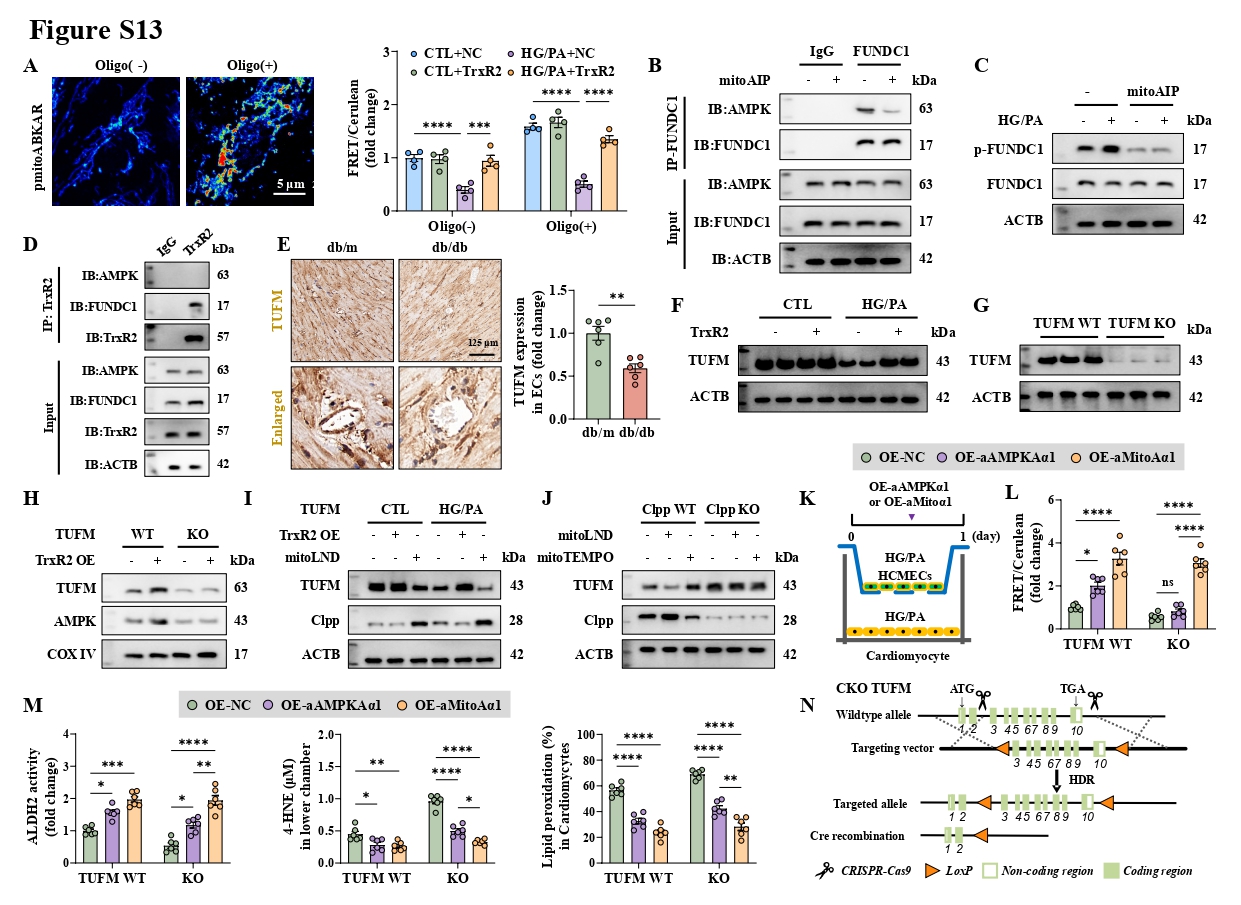


**Figure S13. TrxR2 increased TUFM expression via suppressing oxidative stress and Clpp.**

**A**, Representative images of pmitoABKAR transfection, and quantification of mitoAMPK activity were presented as normalized FRET ratios (n = 4 per group). **B**, The interaction between FUNDC1 and AMPK was detected using Co-IP assay (n = 2 per group). **C**, Western blot analysis of FUNDC1 phosphorylation at Ser17 was performed, with or without mitoAIP transfection (n = 2 per group). **D**, The interaction among FUNDC1, AMPK and TrxR2 was detected by Co-IP assay (n = 2 per group). **E**, Representative images of TUFM staining and statistical analysis of TUFM expression in endothelium (n = 6 per group). **F** and **G**, Western blot analysis was performed to assess TUFM expression in the indicated groups (n = 4 per group). **H**, Western blot analysis of TUFM and AMPK expression in mitochondrial fraction (n = 2 per group). **I** and **J**, Western blot analysis was performed to assess the expression of TUFM and Clpp after treatment with TrxR2 overexpression, the ROS agonist mitoLND, or the ROS inhibitor mitoTEMPO (n = 3 per group). **K**, An illustration depicts that HCMECs were transfected with aAMPKα1 or aMitoAα1 and subsequently subjected to HG/PA injury in the co-culture system. **L** and **M**, Endothelial mitoAMPK activity and ALDH2 activity, ECs-released 4-HNE levels, and LPO levels in cardiomyocytes in the co-culture system were statistically analyzed (n = 6 per group). **N**, The strategy for constructing endothelial-specific TUFM knockout (TUFM^EC-KO^) mice. Unpaired Student’s t-test was used for **E**. Two-way ANOVA, followed by Tukey’s post-hoc multi-comparison test, was used for **A**, **L**, and **M**. *p<0.05, **p<0.01, ***p<0.001, ****p<0.0001 indicate statistically significant differences.


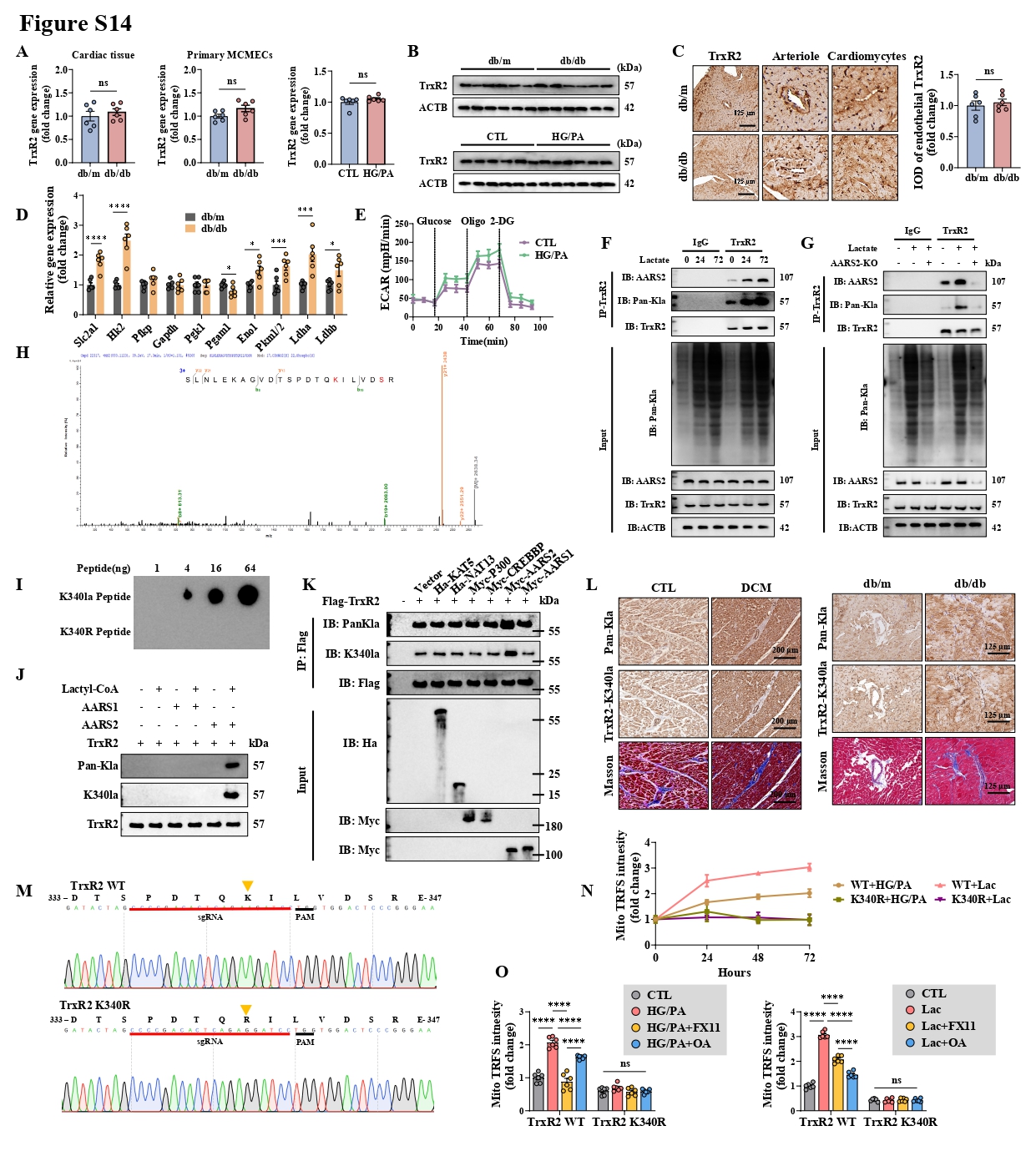


**Figure S14. K340 was identified as the residue for TrxR2 lactylation modification.**

**A**, Quantification of *TrxR2* mRNA expression was performed in cardiac tissue, primary MCMECs, and HG/PA-injured HCMECs (n = 6 per group). **B**, Western blot of TrxR2 protein expression in cardiac tissue and HG/PA-injured HCMECs (n = 6 per group). **C**, Representative image of TrxR2 staining and statistical analysis of endothelial TrxR2 expression (n = 6 per group). **D**, The statistical analysis of glycolytic gene expression from PCR in sorted primary MCMECs (n=6 per group). **E**, The extracellular acidification rate (ECAR) curve was analyzed following HG/PA injury (n = 4 per group). **F** and **G**, The effects of L-lactate (30mM) treatment (F) and AARS2 knockout (G) on pan-lactylation of cell lysates and TrxR2 were analyzed by Co-IP assay (n = 2 per group). **H**, The K340 were identified as the lactylation site of TrxR2 using mass spectrometry. **I**, The reactivity and specificity of the custom-made TrxR2 K340la antibody were validated using dot blot. **J**, In vitro lactylation assays using purified AARS1, AARS2, and TrxR2 proteins to verify direct catalysis (n = 2 per group). **K**, HCMECs was co-transfected with TrxR2 and candidate lactyltransferases. TrxR2 lactylation was detected by western blot (n = 2 per group). **L**, Representative images of pan-lactylation staining, TrxR2 K340la staining, and Masson staining in DCM (left) and db/db mice (right) (n = 3 per group). **M**, Sanger sequencing of genotyping for TrxR2 WT and TrxR2 K340R HCMECs. **N**, Time course analysis of MitoTRFS intensity after HG/PA injury and lactate sodium treatment in TrxR2 WT and K340R cell lines (n = 3 per group). **O**, Statistical analysis of MitoTRFS intensity was performed following 72 hours of HG/PA injury (left) or sodium lactate treatment (right), with or without LDH inhibitors (10 μM FX-11 and 10 μM OA) treatment (n = 6 per group). Unpaired Student’s t-test was used for **A**, **C** and **D.** Two-way ANOVA, followed by Tukey’s post-hoc multi-comparison test, was used for **E, N, and O**. *p<0.05, **p<0.01, ***p<0.001, ****p<0.0001 indicate statistically significant differences. ECAR indicates extracellular acidification rate; FX-11, LDHA Inhibitor FX11; OA, Oxamic acid.


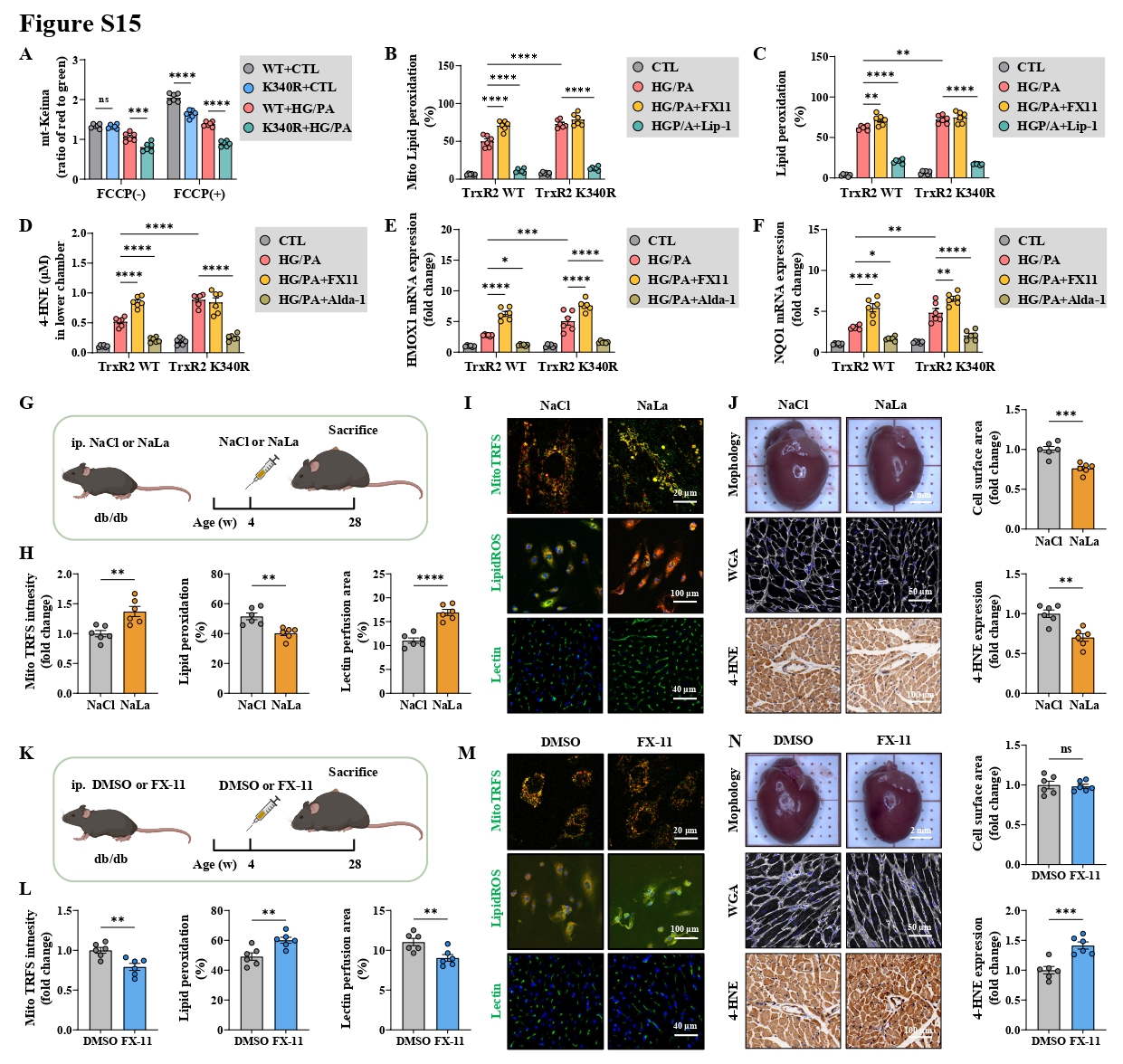


**Figure S15. Lactate and TrxR2 lactylation offered cardiovascular benefits in diabetic cardiomyopathy.**

**A**, Quantification data of mitophagy level in TrxR2 WT and K340R HCMECs (n = 6 per group). **B** and **C**, Statistical analysis of mitoLPO and LPO level in TrxR2 WT and K340R HCMECs, with or without FX-11 and Lip-1 (Liproxstatin-1) treatment (n = 6 per group). **D-F**, TrxR2 WT and K340R HCMECs were seeded in the upper chamber of the co-culture system. The 4-HNE levels in the lower chamber, as well as the *HMOX1* and *NQO1* mRNA levels in cardiomyocytes, were statistically analyzed (n = 6 per group). **G**, The schematic outline illustrates that diabetic db/db mice were treated with sodium lactate (NaLa, 50 mg/kg/day) or an equal volume of sodium chloride (NaCl) for 24 weeks. **H** and **I**, Representative fluorescence images and quantification of MitoTRFS staining and BODIPY C11staining in primary MCMECs, as well as the lectin perfusion assay (n = 6 per group). **J**, Representative images and quantification data of cardiac morphology (top), WGA staining (middle), and 4-HNE staining (bottom) (n=6 per group). **K**, Schematic outline showed diabetic db/db mice were treated with FX-11 (2 mg/kg per day) or DMSO control for 24 weeks. **L** and **M**, Representative fluorescence images and quantification of MitoTRFS staining and BODIPY C11 staining in primary MCMECs, as well as the lectin perfusion assay (n = 6 per group). **N**, Representative images and quantification data of cardiac morphology (top), WGA staining (middle), and 4-HNE staining (bottom) (n = 6 per group). Unpaired Student’s t-test was used for **G-N**. Two-way ANOVA, followed by Tukey’s post-hoc multi-comparison test, was used for **A-F**. *p<0.05, **p<0.01, ***p<0.001, ****p<0.0001 indicate statistically significant differences.
